# Robust $\ell _1$ Approaches to Computing the Geometric Median and Principal and Independent Components

**DOI:** 10.1007/s10851-016-0637-9

**Published:** 2016-02-24

**Authors:** Stephen L. Keeling, Karl Kunisch

**Affiliations:** Institut für Mathematik und Wissenschaftliches Rechnen, Karl-Franzens-Universität Graz, Heinrichstraße 36, 8010 Graz, Austria

**Keywords:** Geometric median, principal component analysis, Independent component analysis, Robustness, Local convergence of iterative methods

## Abstract

Robust measures are introduced for methods to determine statistically uncorrelated or also statistically independent components spanning data measured in a way that does not permit direct separation of these underlying components. Because of the nonlinear nature of the proposed methods, iterative methods are presented for the optimization of merit functions, and local convergence of these methods is proved. Illustrative examples are presented to demonstrate the benefits of the robust approaches, including an application to the processing of dynamic medical imaging.

## Introduction

The topics of this work focus on the low-dimensional representation of complex measured data. The lowest dimensional representation is a type of average. More accurate representations add dimensions beyond the average based upon subspaces in which the data vary the most. Choosing a basis for such subspaces is driven by the priority that data coordinates with respect to this basis be statistically uncorrelated or even statistically independent. The particular interest here is to present methods for performing these tasks which are robust against outliers in the measured data.

The most common type of average is the mean, which may be formulated variationally as the point minimizing the sum of squared distances to data points. As discussed in [14], a more robust method involves minimizing a merit function which does not grow as rapidly with respect to the data and would therefore apply less weight to erroneous data points far from a natural average. Various notions of an average based upon $\ell _1$ measures are discussed in [11]. Based upon examples presented in Sect. 3, the type of average selected for this work is the *geometric median*, which may be formulated variationally as the point minimizing the sum of distances (not squared) to data points. The problem of determining the geometric median has a long history. In the 1937 paper by Weiszfeld [21], three proofs concerning the uniqueness of the geometric median are given, and one of these supplies an algorithm for its computation. We also refer to the recent annotated translation of that paper [19]. See also [3]. A shorter proof of uniqueness is given in Sect. 6 which is based upon a strict convexity argument. Moreover, a possibly novel characterization of a solution is provided in case data points are collinear. A regularized Weiszfeld iteration for computing the geometric median is proposed in Sect. 3, and the local convergence of this scheme is proved in Sect. 6. Alternative approaches, based upon the Chambolle–Pock algorithm [8] and projected dual ascent [4], are compared to our approach.

Given a natural average or center of the measured data, one may then wish to determine the direction in which data points vary the most from the center. This direction is the most significant *principal component* of the data. Principal components of lesser significance are sought similarly but within the orthogonal complement of the more significant ones. Determining and analyzing such components is the subject of principal component analysis (PCA) [15]. The most common way of determining these components is to select them as the eigenvectors of the covariance matrix of the data. The more significant components correspond to the larger eigenvalues of the covariance matrix since each eigenvalue gives the variance of the data projected onto the corresponding eigenvector. As discussed in Sect. 4, determining each eigenvector can be formulated variationally in terms of finding a best fit line through the data, where the line minimizes the sum of squared distances to data points. See [5] and [10] for $\ell _1$-based alternatives to this criterion. Based upon examples presented in Sect. 4, this line is determined here more robustly by minimizing the sum of distances (not squared) to data points. In other words, analogous to defining an average as a geometric median *point*, a principal component is defined here as a geometric median *line*. In another context, the terms *robust PCA* have been associated with the separation of sparse and low-rank components in the given data. This separation was first proposed in [7], where a low-rank component is measured with the spectral norm and a sparse component with the $\ell _1$ norm. See also the related approaches of [23] and [18], where a low-rank component is separated from a column sparse component using similar norms. As in the works of [10] and [17], we do not assume here that the given data may be decomposed into sparse and low-rank components. Our task is instead to decompose (maximal rank) data into components which may all have significant energy in the data. The sum of distances (not squared) between data points and best lines is considered in [10] to obtain robust principal components all at once, and a convex relaxation of this approach is analyzed in [17]. In our approach, geometric median lines are obtained sequentially in decreasing orthogonal subspaces. In Sect. 4, an iterative scheme is proposed for computing these lines, and the scheme is based upon that used for computing the geometric median point. However, since the merit function is not convex, uniqueness of minimizers cannot be expected. Nevertheless, local convergence of the scheme to a minimizer is proved in Sect. 7. For approaches to PCA involving maximization of an $\ell _1$ norm we refer to [16] and [18].

Suppose that the data are rotated to an axis system aligned with principal components and that they are then scaled along each new axis to normalize the respective variances to unity. When this rotation and scaling is carried out by standard methods using $\ell _2$ measures, the transformed data have a covariance matrix equal to the identity. Then the data are said to have been *sphered*. In particular, the new data coordinates are statistically uncorrelated. However, they are not necessarily statistically independent [14]. (See, e.g., the example of Fig. 5 with $m=1$ in (5.1) so that the data are sphered but the coordinates do not satisfy the independence criterion (5.4).) It might then be postulated that the data can be represented in a rotated axis system with respect to which coordinates are statistically independent. Determining and analyzing such a system is the subject of independent component analysis (ICA). In case the postulate holds, coordinates of the sphered data represent weighted sums of statistically independent variables, and by the Central Limit Theorem [1] histograms of such coordinates tend to be bell shaped. In order to identify the postulated rotation, it is standard to minimize the *Gaussianity* of histograms of coordinates in the desired rotated system. The approach proposed by [14] is to determine this rotation by maximizing a merit function which is known to be minimized by data with a Gaussian distribution. It is also argued in [14] that one such merit function is more robust to data outliers than another when it does not grow as rapidly with respect to the data. Such candidate merit functions are considered in Sect. 5. The optimization method of [24] is robust against local extrema. Here we propose an approach for determining the desired rotation by first targeting independence directly instead of using the indirect measure of Gaussianity. The merit function proposed in Sect. 5 is motivated by the observation that while sphered axes tend to be aligned with data clusters, independent axes tend to *separate* clusters. See the examples presented in Sect. 5 for details. A fixed point iteration scheme based on the optimality condition is proposed in Sect. 5 for computing robust independent components, and the local convergence of this scheme is proved in Sect. 8.

The paper is outlined as follows. In Sect. 2, standard $\ell _2$ approaches to PCA and ICA are summarized, particularly to establish the background used later for the presentation of more robust methods. In Sect. 3, a robust method of data centering is proposed using the geometric median. In Sect. 4, a robust method for determining principal components is proposed using lines which are best fit in the sense that the sum of distances (not squared) to the data points is minimized within the subspace orthogonal to other components. In Sect. 5, a robust method for determining independent components is proposed which maximizes separations among sphered data clusters. Due to the nonlinearity of the respective optimality conditions, iterative schemes are proposed in Sects. 3–5 to solve the respective optimization problems. Local convergence of these schemes is proved in Sects. 6–8. In Sect. 9, the proposed methods are applied to a magnetic resonance image sequence to separate intensity changes due to physiological motion from those due to contrast agent, and benefits of the robust methods are demonstrated with respect to this realistic example. See also [20] and [22]. The paper ends with a summary in Sect. 10.

## Summary of $\ell _2$ Approaches to PCA and ICA

Let an unknown random vector $\mathrm{z} \in \mathbb {R}^m$ be given with components $\{\mathrm{z}_i\}_{i=1}^m$ which will be called *sources*. For example, the sources could be random variables associated with sounds produced independently at a *cocktail party*. The sources are assumed to satisfy the following:

1. For $1\le i\ne j\le m$, $\mathrm{z}_i$ and ${z}_j$ are statistically independent.
2. No ${z}_i$ is normally distributed.
3. For $1\le i\le m$, the variance $\sigma _i^2 = {E}[({z}_i-{E}[{z}_i])^2]$ of ${z}_i$ is positive.

Here, *E* denotes the expectation. Since the sources are statistically independent, they are uncorrelated [14]. Let their positive definite diagonal covariance matrix be denoted by

$$\begin{aligned} C({z}) = \{{E}[{z}_i-{E}[{z}_i], {z}_j-{E}[{z}_j]]\}_{i,j=1}^m = \text{ diag }\{\sigma _i^2\}_{i=1}^m \end{aligned}$$

which is unknown. Let a random vector ${y} \in \mathbb {R}^m$ be defined through a measurement process

2.1 $$\begin{aligned} {y} = A{z} \end{aligned}$$

modeled in terms of the *mixing matrix*$A \in \mathbb {R}^{m\times m}$. The components $\{{y}_i\}_{i=1}^m$ of *y* will be called measurements. For example, the measurements could be random variables associated with sounds recorded by separate microphones at the cocktail party mentioned above. In this case, it may be assumed naturally that each microphone records a weighted sum of sources, where a weight is stronger when the source is nearer, and the set of vectors of such weights for the respective microphones is linearly independent. Under the assumption that the mixing matrix is invertible, the goal is to determine a matrix $W\in \mathbb {R}^{m\times m}$ such that the components $\{{x}_i\}_{i=1}^m$ of the random vector

2.2 $$\begin{aligned} {x} = W{ y} \end{aligned}$$

estimate the sources in the following sense. First, normalizing ${z}=A^{-1}{y}$ according to $C({z})^{-\frac{1}{2}}{ z}$ removes the ambiguity of unknown variances by setting the covariance matrix to the identity. Secondly, since the order and sign of components in $C({z})^{-\frac{1}{2}}{z}$ are unknown, the alternative $PC({z})^{-\frac{1}{2}}{z}$ also satisfies the source assumptions when $P\in \mathbb {R}^{m\times m}$ is any matrix satisfying $(P_{q_i,j})^2=\delta _{i,j}$ with $\{q_i\}_{i=1}^m$ being a permutation of $\{i\}_{i=1}^m$. Thus, *W* estimates a product $PC({z})^{-\frac{1}{2}}A^{-1}$, and the covariance matrix of *x* in (2.2) is the identity.

Suppose that each random measurement variable ${y}_i$ is sampled directly to obtain *n* samples $\{y_{ij}\}_{j=1}^n$. Implicitly underlying these are samples $\{z_{ij}\}_{j=1}^n$ of each random source variable ${z}_i$. Define the sample vectors ${{\varvec{y}}}_i = \{y_{ij}\}_{j=1}^n$, ${{\varvec{z}}}_i = \{z_{ij}\}_{j=1}^n$, $i=1,\ldots ,m$. According to the linear model in (2.1), the matrices $Y=\{{{\varvec{y}}}_1,\ldots ,{{\varvec{y}}}_m\}^\mathrm{T} \in \mathbb {R}^{m\times n}$ and $Z=\{{{\varvec{z}}}_1,\ldots ,{{\varvec{z}}}_m\}^\mathrm{T} \in \mathbb {R}^{m\times n}$ are related by

2.3 $$\begin{aligned} Y = AZ. \end{aligned}$$

By (2.2), the estimation $X = \{{{\varvec{x}}}_1,\ldots ,{{\varvec{x}}}_m\}^\mathrm{T}\in \mathbb {R}^{m\times n}$ of the sources satisfies

2.4 $$\begin{aligned} X = WY. \end{aligned}$$

The matrix *W* is determined stepwise in terms of its singular value decomposition

2.5 $$\begin{aligned} W = U\Lambda ^{-\frac{1}{2}} V^\mathrm{T}, \end{aligned}$$

where $U,V \in \mathbb {R}^{m\times m}$ are orthogonal and $\Lambda \in \mathbb {R}^{m\times m}$ is positive definite and diagonal. Specifically, after the data are centered

2.6 $$\begin{aligned} Y_\mathrm{c} = Y - {\bar{Y}} \end{aligned}$$

with

2.7 $$\begin{aligned} {\bar{Y}} = \{\bar{{{\varvec{y}}}}_1,\ldots , \bar{{{\varvec{y}}}}_m\}^\mathrm{T}, \quad \bar{{{\varvec{y}}}}_i = \frac{1}{n}\sum _{j=1}^n y_{ij} \end{aligned}$$

the product $V^\mathrm{T}Y_\mathrm{c}$ should rotate the data so that the new coordinate axes are aligned with the visually natural axes of the cluster of data points $\{Y{\hat{{\varvec{e}}}}_j\}_{j=1}^n$, ${\hat{{\varvec{e}}}}_j \in \mathbb {R}^n$, $({\hat{{\varvec{e}}}}_j)_i = \delta _{i,j}$. After this rotation, the product

2.8 $$\begin{aligned} Y_\mathrm{s} = \Lambda ^{-\frac{1}{2}}V^\mathrm{T}Y_\mathrm{c} \end{aligned}$$

should scale the data so that the variance along each new coordinate axis is unity. For this reason, the data $Y_\mathrm{s}$ are said to be *sphered*. The final orthogonal matrix *U* in (2.5) is chosen so that the components of the random variable *x* in (2.2) are maximally independent in a sense made precise below.

To determine the transformations *V* and $\Lambda$, the covariance matrix of the sphered data is required to be the identity,

2.9 $$\begin{aligned} I = {\textstyle \frac{1}{n}} Y_\mathrm{s}Y_\mathrm{s}^\mathrm{T} = \Lambda ^{-\frac{1}{2}}V^\mathrm{T}[{\textstyle \frac{1}{n}}Y_\mathrm{c}Y_\mathrm{c}^\mathrm{T}] V\Lambda ^{-\frac{1}{2}}, \end{aligned}$$

which is accomplished by determining the matrices *V* and $\Lambda$ from the eigenspace decomposition of the centered data,

2.10 $$\begin{aligned} {\textstyle \frac{1}{n}}Y_\mathrm{c}Y_\mathrm{c}^\mathrm{T} = V\Lambda V^\mathrm{T}, \quad V^\mathrm{T}V = I. \end{aligned}$$

The columns of *V* are the so-called *principal components* of the data *Y*. Analyzing this decomposition is the subject of *principal component analysis* (PCA). For instance, the sampled data may be filtered by projecting these data onto subspaces spanned by principal components. For this, assume that the entries of $\Lambda = \text{ diag }\{\lambda _i\}_{i=1}^m$ and $V=\{{{\varvec{v}}}_1,\ldots ,{{\varvec{v}}}_m\}$ are ordered according to $\lambda _1 \ge \lambda _2 \ge \cdots \ge \lambda _m$. This means that the variance $\lambda _i = \frac{1}{n}\Vert Y_\mathrm{c}^\mathrm{T}{{\varvec{v}}}_i\Vert _{\ell _2}^2$ of the data $Y_\mathrm{c}$ along the axis ${{\varvec{v}}}_i$ is larger than the variance $\lambda _j$ along the axis ${{\varvec{v}}}_j$ for $i<j$. To select only the $r<m$ components with respect to which the data have the most variation, define the projected data $Y_P \approx Y$ by

2.11 $$\begin{aligned} Y_P = {\bar{Y}} + V\Lambda ^{\frac{1}{2}}P^\mathrm{T}P\Lambda ^{-\frac{1}{2}}V^\mathrm{T}(Y-{\bar{Y}}), \end{aligned}$$

where the projector $P \in \mathbb {R}^{r\times m}$ is defined with entries $P_{i,j} = \delta _{i,j}$. Note that with (2.6), (2.8), and (2.10), this result can be rewritten as $Y_P = {\bar{Y}} + {\textstyle \frac{1}{n}}Y_\mathrm{c}(PY_\mathrm{s})^\mathrm{T}(PY_\mathrm{s})$.

Next, the transformation *U* in (2.1) is determined so that the components of the random variable *x* in (2.2) are independent. While the rows of the sphered data $Y_\mathrm{s}$ are statistically uncorrelated, they are not necessarily statistically independent [14]. A criterion is now sought for a final rotation of axes which gives the desired independence. Since as seen in (2.1) measurements are sums of independent random variables, the Central Limit Theorem suggests why the measurements tend to be normally distributed [1]. The matrix *U* is often chosen to reverse this effect, i.e., to make the components of *x* depart from being normally distributed as much as possible. Here, the significance of the assumption that no component $z_i$ be normally distributed can be seen, as otherwise the proposed measure of independence would not bring a separation of sources in the following. For the required statistical constructions, let *E*[*x*] denote the expectation of a random variable *x*. Since a normally distributed random variable *n* with mean 0 and variance $\sigma ^2$ has moments

2.12 $$\begin{aligned}&{E}[|{n}|^m] = \kappa _m \sigma ^m, \nonumber \\&\kappa _m = \left\{ \begin{array}{rl} (m-1)\cdot (m-3)\cdot (m-5)\cdots 3\cdots 1, &{} m\, \mathrm { even} \\ \sqrt{\frac{2}{\pi }} (m-1)\cdot (m-3)\cdot (m-5)\cdots 2, &{} m\, \mathrm { odd}, \\ \end{array} \right. \nonumber \\ \end{aligned}$$

it follows that the Kurtosis $K = K_4$,

2.13 $$\begin{aligned} K_m(x) = E[|x- E[x]|^m]-\kappa _m { E}[|{x}-{E}[{x}]|^2]^{m/2}, \end{aligned}$$

of *n* satisfies $K({n}) = {E}[|{n}|^4]-3{E}[|{n}|^2]^2 = 0$. Hence, a parameter-dependent random variable may be made to depart maximally from being normally distributed by maximizing the square of its Kurtosis with respect to parameters. Applying this criterion to the rows of

2.14 $$\begin{aligned} X_{c} = UY_{s}, \end{aligned}$$

the rows of $U = \{{{\varvec{u}}}_i^\mathrm{T}\}_{i=1}^m$ are determined as follows. Define

2.15 $$\begin{aligned} U_l= & {} \{{{\varvec{u}}}_1,\ldots ,{{\varvec{u}}}_l\}^\mathrm{T}, \quad U_l \in \mathbb {R}^{l\times m}, \nonumber \\&l=1,\ldots ,m, \quad U_0 = \{\} \end{aligned}$$

and the projected data

2.16 $$\begin{aligned} Y_l = (I-U_{l-1}^\mathrm{T}U_{l-1})Y_\mathrm{s},\quad l=2,\ldots ,m, \quad Y_1 = Y_\mathrm{s}\nonumber \\ \end{aligned}$$

whose columns lie in $T_l \subset \mathbb {R}^m$ defined as the range of $Y_l$. Note that

2.17 $$\begin{aligned} {{\varvec{u}}}_l^\mathrm{T}Y_l = {{\varvec{u}}}_l^\mathrm{T}Y_\mathrm{s}- {{\varvec{u}}}_l^\mathrm{T}U_{l-1}^\mathrm{T}U_{l-1}Y_\mathrm{s} = {{\varvec{u}}}_l^\mathrm{T}Y_\mathrm{s}, \quad {{\varvec{u}}} \in T_l. \end{aligned}$$

Given $T_l$, let the *l*th column of $U^\mathrm{T}$ be determined inductively by

2.18 $$\begin{aligned} {{\varvec{u}}}_l = \frac{\mathbf{u}_l}{\Vert \mathbf{u}_l\Vert _{\ell _2}}, \quad \mathbf{u}_l = {\mathop {\hbox {argmax}}\limits _{\mathbf{u}\in T_l}}\, F\left( \frac{\mathbf{u}^\mathrm{T}Y_l}{\Vert \mathbf{u}\Vert _{\ell _2}}\right) , \quad F({{\varvec{x}}}) = K^2({{\varvec{x}}}).\nonumber \\ \end{aligned}$$

By (2.9) and (2.17), the second moment of $\mathbf{u}^\mathrm{T}Y_l/\Vert \mathbf{u}\Vert _{\ell _2}$ is

2.19 $$\begin{aligned}&\mathbf{u}^\mathrm{T}[Y_lY_l^\mathrm{T}/n]\mathbf{u}/\Vert \mathbf{u}\Vert _{\ell _2}^2 = \mathbf{u}^\mathrm{T}[Y_\mathrm{s}Y_\mathrm{s}^\mathrm{T}/n]\mathbf{u}/\Vert \mathbf{u}\Vert _{\ell _2}^2\nonumber \\&\quad = \mathbf{u}^\mathrm{T}{} \mathbf{u}/\Vert \mathbf{u}\Vert _{\ell _2}^2 = 1, \quad \mathbf{u} \in T_l. \end{aligned}$$

Thus, *F* in (2.18) is computed according to

2.20 $$\begin{aligned} F({{\varvec{x}}}) = \left[ \frac{1}{n}\Vert {{\varvec{x}}}\Vert _{\ell _4}^4 -3 \right] ^2 \quad \text{ for } \quad \frac{1}{n}\Vert {{\varvec{x}}}\Vert _{\ell _2}^2=1. \end{aligned}$$

In this way, the rows of *U* are determined sequentially so that the earlier components of *x* depart from being normally distributed more than later components. Alternatively, all components of *x* may be estimated with roughly equal quality by determining all rows of *U* simultaneously through maximizing $\sum _{l=1}^m F({{\varvec{u}}}_l^\mathrm{T} Y_\mathrm{s})$ under the conditions ${{\varvec{u}}}_i^\mathrm{T}{{\varvec{u}}}_j = \delta _{i,j}$, $1\le i,j\le m$. Once matrices *U*, $\Lambda$, and *V* are determined, the source samples are estimated according to (2.4) and (2.5).

The columns of $$V\Lambda ^{\frac{1}{2}}U^\mathrm{T}$$ are the so-called *independent components* of the data *Y*. Analyzing this decomposition is the subject of *independent component analysis* (ICA). For instance, the sampled data may be filtered by projecting these data onto subspaces spanned by independent components. Specifically, to select the $$r<m$$ desired independent components $$\{q_1,\ldots ,q_r\}$$$$\subset $$$$\{1,\ldots ,m\}$$, define the projected data $$Y_Q \approx Y$$ by

2.21 $$\begin{aligned} Y_Q = {\bar{Y}} + V\Lambda ^{\frac{1}{2}}U^\mathrm{T} Q^\mathrm{T}QU\Lambda ^{-\frac{1}{2}}V^\mathrm{T}(Y-{\bar{Y}}), \end{aligned}$$

where the projector $Q \in \mathbb {R}^{r\times m}$ is defined with entries $Q_{ij} = \delta _{q_i,j}$. Note that with (2.6), (2.8), (2.10) and (2.14), this result can be rewritten as $Y_Q = {\bar{Y}} + {\textstyle \frac{1}{n}}Y_\mathrm{c}(QX_\mathrm{c})^\mathrm{T}(QX_\mathrm{c})$.

In the calculations above, it is implicitly assumed that the number of samples *n* is at least as large as the number of sources *m*. Otherwise, the rank *n* of the covariance matrix $\frac{1}{n} Y_\mathrm{s} Y_\mathrm{s}^\mathrm{T}$ would be less than its dimension *m*, and the diagonal matrix $\Lambda$ in (2.10) would not be positive definite. In case $n<m$ does in fact hold, because so few samples have been collected, one might be inclined simply to replace *Y* with $Y^\mathrm{T}$ and therefore reverse the roles of time and space in the data. However, the data must possess an ergodicity property for the results with transposed data to be roughly equivalent to those without transposed data. Since such a property may not generally hold, the matrices above are determined here as follows; see also [6]. With ${\bar{Y}}$ and $Y_\mathrm{c}$ given by (2.6), define the singular value decomposition $Y_\mathrm{c}/\sqrt{n} = V{\hat{\Sigma }}{\hat{Y}}_\mathrm{s}/\sqrt{n}$ in terms of rotation matrices $V\in \mathbb {R}^{m\times m}$ and ${\hat{Y}}_\mathrm{s}/\sqrt{n}\in \mathbb {R}^{n\times n}$ and a rectangular matrix ${\hat{\Sigma }} \in \mathbb {R}^{m\times n}$ for which ${\hat{\Lambda }} = {\hat{\Sigma }}^\mathrm{T}{\hat{\Sigma }} \in \mathbb {R}^{n\times n}$ is diagonal and positive definite. The matrices ${\hat{\Lambda }}$ and ${\hat{Y}}_\mathrm{s}$ are determined from the eigenspace decomposition $Y_\mathrm{c}^\mathrm{T}Y_\mathrm{c} = {\hat{Y}}_\mathrm{s}^\mathrm{T} {\hat{\Lambda }} {\hat{Y}}_\mathrm{s}$, ${\hat{Y}}_\mathrm{s} {\hat{Y}}_\mathrm{s}^\mathrm{T} = nI$. Since the last $m-n$ rows of ${\hat{\Sigma }}$ are zero, the last $m-n$ columns $\tilde{V}\in \mathbb {R}^{m\times (m-n)}$ of $V=[{\hat{V}},\tilde{V}]$ may be neglected to obtain $Y_\mathrm{c}= {\hat{V}}{\hat{\Lambda }}^{\frac{1}{2}}{\hat{Y}}_\mathrm{s}$. The matrix ${\hat{V}} \in \mathbb {R}^{m\times n}$ is determined from ${\hat{V}} = Y_\mathrm{c}{\hat{Y}}_\mathrm{s}^\mathrm{T}{\hat{\Lambda }}^{-\frac{1}{2}}/n$. The sphered data ${\hat{Y}}_\mathrm{s}$ are transformed by the rotation matrix ${\hat{U}} \in \mathbb {R}^{n\times n}$ maximizing independence of the rows of ${\hat{X}}_\mathrm{c} = {\hat{U}}{\hat{Y}}_\mathrm{s} \in \mathbb {R}^{n\times n}$. Note that with $\Lambda = {\hat{\Sigma }}{\hat{\Sigma }}^\mathrm{T}$ it also follows that $Y_\mathrm{c}Y_\mathrm{c}^\mathrm{T} = V{\hat{\Sigma }}[{\hat{Y}}_\mathrm{s}{\hat{Y}}_\mathrm{s}^\mathrm{T}] {\hat{\Sigma }}^\mathrm{T}V^\mathrm{T} = nV\Lambda V^\mathrm{T}$ holds, giving (2.10). Let ${\hat{Y}}_\mathrm{s}$ be padded with $m-n$ zero rows to obtain $Y_\mathrm{s} = [{\hat{Y}}_\mathrm{s};0] \in \mathbb {R}^{m\times n}$. With $\Sigma = \Lambda ^{\frac{1}{2}}$, it follows from the singular value decomposition of $Y_\mathrm{c}$ that $Y_\mathrm{c} = V\Sigma Y_\mathrm{s}$ holds, and hence the counterpart $Y_\mathrm{s} = (\Lambda ^\dagger )^{\frac{1}{2}} V^\mathrm{T}Y_\mathrm{c}$ to (2.8) holds, where $\Lambda ^\dagger$ denotes the pseudo-inverse of $\Lambda$. The rotation matrix $U = [[{\hat{U}},0];[0,\tilde{U}]] \in \mathbb {R}^{m\times m}$ can be defined by supplementing ${\hat{U}}$ with the rotation matrix $\tilde{U}\in \mathbb {R}^{(m-n)\times (m-n)}$ and otherwise padding with zeros, and $X_\mathrm{c} = UY_\mathrm{s} \in \mathbb {R}^{m\times n}$ can be defined to give (2.14). However, according to $X_\mathrm{c} = UY_\mathrm{s} = [[{\hat{U}},0];[0,\tilde{U}]][{\hat{Y}}_\mathrm{s};0] = [{\hat{X}}_\mathrm{c};0]$, the last $m-n$ rows of $X_\mathrm{c}$ are zero, contrary to the objective that the rows be independent. Thus, ${\hat{X}}_\mathrm{c}$ marks the end of the calculation and ${\hat{X}} = {\hat{U}}{\hat{\Lambda }}^{-\frac{1}{2}}{\hat{V}}^\mathrm{T}Y$ gives the maximum number of independent components which can be determined from the undersampled data. Finally, for projectors $P,Q \in \mathbb {R}^{n\times n}$, (2.11) and (2.21) become $Y_P = {\bar{Y}} + \frac{1}{n}Y_\mathrm{c}(P{\hat{Y}}_\mathrm{s})^\mathrm{T} (P{\hat{Y}}_\mathrm{s})$ and $Y_Q = {\bar{Y}} + \frac{1}{n}Y_\mathrm{c} (Q{\hat{X}}_\mathrm{s})^\mathrm{T}(Q{\hat{X}}_\mathrm{s})$, respectively.

## $\ell _1$ Approach to Centering

That $\ell _1$ measures lead to statistically robust results may be highlighted by the following simple example. Suppose samples $Y = \{y_j\}_{j=1}^n = \{0,1,\ldots ,1\}\in \mathbb {R}^n$, $n>2$, have been collected, where the first measurement is clearly an outlier. The $\ell _2$ mean of these data is given by

3.1 $$\begin{aligned} {\mathop {\hbox {argmin}}\limits _{\mu \in {\mathbb {R}}}}\, \sum _{j=1}^n |y_j-\mu |^2 = \text{ mean }({{\varvec{y}}}) = (n-1)/n \end{aligned}$$

which is clearly influenced by the outlier. On the other hand, the $\ell _1$ mean is given by

3.2 $$\begin{aligned} {\mathop {\hbox {argmin}}\limits _{\mu \in \mathbb {R}}}\, \sum _{j=1}^n |y_j-\mu | = \text{ median }({{\varvec{y}}}) = 1 \end{aligned}$$

which is insensitive to the outlier. Here, the median is defined as the middle entry in the sorted list of values, if *n* is odd, or else the average of two middle values, if *n* is even.

For a generalization of this robust scalar mean to its counterpart for vectors, let the data $Y = \{{{\varvec{y}}}_1,\ldots ,{{\varvec{y}}}_m\}^\mathrm{T} \in \mathbb {R}^{m\times n}$, ${{\varvec{y}}}_i = \{y_{ij}\}_{j=1}^n$, with columns $Y{\hat{{\varvec{e}}}}_j$, ${\hat{{\varvec{e}}}}_j \in \mathbb {R}^n$, $({\hat{{\varvec{e}}}}_j)_i = \delta _{i,j}$, be given as in Sect. 2. Note that if the $\ell _1$ mean were defined according to

**Fig. 1:**
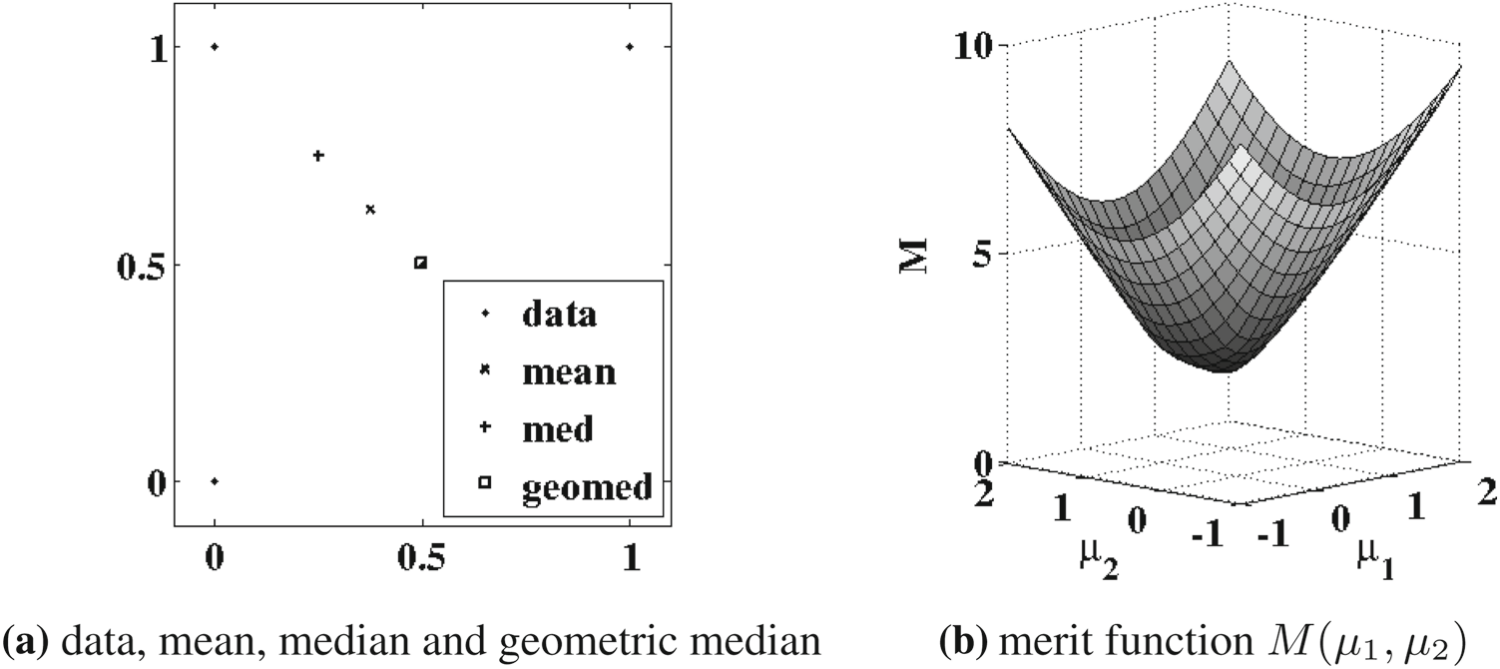
The mean, median, and geometric median are compared in **a**, where the data of (3.5) are shown with $\cdot$, the mean with $\times$, the median with $+$, and the geometric median with $\Box$. Shown in **b** is the landscape of the merit function *M* in (3.4)

3.3 $$\begin{aligned}&{\mathop {\hbox {argmin}}\limits _{{{\varvec{\mu }}}\in \mathbb {R}^m}}\, \sum _{j=1}^n \Vert Y{\hat{{\varvec{e}}}}_j-{{\varvec{\mu }}}\Vert _{\ell _1}\nonumber \\&=\left\{ {\mathop {\hbox {argmin}}\limits _{\mu _i\in \mathbb {R}}}\, \sum _{j=1}^n |y_{ij}-\mu _i| \right\} _{i=1}^m = \left\{ \text{ median }\{y_{ij}\}_{j=1}^n \right\} _{i=1}^m,\nonumber \\ \end{aligned}$$

then the solution would be unnaturally determined componentwise through decoupled minimizations. By contrast, the following $\ell _1$ mean for vectors [11], i.e., the geometric median [19],

3.4 $$\begin{aligned} {\bar{Y}} = {\mathop {\hbox {argmin}}\limits _{{{\varvec{\mu }}}\in \mathbb {R}^m}}\, M({{\varvec{\mu }}}), \quad M({{\varvec{\mu }}}) = \sum _{j=1}^n \Vert Y{\hat{{\varvec{e}}}}_j-{{\varvec{\mu }}}\Vert _{\ell _2} \end{aligned}$$

minimizes, in a natural way, the $\ell _1$ norm of Euclidean distances between the data points and the selected mean. The robustness of this measure in relation to the mean or median can be highlighted by the following simple example, which is illustrated in Fig. 1a. Here the data are given by

3.5 $$\begin{aligned} Y = \left[ \begin{array}{cccc} 0 &{} \frac{1}{2} &{} 1 &{} 0 \\ 0 &{} \frac{1}{2} &{} 1 &{} 1 \end{array} \right] \end{aligned}$$

marked with $\cdot$ in Fig. 1a, and the point (0, 1) may be regarded as an outlier from points otherwise lying on the line between (0, 0) and (1, 1). The componentwise mean of the data gives (0.375, 0.625) marked with $\times$ in Fig. 1a. Then the componentwise median gives (0.25, 0.75) as marked with $+$ in Fig. 1a. Finally, the measure defined in (3.4) gives the geometric median ${\bar{Y}} = (0.4996,0.5004)$ marked with $\Box$ in Fig. 1a, where the smooth landscape for the merit function *M* is shown in Fig. 1b. Because of the natural result obtained by the geometric median in Fig. 1a, (3.4) will be used here for the $\ell _1$ vector mean.

To compute the $\ell _1$ mean of (3.4), the following iteration is used. For $\tau >0$, compute iteratively $D_l\in \mathbb {R}^{m\times n}$ and ${{\varvec{\mu }}}_l\in \mathbb {R}^m$ by

3.6 $$\begin{aligned}&D_{l+1}{\hat{{\varvec{e}}}}_j = \frac{D_l{\hat{{\varvec{e}}}}_j+\tau ({{\varvec{\mu }}}_l-Y{\hat{{\varvec{e}}}}_j)}{1+ \tau \Vert {{\varvec{\mu }}}_l-Y{\hat{{\varvec{e}}}}_j\Vert _{\ell _2}}, \quad j=1,\ldots ,n \end{aligned}$$

3.7 $$\begin{aligned}&{{\varvec{\mu }}}_{l+1} = \sum _{j=1}^n \frac{(D_l-\tau Y){\hat{{\varvec{e}}}}_j}{1+ \tau \Vert {{\varvec{\mu }}}_l-Y{\hat{{\varvec{e}}}}_j\Vert _{\ell _2}} \Big / \sum _{j=1}^n \frac{-\tau }{1+ \tau \Vert {{\varvec{\mu }}}_l-Y{\hat{{\varvec{e}}}}_j\Vert _{\ell _2}}.\nonumber \\ \end{aligned}$$

The motivation for this iteration is based upon the optimality conditions (6.9) derived later in Sect. 6. A continuum level steepest descent approach to solving (6.9) is given formally by

3.8 $$\begin{aligned}&D^\prime (t){\hat{{\varvec{e}}}}_j = ({{\varvec{\mu }}}(t)-Y{\hat{{\varvec{e}}}}_j)\nonumber \\&\quad - \Vert {{\varvec{\mu }}}(t)-Y{\hat{{\varvec{e}}}}_j\Vert _{\ell _2}D(t){\hat{{\varvec{e}}}}_j, \quad D:[0,\infty )\rightarrow \mathbb {R}^{m\times n} \end{aligned}$$

3.9 $$\begin{aligned}&\sum _{j=1}^n \left[ ({{\varvec{\mu }}}(t)-Y{\hat{{\varvec{e}}}}_j)\right. \nonumber \\&\left. - \Vert {{\varvec{\mu }}}(t)-Y{\hat{{\varvec{e}}}}_j\Vert _{\ell _2}D(t){\hat{{\varvec{e}}}}_j \right] =0, \quad {{\varvec{\mu }}}:[0,\infty )\rightarrow \mathbb {R}^m \end{aligned}$$

where (3.9) implicitly defines ${{\varvec{\mu }}}(t)$ so that the sum over *j* in (3.8) and hence $\sum _{j=1}^n D(t){\hat{{\varvec{e}}}}_j$ remains 0. A semi-implicit time-stepping approach to solving (3.8)–(3.9) is given by

3.10 $$\begin{aligned}&\tau ^{-1}(D_{l+1}-D_l){\hat{{\varvec{e}}}}_j = ({{\varvec{\mu }}}_l-Y{\hat{{\varvec{e}}}}_j)- \Vert {{\varvec{\mu }}}_l-Y{\hat{{\varvec{e}}}}_j\Vert _{\ell _2}D_{l+1}{\hat{{\varvec{e}}}}_j \nonumber \\\end{aligned}$$

3.11 $$\begin{aligned}&\sum _{j=1}^n \frac{D_l{\hat{{\varvec{e}}}}_j+\tau ({{\varvec{\mu }}}_{l+1}-Y{\hat{{\varvec{e}}}}_j)}{1+\tau \Vert {{\varvec{\mu }}}_{l}-Y{\hat{{\varvec{e}}}}_j\Vert _{\ell _2}}=0 \end{aligned}$$

where (3.11) defines ${{\varvec{\mu }}}_{l+1}$ to correct the departure of $\sum _{j=1}^n D_{l+1}{\hat{{\varvec{e}}}}_j$ in (3.10) from 0, as required by the last equation in (6.9). After rearranging terms, the scheme (3.6)–(3.7) is seen to be equivalent to (3.10)–(3.11). This derivation of the scheme (3.6)–(3.7) offers a heuristic explanation for the advantage of choosing $\tau$ large so that the desired steady state of (3.8)–(3.9) is achieved rapidly. Further details of this iteration and its convergence analysis are given in Sect. 6. The $\ell _1$-mean is given by taking the limit,

3.12 $$\begin{aligned} {\bar{Y}} = \lim _{l\rightarrow \infty } {{\varvec{\mu }}}_l. \end{aligned}$$

After these calculations have been completed, the centered data are given by $Y_\mathrm{c} = Y-{\bar{Y}}$, the counterpart to (2.6) with (3.4) replacing (2.7).

**Fig. 2:**
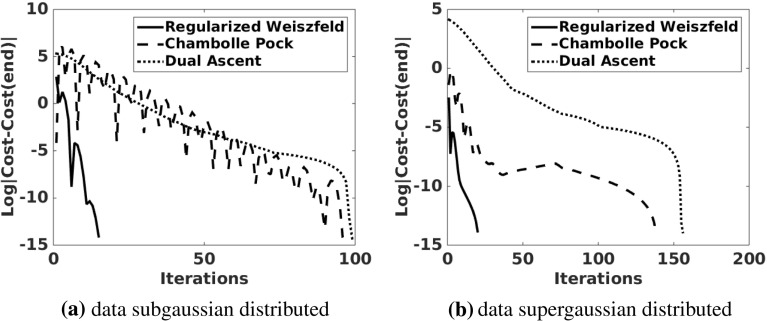
Convergence history for the regularized Weiszfeld scheme (3.6)–(3.7), the Chambolle–Pock scheme (3.13), and the dual ascent scheme (3.14) to compute the geometric median for data which are **a** subgaussian (uniformly) distributed and **b** supergaussian (Laplacian) distributed. (The authors wish to thank the referee who provided the code used for this comparison.)

**Fig. 3:**
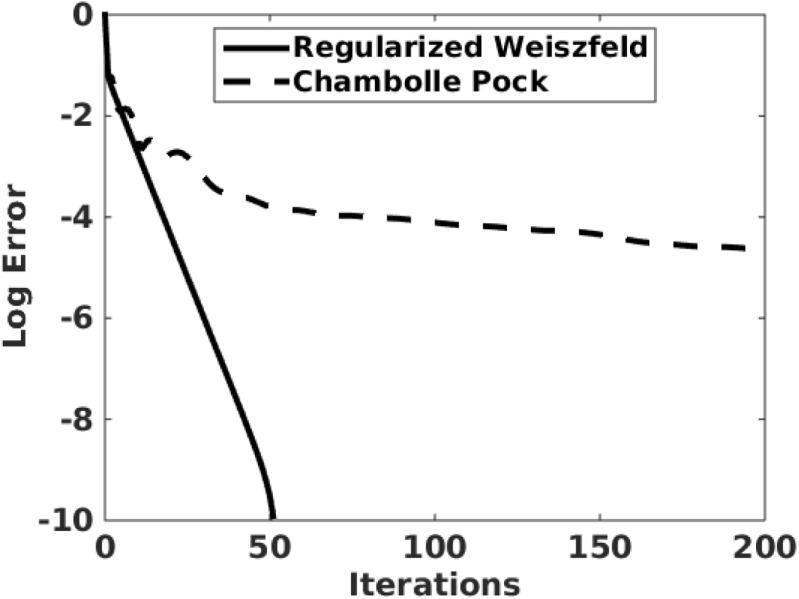
Convergence history for the computation of ${\bar{Y}}$ for the DCE-MRI data of Sect. 9 according to the regularized Weiszfeld scheme (3.6)–(3.7) and the Chambolle–Pock scheme (3.13)

Note that the iteration (3.6)–(3.7) agrees with the Weiszfeld Algorithm for $\tau \rightarrow \infty$ [21]. Since this limit may lead to undefined terms, the proposed scheme may be regarded as a regularized Weiszfeld iteration. This scheme is compared in Figs. 2 and 3 with two alternatives which are described next.

The first alternative approach is given by the Chambolle–Pock Algorithm [8], which can be written for ${{\varvec{\mu }}}_l,{{\varvec{\nu }}}_l \in \mathbb {R}^m$, $D_l \in \mathbb {R}^{m\times n}$ as

3.13 $$\begin{aligned} \left\{ \begin{array}{rcl} D_{l+1}{\hat{{\varvec{e}}}}_j &{} = &{} P_B(D_l{\hat{{\varvec{e}}}}_j+\varsigma ({{\varvec{\nu }}}_l-Y{\hat{{\varvec{e}}}}_j)), \quad 1\le j\le n \\ {{\varvec{\mu }}}_{l+1} &{} = &{} {{\varvec{\mu }}}_l - \tau /n \sum _{j=1}^n D_{l+1}{\hat{{\varvec{e}}}}_j \\ {{\varvec{\nu }}}_{l+1} &{} = &{} {{\varvec{\mu }}}_{l+1} + \theta ({{\varvec{\mu }}}_{l+1}-{{\varvec{\mu }}}_l). \end{array} \right. \end{aligned}$$

In (3.13), $P_B$ is a projection onto the set $B=\{{{\varvec{v}}}\in \mathbb {R}^m: \Vert {{\varvec{\nu }}}\Vert _2 \le 1\}$ and the theoretical requirements are that $\theta =1$ and $0<\varsigma ,\tau < 1$ hold. Note that, as (3.5)–(3.6) can be seen as a semi-implicit time-stepping scheme for solving (3.8)–(3.9), (3.13) can be seen as an alternative explicit time-stepping scheme.

The second alternative approach is given by solving the dual problem $\sup _{D\in A\cap C} \sum _{j=1}^n ({\hat{{\varvec{e}}}}_jD)^\top Y{\hat{{\varvec{e}}}}_j$, where $A=\{D\in \mathbb {R}^{m\times n}: \Vert D{\hat{{\varvec{e}}}}_j\Vert _{\ell _2}\le 1,1\le j\le n\}$ und $C = \{D\in \mathbb {R}^{m\times n}: \sum _{j=1}^n D{\hat{{\varvec{e}}}}_j=0\}$. The solution $D^*$ is computed by a dual ascent iteration

3.14 $$\begin{aligned} D_{l+1} = D_{l} + \tau P_{A\cap C}Y, \end{aligned}$$

where $\tau >0$ is a stepsize and $P_{A\cap C}$ is a projection onto $A\cap C$, computed according to [4]. This ascent method can also be viewed as an explicit time-stepping scheme for solving the dual problem. After $D^*$ is obtained, the fixed point iteration ${{\varvec{\mu }}}_{l+1} = (1/n)\sum _{j=1}^n[Y{\hat{{\varvec{e}}}}_j-\Vert {{\varvec{\mu }}}_l-Y{\hat{{\varvec{e}}}}_j\Vert _{\ell _2} D^*{\hat{{\varvec{e}}}}_j]$ is used to compute a solution ${{\varvec{\mu }}}^*$ to the optimality condition in (6.9).

The convergence of the three schemes, regularized Weiszfeld (3.6)–(3.7), Chambolle–Pock (3.13), and dual ascent (3.14), is compared in Fig. 2. All parameters have been chosen manually to minimize the number of iterations required for convergence. For these comparisons, the data $Y\in \mathbb {R}^{m\times n}$, $m=2$, $n=1000$, are chosen so that the points $\{Y{\hat{{\varvec{e}}}}_j\}_{j=1}^n$ are subgaussian (uniformly, Y = rand(m,n)) distributed in Fig. 2a and supergaussian (Laplacian, Y = log(rand(m,n)./rand(m,n))) distributed in Fig. 2b. Clearly, the regularized Weiszfeld iteration converges much more rapidly. Since additional work is required to determine ${{\varvec{\mu }}}^*$ from the result of the dual ascent method, only the regularized Weiszfeld and the Chambolle–Pock iterations are compared in Fig. 3 for the realistic computation of ${\bar{Y}}$ for the example of Sect. 9. Again, the regularized Weiszfeld iteration converges much more rapidly. This convergence performance combined with the popularity of the Weiszfeld Algorithm has motivated the focus on the scheme (3.6)–(3.7) and its analysis for this work.

## $\ell _1$ Approach to PCA

To present our approach, we start with some preliminaries. Unless otherwise specified, it is assumed that $m\le n$. With *V* and $\Lambda$ in (2.10) given in terms of components as $V = \{{\hat{{{\varvec{v}}}}}_i\}_{i=1}^m$ and $\Lambda = \text{ diag }\{\lambda _i\}_{i=1}^m$, respectively, define

4.1 $$\begin{aligned} V_k = \{{\hat{{\varvec{v}}}}_1,\ldots ,{\hat{{\varvec{v}}}}_k\} \in \mathbb {R}^{m \times k} \end{aligned}$$

and the projected data

4.2 $$\begin{aligned} Y_k = (I-V_{k-1}V_{k-1}^\mathrm{T})Y_\mathrm{c},\quad k=2,\ldots ,m, \quad Y_1 = Y_\mathrm{c}.\nonumber \\ \end{aligned}$$

Let $S_k = {\mathcal R}(Y_k)$ where ${\mathcal R}$ denotes the range. For convenience, it is assumed here that the data $Y_\mathrm{c}$ have maximal rank so that $S_1 = \mathbb {R}^m$. Then ${{\varvec{0}}} = {{\varvec{v}}}^\mathrm{T}(I-V_{k-1}V_{k-1}^\mathrm{T})Y_\mathrm{c} = {{\varvec{v}}}^\mathrm{T}Y_k$ is equivalent to ${{\varvec{v}}} = V_{k-1}V_{k-1}^\mathrm{T}{{\varvec{v}}}$, which is equivalent to ${{\varvec{v}}} = {{\varvec{0}}}$ exactly when $V_{k-1}^\mathrm{T}{{\varvec{v}}} = {{\varvec{0}}}$. Hence,

4.3 $$\begin{aligned} S_k = {\mathcal R}(V_{k-1})^\perp , \quad k=2,\ldots ,m, \quad S_1 = \mathbb {R}^m. \end{aligned}$$

**Fig. 4:**
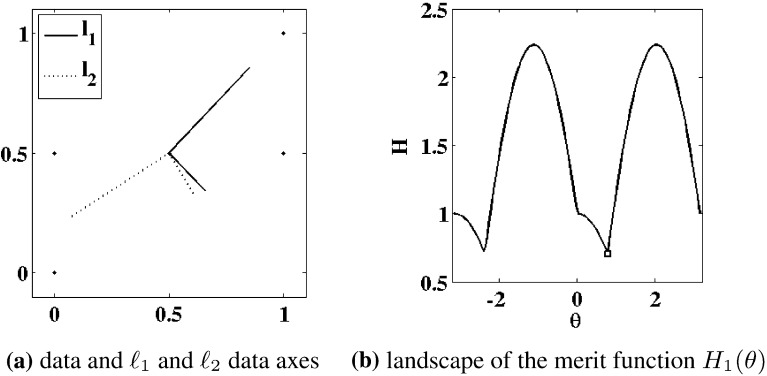
The $\ell _1$ (*solid*) and $\ell _2$ (*dotted*) data axes are compared in **a**, where the data of (4.9) are shown with $\cdot$. Shown in **b** is the landscape of the merit function $H_1(\theta )= H_1({\hat{{{\varvec{v}}}}}(\theta ))$ in (4.8) with ${\hat{{{\varvec{v}}}}}(\theta ) = {(}\cos (\theta ),\sin (\theta )$, which is minimized at $\Box$

Before presenting the proposed robust measure for determining visually natural data axes, a motivation is given by reformulating the $\ell _2$ eigenspace decomposition in (2.10) in terms of a least squares fit of an axis system to the cloud of data points. Given $S_k$, let the *k*th column of *V* and the *k*th diagonal entry of $\Lambda$ be determined inductively by the regression

4.4 $$\begin{aligned} {\hat{{{\varvec{v}}}}}_k= & {} \frac{{{\varvec{v}}}_k}{\Vert {{\varvec{v}}}_k\Vert _{\ell _2}}, \quad {{\varvec{v}}}_k = {\mathop {\hbox {argmin}}\limits _{{{\varvec{v}}}\in S_k}}\, {\tilde{H}}_k({{\varvec{v}}}), \nonumber \\ \lambda _k= & {} \Vert {\hat{{{\varvec{v}}}}}_k^\mathrm{T}Y_k/\sqrt{n}\Vert _{\ell _2}^2, \quad k=1,\ldots ,m, \end{aligned}$$

where

4.5 $$\begin{aligned} {\tilde{H}}_{k}({{\varvec{v}}})= & {} \sum _{j=1}^n \Big \Vert \left( \frac{{{\varvec{v}}}{{\varvec{v}}}^\mathrm{T}}{\Vert {{\varvec{v}}}\Vert _{\ell _2}^2}-I \right) Y_k{\hat{{\varvec{e}}}}_j \Big \Vert _{\ell _2}^2, \nonumber \\&{{\varvec{v}}}\ne {{\varvec{0}}}, \quad {\tilde{H}}_k({{\varvec{0}}}) = \Vert Y_k\Vert _\mathrm{F}^2. \end{aligned}$$

Since minimizing

4.6 $$\begin{aligned} {\tilde{H}}_k({\hat{{{\varvec{v}}}}})= & {} \displaystyle \sum _{j=1}^n {\hat{{\varvec{e}}}}_j^\mathrm{T}Y_k^\mathrm{T}({\hat{{{\varvec{v}}}}}{\hat{{{\varvec{v}}}}}^\mathrm{T}-I)^\mathrm{T}({\hat{{{\varvec{v}}}}}{\hat{{{\varvec{v}}}}}^\mathrm{T}-I)Y_k{\hat{{\varvec{e}}}}_j\nonumber \\= & {} \sum _{j=1}^n {\hat{{\varvec{e}}}}_j^\mathrm{T}Y_k^\mathrm{T}(I-{\hat{{{\varvec{v}}}}}{\hat{{{\varvec{v}}}}}^\mathrm{T})Y_k{\hat{{\varvec{e}}}}_j\nonumber \\= & {} {\displaystyle \sum _{j=1}^n \left[ |Y_k{\hat{{\varvec{e}}}}_j|^2- |{\hat{{\varvec{e}}}}_j^\mathrm{T}Y_k^\mathrm{T}{\hat{{{\varvec{v}}}}}|^2\right] = \Vert Y_k\Vert _\mathrm{F}^2 - \Vert Y_k^\mathrm{T}{\hat{{{\varvec{v}}}}}\Vert _{\ell _2}^2 }\nonumber \\ \end{aligned}$$

over ${\hat{{{\varvec{v}}}}} \in S_k$ with $\Vert {\hat{{{\varvec{v}}}}}\Vert _{\ell _2} = 1$ is equivalent to maximizing the Rayleigh quotient ${{\varvec{v}}}^\mathrm{T}Y_kY_k^\mathrm{T}{{\varvec{v}}}/{{\varvec{v}}}^\mathrm{T}{{\varvec{v}}} = \Vert Y_k^\mathrm{T}{{\varvec{v}}}\Vert _{\ell _2}^2/\Vert {{\varvec{v}}}\Vert _{\ell _2}^2$ over the same set, ${\hat{{{\varvec{v}}}}}_k$ in (4.4) is the eigenvector of $\frac{1}{n}Y_\mathrm{c}Y_\mathrm{c}^\mathrm{T}$ with the *k*th largest eigenvalue $\lambda _k=\Vert {\hat{{{\varvec{v}}}}}_k^\mathrm{T}Y_\mathrm{c}/\sqrt{n}\Vert _{\ell _2}^2= \Vert {\hat{{{\varvec{v}}}}}_k^\mathrm{T}Y_k/\sqrt{n}\Vert _{\ell _2}^2$ as shown in (4.4).

We now aim for an appropriate $\ell _1$-variant of (4.4)–(4.5). The proposed approach to determine the orthogonal matrix *V* and the diagonal matrix $\Lambda$ in (2.5) is to replace the sum of squared norms in (4.5) with a sum of norms in (4.8) below. The approach is reminiscent of (3.4) in the sense that while a geometric median *point* is selected by (3.4), a geometric median *line* is determined by (4.7). As for (4.5), let $V_k$ be given by (4.1) and $Y_k$ by (4.2). Then given $S_k$ according to (4.3), let ${{\varvec{v}}}_k$ and $\lambda _k$ be determined inductively by

4.7 $$\begin{aligned} {\hat{{{\varvec{v}}}}}_k= & {} \frac{{{\varvec{v}}}_k}{\Vert {{\varvec{v}}}_k\Vert _{\ell _2}}, \quad {{\varvec{v}}}_k = {\mathop {\hbox {argmin}}\limits _{{{\varvec{v}}}\in S_k}}\, H_k({{\varvec{v}}}), \nonumber \\ \lambda _k= & {} \Vert {\hat{{{\varvec{v}}}}}_k^\mathrm{T}Y_k/\sqrt{n}\Vert _{\ell _1}, \quad k=1,\ldots ,m, \end{aligned}$$

where

4.8 $$\begin{aligned} H_k({{\varvec{v}}})= & {} \sum _{j=1}^n \Big \Vert \left( \frac{{{\varvec{v}}}{{\varvec{v}}}^\mathrm{T}}{\Vert {{\varvec{v}}}\Vert _{\ell _2}^2}-I \right) Y_k{\hat{{\varvec{e}}}}_j \Big \Vert _{\ell _2}, \quad {{\varvec{v}}}\ne {{\varvec{0}}},\nonumber \\ H_k({{\varvec{0}}})= & {} \sum _{j=1}^n \Vert Y_k{\hat{{\varvec{e}}}}_j\Vert _{\ell _2}. \end{aligned}$$

The robustness of the $\ell _1$ measure in $H_k$ in relation to the $\ell _2$ measure in ${\tilde{H}}_k$ is highlighted by the following simple example, which is illustrated in Fig. 4. Here the data are given by

4.9 $$\begin{aligned} Y = \left[ \begin{array}{ccccc} 0 &{} \frac{1}{2} &{} 1 &{} 0 &{} 1 \\ 0 &{} \frac{1}{2} &{} 1 &{} \frac{1}{2} &{} \frac{1}{2} \end{array} \right] \end{aligned}$$

marked with $\cdot$ in Fig. 4a. The points $(0,\frac{1}{2}),(1,\frac{1}{2})$ may be regarded as outliers from points otherwise lying on the line between (0, 0) and (1, 1). The $\ell _2$ data axes are given by (4.4) and are shown in Fig. 4a as dotted line segments. The $\ell _1$ data axes are given by (4.7) and are shown in Fig. 4a as solid line segments. The landscape for the merit function $H_1(\theta )= H_1({\hat{{{\varvec{v}}}}}(\theta ))$ of (4.8) with ${\hat{{{\varvec{v}}}}}(\theta ) = (\cos (\theta ),\sin (\theta )$ is shown in Fig. 4b, and the minimum is marked with $\Box$; see Remark 2 concerning the regularity of the merit function.

**Fig. 5:**
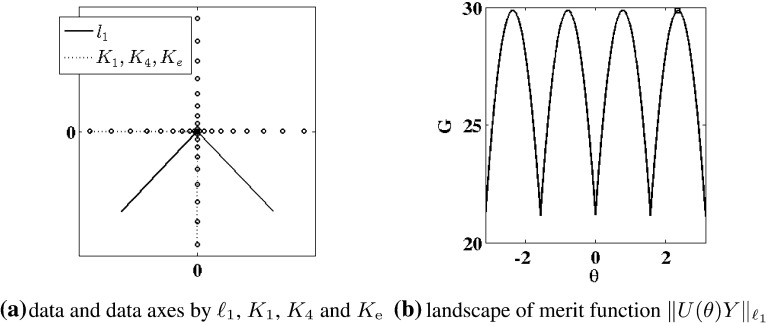
The $\ell _1$ (*solid*) and $K_2$, $K_4$, and $K_\mathrm{e}$ (*dotted*, i.e., identical) data axes were obtained by maximizing the measures in (5.5). The results are compared in **a**, where the data of (5.10) are shown with $\cdot$. Shown in **b** is the landscape of the merit function $\Vert U(\theta )Y\Vert _{\ell _1}$ which is maximized at $\Box$

The data axes defined by (4.7) are computed by the following scheme. For $\tau >0$ and $\rho >0$, compute iteratively $D_l{\hat{{\varvec{e}}}}_j \in S_k$, $j=1,\ldots ,n$, and $\hat{\mathbf{v}}_l\in S_k$ with $\Vert D_0{\hat{{\varvec{e}}}}_j\Vert _{\ell _2} \le 1, j=1,\ldots ,n$, and $\Vert \hat{\mathbf{v}}_0\Vert _{\ell _2}=1$,

4.10 $$\begin{aligned} D_{l+1}{\hat{{\varvec{e}}}}_j= & {} \frac{(\hat{\mathbf{v}}_l \hat{\mathbf{v}}_l^\mathrm{T}-I)(\tau Y_k{\hat{{\varvec{e}}}}_j-D_l{\hat{{\varvec{e}}}}_j)}{1+\tau \Vert (\hat{\mathbf{v}}_l\hat{\mathbf{v}}_l^\mathrm{T}-I)Y_k{\hat{{\varvec{e}}}}_j\Vert _{\ell _2}}, \quad j=1,\ldots ,n \nonumber \\ \end{aligned}$$

4.11 $$\begin{aligned} \hat{\mathbf{v}}_{l+1}= & {} \frac{\mathbf{v}_{l+1}}{\Vert \mathbf{v}_{l+1}\Vert _{\ell _2}} \quad \text{ with }\quad \mathbf{v}_{l+1} = \hat{\mathbf{v}}_l \nonumber \\&- \rho \sum _{j=1}^n \frac{(\hat{\mathbf{v}}_l^\mathrm{T}Y_k{\hat{{\varvec{e}}}}_j) (\tau Y_k{\hat{{\varvec{e}}}}_j - D_l{\hat{{\varvec{e}}}}_j)}{1 + \tau \Vert (\hat{\mathbf{v}}_l\hat{\mathbf{v}}_l^\mathrm{T}-I) Y_k{\hat{{\varvec{e}}}}_j\Vert _{\ell _2}}. \end{aligned}$$

The motivation for this iteration and its convergence analysis are given in Sect. 7. Then the *k*th components of *V* and $\Lambda$ are given, respectively, by taking the limit

4.12 $$\begin{aligned} {{\varvec{v}}}_k = \lim _{l\rightarrow \infty } \hat{\mathbf{v}}_l \end{aligned}$$

and setting

4.13 $$\begin{aligned} \lambda _k = R_k({{\varvec{v}}}_k). \end{aligned}$$

After these calculations have been completed for $k=1,\ldots ,m$, the $\ell _1$ sphered data are given by $Y_\mathrm{s} = \Lambda ^{-\frac{1}{2}}V^\mathrm{T}Y_\mathrm{c}$, the counterpart to (2.8).

Since the minimization problems (4.7) are performed over progressively smaller subspaces $S_k$, it follows that $\lambda _1\ge \lambda _2 \ge \cdots \ge \lambda _m$. Thus, as in Sect. 2, the $\ell _1$-variation $\lambda _i$ of the data $Y_\mathrm{c}$ along the axis ${{\varvec{v}}}_i$ is larger than the $\ell _1$-variation $\lambda _j$ along the axis ${{\varvec{v}}}_j$ for $i<j$. To select only the $r<m$ components with respect to which the data have the most $\ell _1$-variation, define the projected data $Y_P \approx Y$ by the counterpart to (2.11) where the matrices ${\bar{Y}}$, *V*, and $\Lambda$ are now determined by $\ell _1$ measures while the projector $P \in \mathbb {R}^{r\times m}$ is defined as before with entries $P_{i,j} = \delta _{i,j}$.

As the columns of *V* are determined sequentially, the earlier components of *y* are more strongly separated from other later components. Alternatively, all components of *y* may be estimated with roughly equal quality by determining all columns of *V* simultaneously through minimizing a sum of functionals of the form (4.8) for each column under the constraint that *V* be orthogonal [10, 17].

In case the data are undersampled and $n<m$, the steps outlined in this section must be modified as follows. Let ${\bar{Y}}$ and $Y_\mathrm{c}$ be determined as described following (3.4). Then ${\hat{V}} \in \mathbb {R}^{m\times n}$ is determined by solving (4.7) but for $k=1,\ldots ,n$ where $S_1$ is the range of $Y_\mathrm{c}$. With $\lambda _k = R_k({{\varvec{v}}}_k)$, set ${\hat{\Lambda }} = \text{ diag }\{\lambda _k\}_{k=1}^n$. The sphered data are then given by ${\hat{Y}}_\mathrm{s} = {\hat{\Lambda }}^{-\frac{1}{2}}{\hat{V}}^\mathrm{T}Y_\mathrm{c} \in \mathbb {R}^{n\times n}$. Finally, for a projector $P \in \mathbb {R}^{n\times n}$, (2.11) becomes $Y_P = {\bar{Y}} + {\hat{V}}{\hat{\Lambda }}^{\frac{1}{2}} P^\mathrm{T}P{\hat{\Lambda }}^{-\frac{1}{2}}{\hat{V}}^\mathrm{T}(Y-{\bar{Y}})$.

**Fig. 6:**
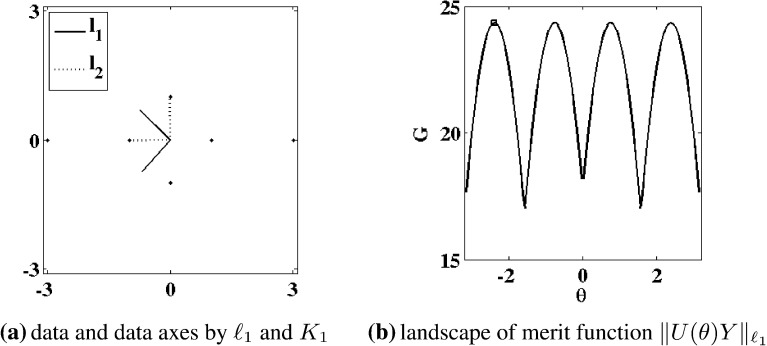
The $\ell _1$ (*solid*) and $\ell _2$ (*dotted*) data axes are compared in **a**, where the data of (5.10) are shown with $\cdot$. Shown in **b** is the landscape of the merit function $G_1$ in (5.9)

## $\ell _1$ Approach to ICA

While the Kurtosis has been used as a measure of Gaussianity in (2.18) to determine independent components, an alternative measure of independence is proposed here which is more robust in the presence of outliers. This approach targets independence directly in a manner which can be illustrated in terms of the example shown below in Fig. 5. Here the data are given by

5.1 $$\begin{aligned} Y= & {} \left[ \begin{array}{rrrrrrrrrr} -1 &{} \ldots &{} \sigma \left( \frac{i}{m}\right) |\frac{i}{m}|^k &{} \ldots &{} 1 &{} 0 &{} \ldots &{} &{} \ldots &{} 0 \\ 0 &{} \ldots &{} &{} \ldots &{} 0 &{} -1 &{} \ldots &{} \sigma (\frac{i}{m})|\frac{i}{m}|^k &{} \ldots &{} 1 \\ \end{array} \right] , \nonumber \\&\quad \begin{array}{c} m = 20, k=2 \\ i=-m,\ldots ,m \end{array} \end{aligned}$$

where $\sigma (t) = \text{ sign }(t)$. Let these data represent a realization of a random vector ${y} \in \mathbb {R}^2$ satisfying

5.2 $$\begin{aligned} {P}({y}=Y{\hat{{\varvec{e}}}}_i)= {P}({ y}=Y{\hat{{\varvec{e}}}}_j)=\frac{1}{2m}, \quad ({\hat{{\varvec{e}}}}_i)_j=\delta _{ij}, \end{aligned}$$

where *P* denotes the probability. In order that the rotation dependent random vector

5.3 $$\begin{aligned} {x}(\theta )= & {} \{{x}_i(\theta )\}_{i=1}^2, \quad {x}(\theta )=U(\theta ){y}, \quad U(\theta )\nonumber \\= & {} \left[ \begin{array}{rr} \cos (\theta ) &{} \sin (\theta ) \\ -\sin (\theta ) &{} \cos (\theta ) \end{array} \right] \end{aligned}$$

satisfy the independence condition,

5.4 $$\begin{aligned} {P}({x}_1(\theta )= & {} \alpha \text{ and } { x}_2(\theta )=\beta )\nonumber \\= & {} {P}({x}_1(\theta )=\alpha )\cdot {P}({x}_2(\theta )=\beta ), \quad \forall \alpha ,\beta \in \mathbb {R},\nonumber \\ \end{aligned}$$

a rotation angle $\theta =\pi /4+k\pi /2$, $k\in \mathbb {Z}$, must be chosen. For the determination of the proper rotation, four different measures of independence are compared in Fig. 5,

5.5 $$\begin{aligned}&\Vert U(\theta )Y\Vert _{\ell _1}, \quad K_1^2(U(\theta )Y), \nonumber \\&\quad K_4^2(U(\theta )Y), \quad \text{ and } \quad K_\mathrm{e}^2(U(\theta )Y), \end{aligned}$$

where $K_m$ is given by (2.13) and (see [14])

5.6 $$\begin{aligned} K_\mathrm{e}({x})= & {} {E}[\exp (-|{x}-{E}[{ x}]|^2)/2]\nonumber \\&- 1/\sqrt{1+{E}[|{x}-{E}[{x}]|^2]}. \end{aligned}$$

The data axes obtained by maximizing the last three measures in (5.5) are identical and are shown in Fig. 9a as dotted line segments. The data axes obtained by maximizing the first measure in (5.5) are shown in Fig. 9a as solid line segments. The landscape for the merit function $\Vert U(\theta )Y\Vert _{\ell _1}$ of (5.5) is shown in Fig. 9b and the maximum is marked with $\Box$. Only the first measure in (5.5) is maximized at a desired angle as shown in the landscape of Fig. 9b. All other measures are maximized at a multiple of $\pi$. The correct rotation is also obtained using (5.8) for the data presented in Sect. 9. While a more detailed statistical analysis is intended for a future work, it is presently on the basis of these examples that the measure shown below in (5.8) is proposed to determine the rotation matrix *U* of (2.14).

To achieve maximally independent rows of $X_\mathrm{c} = UY_\mathrm{s}$, the rows of the orthogonal matrix $U = \{\hat{{{\varvec{u}}}}_i^\mathrm{T}\}_{i=1}^m$ are determined as follows. Define $U_l = \{\hat{{{\varvec{u}}}}_1,\ldots ,{\hat{{{\varvec{u}}}}}_l\}^\mathrm{T}$ as in (2.15) and the projected data $Y_l = (I-U_{l-1}^\mathrm{T}U_{l-1})Y_\mathrm{s}$, $l=2,\ldots ,m$, $Y_1 = Y_\mathrm{s}$, as in (2.16). Let $T_l = {\mathcal R}(Y_l)$ where ${\mathcal R}$ denotes the range. For convenience, it is assumed here that the data $Y_\mathrm{s}$ have maximal rank so that $T_1 = \mathbb {R}^m$. Then ${{\varvec{0}}} = {{\varvec{u}}}^\mathrm{T}(I-U_{l-1}^\mathrm{T}U_{l-1})Y_\mathrm{s} = {{\varvec{u}}}^\mathrm{T}Y_l$ is equivalent to ${{\varvec{u}}} = U_{l-1}^\mathrm{T}U_{l-1}{{\varvec{u}}}$, which is equivalent to ${{\varvec{u}}} = {{\varvec{0}}}$ exactly when $U_{l-1}{{\varvec{u}}} = {{\varvec{0}}}$. Hence,

5.7 $$\begin{aligned} T_l = {\mathcal R}(U_{l-1}^\mathrm{T})^\perp , \quad l=2,\ldots ,m, \quad T_1 = \mathbb {R}^m. \end{aligned}$$

Given $T_l$, let the *l*th column of $U^\mathrm{T}$ be determined inductively by

5.8 $$\begin{aligned} {\hat{{{\varvec{u}}}}}_l = \frac{\mathbf{u}_l}{\Vert \mathbf{u}_l\Vert _{\ell _2}}, \quad \mathbf{u}_l = {\mathop {\hbox {argmax}}\limits _{\mathbf{u}\in T_l}}\, G_l(\mathbf{u}), \end{aligned}$$

where

5.9 $$\begin{aligned} G_l(\mathbf{u}) = \frac{\Vert \mathbf{u}^\mathrm{T}Y_l\Vert _{\ell _1}}{\Vert \mathbf{u}\Vert _{\ell _2}}, \quad \mathbf{u}\ne {{\varvec{0}}}, \quad G_l({{\varvec{0}}}) = 0. \end{aligned}$$

In this way, the rows of *U* are determined sequentially so that the earlier components of *x* are more strongly separated from other later components. Alternatively, all components of *x* may be estimated with roughly equal quality by determining all rows of *U* simultaneously through maximizing a sum of functionals of the form (5.9) for each row under the constraint that *U* be orthogonal [14]. Once matrices *U*, $\Lambda$, and *V* are determined, the source samples are estimated according to (2.4) and (2.5).

The robustness of the measure $G_l$ in (5.9) in relation to the measure *F* in (2.20) is highlighted by the following simple example, which is illustrated in Fig. 6. Here the data *Y* are given by

5.10 $$\begin{aligned} \displaystyle Y_x= & {} \left[ \begin{array}{rrrrrrrrrr} +1 &{} +1 &{} +1 &{} +1 &{} +1 &{} -1 &{} -1 &{} -1 &{} -1 &{} -1 \\ 0 &{} 0 &{} 0 &{} 0 &{} 0 &{} 0 &{} 0 &{} 0 &{} 0 &{} 0 \\ \end{array} \right] \nonumber \\ Y_y= & {} \left[ \begin{array}{rr} 0 &{} 1 \\ 1 &{} 0 \\ \end{array} \right] Y_x, \quad Y_\mathrm{o} = \left[ \begin{array}{rr} 3 &{} -3 \\ 0 &{} 0 \\ \end{array} \right] , \quad Y = \left[ \right. Y_x \quad Y_y \quad Y_\mathrm{o} \left. \right] \nonumber \\ \end{aligned}$$

marked with $\cdot$ in Fig. 6a, and the points $(\pm 3,0)$ may be regarded as outliers from points otherwise lying at the diamond vertices $\{(0,\pm 1),(\pm 1,0)\}$. The $\ell _2$ data axes are given by (2.18) and are shown in Fig. 6a as dotted line segments. The $\ell _1$ data axes are given by (5.8) and are shown in Fig. 6a as solid line segments, where the landscape for the merit function $G_1$ of (5.9) is shown in Fig. 6b and the maximum is marked with $\Box$.

The vectors defined by (5.8) are computed by the following scheme. For $\tau > 0$, compute iteratively $\hat{\mathbf{u}}_k \in T_l$ with $\Vert \hat{\mathbf{u}}_0\Vert _{\ell _2} = 1$,

5.11 $$\begin{aligned} \hat{\mathbf{u}}_{k+1}= & {} \frac{\mathbf{u}_{k+1}}{\Vert \mathbf{u}_{k+1}\Vert _{\ell _2}} \quad \text{ with }\nonumber \\ \mathbf{u}_{k+1}= & {} \hat{\mathbf{u}}_k + \tau \left[ Y_l\, \sigma (Y_l^\mathrm{T}\hat{\mathbf{u}}_k) - \hat{\mathbf{u}}_k \Vert \hat{\mathbf{u}}_k^\mathrm{T}Y_l\Vert _{\ell _1} \right] , \end{aligned}$$

where

5.12 $$\begin{aligned} \sigma (t)= & {} \text{ sign }(t) \quad \text{ for } \quad t\in \mathbb {R}, \qquad \sigma ({{\varvec{v}}})=\{\sigma (v_j)\}_{j=1}^n \quad \text{ for } \nonumber \\ {{\varvec{v}}}= & {} \{v_j\}_{j=1}^n \in \mathbb {R}^n. \end{aligned}$$

The motivation for this iteration and its convergence analysis are given in Sect. 8. The *l*th column of $U^\mathrm{T}$ is given by taking the limit,

5.13 $$\begin{aligned} {\hat{{{\varvec{u}}}}}_l = \lim _{k\rightarrow \infty } \hat{\mathbf{u}}_k. \end{aligned}$$

After these calculations have been completed for $l=1,\ldots ,m$, the $\ell _1$ maximally independent data are given by $X_\mathrm{c} = UY_\mathrm{s}$, the counterpart to (2.14).

To select the $$r<m$$ desired independent components $$\{q_1,\ldots ,q_r\}$$$$\subset $$$$\{1,\ldots ,m\}$$, define the projected data $$Y_Q \approx Y$$ by the counterpart to (2.21) where the matrices $${\bar{Y}}$$, *V*, $$\Lambda $$, and *U* are now determined by $$\ell _1$$ measures, while the projector $$Q \in \mathbb {R}^{r\times m}$$ is defined as before with entries $$Q_{ij} = \delta _{q_i,j}$$.

In case the data are undersampled and $n<m$, let ${\hat{V}} \in \mathbb {R}^{m\times n}$, ${\hat{Y}}_\mathrm{s},{\hat{\Lambda }} \in \mathbb {R}^{n\times n}$ be given as described at the end of Sect. 3. Then, the sphered data ${\hat{Y}}_\mathrm{s}$ are transformed by the rotation matrix ${\hat{U}} \in \mathbb {R}^{n\times n}$ maximizing independence of the rows of ${\hat{X}}_\mathrm{c} = {\hat{U}}{\hat{Y}}_\mathrm{s} \in \mathbb {R}^{n\times n}$. Thus, ${\hat{X}} = {\hat{U}}{\hat{\Lambda }}^{-\frac{1}{2}}{\hat{V}}^\mathrm{T}Y$ gives the maximum number of independent components which can be determined from the undersampled data. Finally, for a projector $Q \in \mathbb {R}^{n\times n}$, (2.21) becomes $Y_Q = {\bar{Y}} + {\hat{V}}{\hat{\Lambda }}^{\frac{1}{2}}{\hat{U}}^\mathrm{T} Q^\mathrm{T}Q{\hat{U}}{\hat{\Lambda }}^{-\frac{1}{2}}{\hat{V}}^\mathrm{T}(Y-{\bar{Y}})$.

## Convergence of the Iterative Scheme for the $\ell _1$ Mean

The analysis of the scheme (3.6)–(3.7) begins by establishing basic properties for the minimization problem (3.4), i.e., the determination of the geometric median. As indicated in Sect. 1, a proof of uniqueness of the geometric median is provided here which is shorter than that found in [21] or [19] and is based on strict convexity of the functional *M*. Also, a possibly novel characterization of a solution is provided in case the columns of *Y* are collinear, which means that *Y* can be expressed in the form

6.1 $$\begin{aligned} Y= & {} {{\varvec{a}}} {{\varvec{e}}}^\mathrm{T} + {{\varvec{b}}}{{\varvec{y}}}^\mathrm{T}, \text {where }\nonumber \\ {{\varvec{e}}}= & {} (1,\ldots ,1 )^\mathrm{T},\, {{\varvec{a}}},{{\varvec{b}}} \in \mathbb {R}^m,\, {{\varvec{e}}},{{\varvec{y}}} \in \mathbb {R}^n. \end{aligned}$$

### **Lemma 1**

If the columns of $Y \in \mathbb {R}^{m\times n}$ are not collinear, then *M* is strictly convex.

### *Proof*

The mapping *M* is the sum of convex mappings and hence convex itself. If *M* is not strictly convex, then there are vectors ${{{\varvec{\mu }}}_\mathbf{1}},{{{\varvec{\mu }}}_\mathbf{2}} \in \mathbb {R}^m$, and ${\varvec{\overline{\mu }}}= \overline{\alpha } {{{\varvec{\mu }}}_\mathbf{1}} + (1 - \overline{\alpha }) {{{\varvec{\mu }}}_\mathbf{2}}, \text { with } \overline{\alpha } \in (0,1)$ such that

$$\begin{aligned} M({\varvec{\overline{\mu }}}) = \overline{\alpha } M ({{{\varvec{\mu }}}_\mathbf{1}}) + (1 - \overline{\alpha }) M ({{{\varvec{\mu }}}_\mathbf{2}}) \end{aligned}$$

and thus the mapping $\alpha \rightarrow M (\alpha {{{\varvec{\mu }}}_\mathbf{1}} + (1- \alpha ){{{\varvec{\mu }}}_\mathbf{2}}),\, \alpha \in (0,1),$ is affine. Let $\hat{\alpha } \in (0,1)$ be such that $\hat{{{\varvec{\mu }}}} = \hat{\alpha } {{{\varvec{\mu }}}_\mathbf{1}} + (1-\hat{\alpha }){{{\varvec{\mu }}}_\mathbf{2}}$ does not coincide with any of the columns $Y {\hat{{\varvec{e}}}}_i$. Then we find the Hessian

6.2 $$\begin{aligned} \nabla ^2 M ({\hat{{\varvec{\mu }}}})= & {} \sum _{i=1}^{n} \frac{1}{\Vert {\hat{{\varvec{\mu }}}}-Y{\hat{{\varvec{e}}}}_i\Vert _{\ell _2}^3} \big (\Vert {\hat{{\varvec{\mu }}}} \nonumber \\&\quad - Y{\hat{{\varvec{e}}}}_i\Vert _{\ell _2}^2 I - ({\hat{{\varvec{\mu }}}} - Y{\hat{{\varvec{e}}}}_i)({\hat{{\varvec{\mu }}}} - Y{\hat{{\varvec{e}}}}_i)^\mathrm{T} \big ), \end{aligned}$$

and hence for any ${{\varvec{x}}} \in \mathbb {R}^m$

6.3 $$\begin{aligned} {{\varvec{x}}}^\mathrm{T} \nabla ^2 M ({\hat{{\varvec{\mu }}}}) {{\varvec{x}}}= & {} \sum _{i=1}^{n} \frac{1}{\Vert {\hat{{\varvec{\mu }}}}-Y{\hat{{\varvec{e}}}}_i\Vert _{\ell _2}^3} \big (\Vert {\hat{{\varvec{\mu }}}}\nonumber \\&- Y{\hat{{\varvec{e}}}}_i\Vert _{\ell _2}^2 \, \Vert {{\varvec{x}}}\Vert _{\ell _2}^2 - |({\hat{{\varvec{\mu }}}} - Y {\hat{{\varvec{e}}}}_i)^\mathrm{T} {{\varvec{x}}}|^2 \big ). \end{aligned}$$

Note that $|({\hat{{\varvec{\mu }}}} - Y {\hat{{\varvec{e}}}}_i)^\mathrm{T} {{\varvec{x}}}| \le \Vert {\hat{{\varvec{\mu }}}} - Y {\hat{{\varvec{e}}}}_i\Vert _{\ell _2} \, \Vert {{\varvec{x}}}\Vert _{\ell _2}.$ If $|({\hat{{\varvec{\mu }}}} - Y {\hat{{\varvec{e}}}}_i)^\mathrm{T} {{\varvec{x}}}| = \Vert {\hat{{\varvec{\mu }}}} - Y {\hat{{\varvec{e}}}}_i\Vert \, \Vert {{\varvec{x}}}\Vert$ for all $i = 1,\ldots ,n,$ then there exist $b_i\in \mathbb {R}$ such that ${\hat{{\varvec{\mu }}}} - Y {\hat{{\varvec{e}}}}_i = b_i{{\varvec{x}}},$ for $i=1,\ldots ,n$. Thus there exists ${{\varvec{b}}} \in \mathbb {R}^n$ such that $Y = {{\varvec{\mu }}} {{\varvec{e}}}^\mathrm{T} - {{\varvec{b}}}{{\varvec{x}}}^\mathrm{T}$ which contradicts the assumption. Hence there exists at least one index *i* such that $|({\hat{{\varvec{\mu }}}} - Y {\hat{{\varvec{e}}}}_i)^\mathrm{T} {{\varvec{x}}}| < \Vert {\hat{{\varvec{\mu }}}} - Y {\hat{{\varvec{e}}}}_i\Vert _{\ell _2} \, \Vert {{\varvec{x}}}\Vert _{\ell _2}$ and thus ${{\varvec{x}}}^\mathrm{T} \nabla ^2 M({\hat{{\varvec{\mu }}}}) {{\varvec{x}}} > 0.$ This contradicts that $\alpha \rightarrow M (\alpha {{{\varvec{\mu }}}_\mathbf{1}} + (1 -\alpha ) {{{\varvec{\mu }}}_\mathbf{2}})$ is affine at $\hat{\alpha }$. $\square$

### **Lemma 2**

If the columns of $Y \in \mathbb {R}^{m\times n}$ are collinear, then *M* is minimized (not necessarily uniquely) by ${{\varvec{\mu }}}^\star = {{\varvec{a}}} + {{\varvec{b}}} \cdot \text { median } ({{\varvec{y}}})$. If the columns of *Y* are not collinear, there exists a unique ${{\varvec{\mu }}}^\star \in \mathbb {R}^m$ minimizing *M*.

### *Proof*

Existence of a solution ${{\varvec{\mu }}}^* \in \mathbb {R}^m$ follows by standard subsequential limit arguments. If the columns of *Y* are not collinear, uniqueness of the solution ${{\varvec{\mu }}}^\star$ follows from strict convexity of ${{\varvec{\mu }}} \rightarrow M ({{\varvec{\mu }}})$.

Suppose next that the columns of *Y* are collinear so that $Y = {{\varvec{a}}}{{\varvec{e}}}^\mathrm{T} + {{\varvec{b}}}{{\varvec{y}}}^\mathrm{T}$. Set $\nu = \Vert {{\varvec{b}}}\Vert _{\ell _2}$ and ${\varvec{w}} = {{\varvec{b}}} + \nu {\hat{{\varvec{e}}}}_1$ where $({\hat{{\varvec{e}}}}_1)_i = \delta _{i1}$. Then the Householder transformation

6.4 $$\begin{aligned} U = I - 2 \frac{{\varvec{w}}{\varvec{w}}^\mathrm{T}}{\Vert {\varvec{w}}\Vert _{\ell _2}^2} \end{aligned}$$

is orthogonal and satisfies

6.5 $$\begin{aligned} U{{\varvec{b}}} = -\nu {\hat{{\varvec{e}}}}_1 \end{aligned}$$

as well as $\Vert U{{\varvec{x}}}\Vert _{\ell _2} = \Vert {{\varvec{x}}}\Vert _{\ell _2}$, $\forall {{\varvec{x}}} \in \mathbb {R}^m$. Let an arbitrary ${{\varvec{\mu }}} \in \mathbb {R}^m$ be represented as

6.6 $$\begin{aligned} {{\varvec{\mu }}} ={{\varvec{a}}} + x{{\varvec{b}}}+ \tilde{{{\varvec{b}}}}, \quad x = ({{\varvec{\mu }}}-{{\varvec{a}}})^\mathrm{T}{{\varvec{b}}}/\Vert {{\varvec{b}}}\Vert _{\ell _2}, \quad {{\varvec{b}}}^\mathrm{T}\tilde{{{\varvec{b}}}} = 0\nonumber \\ \end{aligned}$$

and note that

6.7 $$\begin{aligned} (U{{\varvec{b}}})^\mathrm{T}(U\tilde{{{\varvec{b}}}}) = {{\varvec{b}}}^\mathrm{T}U^\mathrm{T}U\tilde{{{\varvec{b}}}} = {{\varvec{b}}}^\mathrm{T}\tilde{{{\varvec{b}}}} = 0. \end{aligned}$$

With (6.5)–(6.7), the merit function can be written as

6.8 $$\begin{aligned} M({{\varvec{\mu }}})= & {} \displaystyle \sum _{j=1}^n \Vert {{\varvec{\mu }}}-{{\varvec{a}}}-{{\varvec{b}}}y_j\Vert _{\ell _2}\nonumber \\= & {} \sum _{j=1}^n \Vert U({{\varvec{\mu }}}-{{\varvec{a}}}-{{\varvec{b}}}y_j)\Vert _{\ell _2} \nonumber \\= & {} \sum _{j=1}^n \Vert U(\tilde{{{\varvec{b}}}}+(x-y_j){{\varvec{b}}})\Vert _{\ell _2}\nonumber \\= & {} \displaystyle \sum _{j=1}^n \left[ \Vert U\tilde{{{\varvec{b}}}}\Vert _{\ell _2}^2\right. \nonumber \\&\left. +\, 2(x-y_j)(U{{\varvec{b}}})^\mathrm{T}(U\tilde{{{\varvec{b}}}}) +|x-y_j|^2\nu ^2 \right] ^{\frac{1}{2}} \nonumber \\= & {} {\displaystyle \sum _{j=1}^n \left[ \Vert U\tilde{{{\varvec{b}}}}\Vert _{\ell _2}^2+|x-y_j|^2\nu ^2 \right] ^{\frac{1}{2}}. } \end{aligned}$$

Once the merit function has been reduced to this one-dimensional form, it is readily seen that $M({{\varvec{\mu }}}) \ge M({{\varvec{a}}}+\gamma {\varvec{b)}}$ for $\gamma = \text{ median }({{\varvec{y}}})$, and hence *M* is minimized at ${{\varvec{a}}}+\gamma {{\varvec{b}}}$. That the minimizer is not necessarily unique can be seen from the case that *n* is even and the components of ${{\varvec{y}}}$ are distinctly ascending, so *M* has the same value $M({{\varvec{a}}}+\gamma {{\varvec{b}}})$ at all points ${{\varvec{a}}}+{{\varvec{b}}}[ty_{n/2}+(1-t)y_{n/2+1}]$, $t\in [0,1]$. $\square$

### **Lemma 3**

The first-order necessary optimality condition for a minimizer ${{\varvec{\mu }}}^\star$ of *M* over $\mathbb {R}^m$ is that there is $D^\star \in \mathbb {R}^{m\times n}$ satisfying,

6.9 $$\begin{aligned}&{{\varvec{\mu }}}^\star -Y{\hat{{\varvec{e}}}}_j = \Vert {{\varvec{\mu }}}^\star -Y{\hat{{\varvec{e}}}}_j\Vert _{\ell _2}D^\star {\hat{{\varvec{e}}}}_j, \nonumber \\&\Vert D^\star {\hat{{\varvec{e}}}}_j\Vert _{\ell _2}\le 1, \quad j=1,\ldots ,n, \quad \sum _{j=1}^n D^\star {\hat{{\varvec{e}}}}_j = {{\varvec{0}}}. \end{aligned}$$

### *Proof*

The necessary optimality condition for a minimizer ${{\varvec{\mu }}}^\star$ is that $0 \in \partial M({{\varvec{\mu }}}^\star )$. By the chain rule (see, e.g., [2], p. 233), the subdifferential of *M* is given by the sum of the respective subdifferentials,

6.10 $$\begin{aligned} \partial M({{\varvec{\mu }}}) = \sum _{j=1}^n \partial \Vert {{\varvec{\mu }}}-Y{\hat{{\varvec{e}}}}_j\Vert _{\ell _2}. \end{aligned}$$

Thus, there exist ${\varvec{d}}_j^\star \in \partial \Vert {{\varvec{\mu }}}^\star -Y{\hat{{\varvec{e}}}}_j\Vert _{\ell _2}$, $j=1,\ldots ,n$, satisfying

6.11 $$\begin{aligned} \sum _{j=1}^n {\varvec{d}}_j^\star = 0, \end{aligned}$$

and

6.12 $$\begin{aligned} \partial \Vert {{\varvec{\mu }}}-Y{\hat{{\varvec{e}}}}_j\Vert _{\ell _2} = \left\{ \begin{array}{cl} {\displaystyle \frac{{{\varvec{\mu }}}-Y{\hat{{\varvec{e}}}}_j}{\Vert {{\varvec{\mu }}}-Y{\hat{{\varvec{e}}}}_j\Vert _{\ell _2}}}, &{} {{\varvec{\mu }}} \ne Y{\hat{{\varvec{e}}}}_j \\ B(0,1), &{} {{\varvec{\mu }}} = Y{\hat{{\varvec{e}}}}_j, \\ \end{array} \right. \end{aligned}$$

where *B*(0, 1) is the unit ball. Combining these facts, we have

6.13 $$\begin{aligned} ({{\varvec{\mu }}}^\star -Y{\hat{{\varvec{e}}}}_j) = \Vert {{\varvec{\mu }}}^\star -Y{\hat{{\varvec{e}}}}_j\Vert _{\ell _2}{\varvec{d}}_j^\star , \quad j=1,\ldots ,n. \end{aligned}$$

The claim (6.9) follows with $D^\star = \{{\varvec{d}}_1^\star ,\ldots ,{\varvec{d}}_n^\star \}$. $\square$

Turning to the iteration (3.6)–(3.7), we observe that if convergence to a fixed point $\{D^\star ,{{\varvec{\mu }}}^\star \}$ can be guaranteed, then from (3.6) we have that

6.14 $$\begin{aligned} D^\star {\hat{{\varvec{e}}}}_j \Vert {{\varvec{\mu }}}^\star - Y_j\Vert _{\ell _2} = {{\varvec{\mu }}}^\star - Y{\hat{{\varvec{e}}}}_j, \quad j=1,\ldots ,n. \end{aligned}$$

From (3.7) we have

6.15 $$\begin{aligned} \sum _{j=1}^{n} \frac{D^\star {\hat{{\varvec{e}}}}_j+\tau ({{\varvec{\mu }}}^\star -Y{\hat{{\varvec{e}}}}_j)}{1 + \tau \Vert {{\varvec{\mu }}}^\star - Y{\hat{{\varvec{e}}}}_j\Vert _{\ell _2}} = 0. \end{aligned}$$

Combining (6.14) and (6.15) gives

6.16 $$\begin{aligned} 0= \sum _{j=1}^{n} \frac{D^\star {\hat{{\varvec{e}}}}_j+\tau D^\star {\hat{{\varvec{e}}}}_j\Vert {{\varvec{\mu }}}^\star - Y{\hat{{\varvec{e}}}}_j\Vert _{\ell _2}}{1 + \tau \Vert {{\varvec{\mu }}}^\star - Y_j\Vert _{\ell _2}} = \sum _{j=1}^{n} D^\star {\hat{{\varvec{e}}}}_j. \end{aligned}$$

Moreover, if $\Vert D_0{\hat{{\varvec{e}}}}_j\Vert _{\ell _2} \le 1, j=1,\ldots ,n,$ then the iterates $\{D_l\}$ also satisfy this bound, and hence $\Vert D^\star {\hat{{\varvec{e}}}}_j\Vert _{\ell _2} \le 1, j=1,\ldots ,n$. Thus $\{D^\star ,{{\varvec{\mu }}}^\star \}$ must satisfy the necessary optimality condition (6.9). The following theorem provides sufficient conditions under which convergence of our algorithm to a fixed point can be guaranteed.

### **Theorem 1**

Suppose that the columns of $Y \in \mathbb {R}^{m\times n}$ are not collinear so that (6.1) does not hold. Let $\{D^\star ,{{\varvec{\mu }}}^\star \}$ satisfy (6.9) with ${{\varvec{\mu }}}^\star \not \in \{Y{\hat{{\varvec{e}}}}_j\}_{j=1}^n$. Then $\{D^\star ,{{\varvec{\mu }}}^\star \}$ is a fixed point for the iteration (3.6)–(3.7), and for $\tau$ sufficiently large, iterates $\{D_l,{{\varvec{\mu }}}_l\}$ converge to this fixed point when $\{D_0,{{\varvec{\mu }}}_0\}$ starts the iteration close enough to $\{D^\star ,{{\varvec{\mu }}}^\star \}$.

### *Proof*

It will first be shown that $\{D^\star ,{{\varvec{\mu }}}^\star \}$ is a fixed point for the iteration (3.6)–(3.7), which is locally asymptotically stable for $\tau$ sufficiently large. Using (6.9) and substituting $D_l = D^\star$ on the right side of (3.6),

6.17 $$\begin{aligned} D_{l+1}{\hat{{\varvec{e}}}}_j= & {} \frac{D^\star {\hat{{\varvec{e}}}}_j+\tau ({{\varvec{\mu }}}^\star - Y{\hat{{\varvec{e}}}}_j)}{1+\tau \Vert {{\varvec{\mu }}}_l-Y{\hat{{\varvec{e}}}}_j\Vert _{\ell _2}}\nonumber \\= & {} \frac{D^\star {\hat{{\varvec{e}}}}_j+\tau \Vert {{\varvec{\mu }}}^\star - Y{\hat{{\varvec{e}}}}_j\Vert _{\ell _2}D^\star {\hat{{\varvec{e}}}}_j}{1+\tau \Vert {{\varvec{\mu }}}_l-Y{\hat{{\varvec{e}}}}_j\Vert _{\ell _2}}\nonumber \\= & {} D^\star {\hat{{\varvec{e}}}}_j, \quad 1\le j\le n \end{aligned}$$

gives $D_{l+1} = D^\star$. Using this result together with (6.9) and setting ${{\varvec{\mu }}}_l = {{\varvec{\mu }}}^\star$ on the right side of (3.7),

6.18 $$\begin{aligned} {{\varvec{0}}}= & {} {\displaystyle \sum _{j=1}^n \frac{D^\star {\hat{{\varvec{e}}}}_j+\tau ({{\varvec{\mu }}}^\star -Y{\hat{{\varvec{e}}}}_j) + \tau ({{\varvec{\mu }}}_{l+1}-{{\varvec{\mu }}}^\star )}{1+\tau \Vert {{\varvec{\mu }}}^\star -Y{\hat{{\varvec{e}}}}_j\Vert _{\ell _2}}} \nonumber \\= & {} \displaystyle \sum _{j=1}^n D^\star {\hat{{\varvec{e}}}}_j + \sum _{j=1}^n \frac{\tau ({{\varvec{\mu }}}_{l+1}-{{\varvec{\mu }}}^\star )}{1+\tau \Vert {{\varvec{\mu }}}^\star -Y{\hat{{\varvec{e}}}}_j\Vert _{\ell _2}}\nonumber \\= & {} ({{\varvec{\mu }}}_{l+1}-{{\varvec{\mu }}}^\star ) \sum _{j=1}^n \frac{\tau }{1+\tau \Vert {{\varvec{\mu }}}^\star -Y{\hat{{\varvec{e}}}}_j\Vert _{\ell _2}} \end{aligned}$$

gives ${{\varvec{\mu }}}_{l+1}={{\varvec{\mu }}}^\star$. Thus, $\{D^\star ,{{\varvec{\mu }}}^\star \}$ is a fixed point of the iteration (3.6)–(3.7). To establish the stability of the fixed point, define

6.19 $$\begin{aligned} {{\varvec{F}}}_j({\varvec{d}}_1,\ldots ,{\varvec{d}}_n,{{\varvec{\mu }}}) = \frac{{\varvec{d}}_j+\tau ({{\varvec{\mu }}}-Y{\hat{{\varvec{e}}}}_j)}{1+\tau \Vert {{\varvec{\mu }}}-Y{\hat{{\varvec{e}}}}_j\Vert _{\ell _2}}, \quad j=1,\ldots ,n\nonumber \\ \end{aligned}$$

and

6.20 $$\begin{aligned}&{{\varvec{G}}}({\varvec{d}}_1,\ldots ,{\varvec{d}}_n,{{\varvec{\mu }}})\nonumber \\&\quad = \sum _{j=1}^n \frac{({\varvec{d}}_j-\tau Y{\hat{{\varvec{e}}}}_j)}{1+ \tau \Vert {{\varvec{\mu }}}-Y{\hat{{\varvec{e}}}}_j\Vert _{\ell _2}} \big / \sum _{j=1}^n \frac{-\tau }{1+ \tau \Vert {{\varvec{\mu }}}-Y{\hat{{\varvec{e}}}}_j\Vert _{\ell _2}}\nonumber \\ \end{aligned}$$

so that (3.6)–(3.7) is given by

6.21 $$\begin{aligned} D_{l+1}{\hat{{\varvec{e}}}}_j= & {} {{\varvec{F}}}_j(D_l{\hat{{\varvec{e}}}}_1,\ldots ,D_l{\hat{{\varvec{e}}}}_n,{{\varvec{\mu }}}_l), \quad j=1,\ldots ,n, \nonumber \\ {{\varvec{\mu }}}_{l+1}= & {} {{\varvec{G}}}(D_{l+1}{\hat{{\varvec{e}}}}_1,\ldots ,D_{l+1}{\hat{{\varvec{e}}}}_n,{{\varvec{\mu }}}_l). \end{aligned}$$

The claimed stability will follow once it is shown that the Jacobian of this mapping evaluated at $\{D^\star ,{{\varvec{\mu }}}^\star \} = \{{\varvec{d}}_1^\star ,\ldots ,{\varvec{d}}_n^\star ,{{\varvec{\mu }}}^\star \}$ has spectral radius less than 1 when $\tau$ is sufficiently large. For (6.19),

6.22 $$\begin{aligned}&\frac{\partial {{\varvec{F}}}_j}{\partial {\varvec{d}}}({\varvec{d}}_1^\star ,\ldots , {\varvec{d}}_n^\star ,{{\varvec{\mu }}}^\star )\nonumber \\&\quad = \frac{I}{1+\tau \Vert {{\varvec{\mu }}}^\star -Y{\hat{{\varvec{e}}}}_j\Vert _{\ell _2}} \mathop {\longrightarrow }\limits ^{\tau \rightarrow \infty } 0 \end{aligned}$$

and

6.23 $$\begin{aligned}&{\displaystyle \frac{\partial {{\varvec{F}}}_j}{\partial {{\varvec{\mu }}}}({\varvec{d}}_1^\star ,\ldots , {\varvec{d}}_n^\star ,{{\varvec{\mu }}}^\star ) } = \displaystyle \frac{\tau I}{1+\tau \Vert {{\varvec{\mu }}}^\star -Y{\hat{{\varvec{e}}}}_j\Vert }\nonumber \\&\quad - \frac{{\varvec{d}}_j^\star +\tau ({{\varvec{\mu }}}^\star -Y {\hat{{\varvec{e}}}}_j)}{[1+\tau \Vert {{\varvec{\mu }}}^\star -Y{\hat{{\varvec{e}}}}_j\Vert _{\ell _2}]^2} \frac{\tau ({{\varvec{\mu }}}^\star -Y{\hat{{\varvec{e}}}}_j)^\mathrm{T}}{\Vert {{\varvec{\mu }}}^\star -Y{\hat{{\varvec{e}}}}_j\Vert _{\ell _2}} \nonumber \\&\quad \mathop {\longrightarrow }\limits ^{\tau \rightarrow \infty } \displaystyle \frac{1}{\Vert {{\varvec{\mu }}}^\star -Y{\hat{{\varvec{e}}}}_j\Vert _{\ell _2}}\left[ I \right. \nonumber \\&\quad \left. - \frac{({{\varvec{\mu }}}^\star -Y{\hat{{\varvec{e}}}}_j)}{\Vert {{\varvec{\mu }}}^\star -Y{\hat{{\varvec{e}}}}_j\Vert _{\ell _2}} \frac{({{\varvec{\mu }}}^\star -Y{\hat{{\varvec{e}}}}_j)^\mathrm{T}}{\Vert {{\varvec{\mu }}}^\star -Y{\hat{{\varvec{e}}}}_j\Vert _{\ell _2}} \right] =: A_j. \end{aligned}$$

For (6.20),

6.24 $$\begin{aligned}&\frac{\partial {{\varvec{G}}}}{\partial {\varvec{d}}_j}({\varvec{d}}_1^\star ,\ldots , {\varvec{d}}_n^\star ,{{\varvec{\mu }}}^\star )\nonumber \\&= \sum _{i=1}^n \frac{I}{1+\tau \Vert {{\varvec{\mu }}}^\star -Y{\hat{{\varvec{e}}}}_i\Vert _{\ell _2}} \big / \sum _{j=1}^n \frac{-\tau }{1+\tau \Vert {{\varvec{\mu }}}^\star -Y{\hat{{\varvec{e}}}}_j\Vert _{\ell _2}} \mathop {\longrightarrow }\limits ^{\tau \rightarrow \infty } 0\nonumber \\ \end{aligned}$$

and

6.25 $$\begin{aligned}&\displaystyle \frac{\partial {{\varvec{G}}}}{\partial {{\varvec{\mu }}}}({\varvec{d}}_1^\star ,\ldots , {\varvec{d}}_n^\star ,{{\varvec{\mu }}}^\star ) = \displaystyle \sum _{i=1}^n \frac{({\varvec{d}}_i^\star -\tau Y{\hat{{\varvec{e}}}}_i)}{[1+\tau \Vert {{\varvec{\mu }}}^\star -\tau Y{\hat{{\varvec{e}}}}_i\Vert _{\ell _2}]^2}\nonumber \\&\qquad \frac{\tau ({{\varvec{\mu }}}^\star -Y{\hat{{\varvec{e}}}}_i)^\mathrm{T}}{\Vert {{\varvec{\mu }}}^\star -Y{\hat{{\varvec{e}}}}_i\Vert _{\ell _2}}\bigg /\sum _{j=1}^n\frac{\tau }{1+\tau \Vert {{\varvec{\mu }}}^\star -Y{\hat{{\varvec{e}}}}_j\Vert _{\ell _2}} \nonumber \\&\qquad + \displaystyle \underbrace{ \sum _{i=1}^n\frac{({\varvec{d}}_i^\star -\tau Y{\hat{{\varvec{e}}}}_i)}{1+\tau \Vert {{\varvec{\mu }}}^\star -Y{\hat{{\varvec{e}}}}_i\Vert _{\ell _2}} }_{=\sum _{i=1}^n \frac{-\tau {{\varvec{\mu }}}^\star }{1+\tau \Vert {{\varvec{\mu }}}^\star -Y{{\varvec{e}}}_i\Vert _{\ell _2}}} \left[ \sum _{j=1}^n \frac{-\tau }{[1+\tau \Vert {{\varvec{\mu }}}^\star -Y{\hat{{\varvec{e}}}}_j\Vert _{\ell _2}]^2}\right. \nonumber \\&\qquad \left. \frac{\tau ({{\varvec{\mu }}}^\star -Y{\hat{{\varvec{e}}}}_j)^\mathrm{T}}{\Vert {{\varvec{\mu }}}^\star -Y{\hat{{\varvec{e}}}}_j\Vert _{\ell _2}} \right] \bigg / \left[ \sum _{k=1}^n \frac{-\tau }{1+\tau \Vert {{\varvec{\mu }}}^\star -Y{\hat{{\varvec{e}}}}_k\Vert _{\ell _2}} \right] ^2\nonumber \\&\quad = \displaystyle \sum _{i=1}^n \frac{[{\varvec{d}}_i^\star +\tau ({{\varvec{\mu }}}^\star - Y{\hat{{\varvec{e}}}}_i)]}{[1+\tau \Vert {{\varvec{\mu }}}^\star -Y{\hat{{\varvec{e}}}}_i\Vert _{\ell _2}]^2}\frac{\tau ({{\varvec{\mu }}}^\star -Y{\hat{{\varvec{e}}}}_i)^\mathrm{T}}{\Vert {{\varvec{\mu }}}^\star -Y{\hat{{\varvec{e}}}}_i\Vert _{\ell _2}}\bigg /\sum _{j=1}^n\nonumber \\&\qquad \frac{\tau }{1+\tau \Vert {{\varvec{\mu }}}^\star -Y{\hat{{\varvec{e}}}}_j\Vert _{\ell _2}}\nonumber \\&\qquad \mathop {\longrightarrow }\limits ^{\tau \rightarrow \infty } \displaystyle \sum _{i=1}^n \frac{({{\varvec{\mu }}}^\star -Y{\hat{{\varvec{e}}}}_i)({{\varvec{\mu }}}^\star -Y{\hat{{\varvec{e}}}}_i)^\mathrm{T}}{\Vert {{\varvec{\mu }}}^\star -Y{\hat{{\varvec{e}}}}_i\Vert _{\ell _2}^3} \bigg /\nonumber \\&\qquad \sum _{j=1}^n \frac{1}{\Vert {{\varvec{\mu }}}^\star -Y{\hat{{\varvec{e}}}}_j\Vert _{\ell _2}} =: B. \end{aligned}$$

Thus, the Jacobian satisfies

6.26 $$\begin{aligned}&\frac{\partial ({{\varvec{F}}}_1,\ldots ,{{\varvec{F}}}_n,{{\varvec{G}}})}{\partial ({\varvec{d}}_1,\ldots ,{\varvec{d}}_n, {{\varvec{\mu }}})}({\varvec{d}}_1^\star ,\ldots ,{\varvec{d}}_n^\star ,{{\varvec{\mu }}}^\star )\nonumber \\&\mathop {\longrightarrow }\limits ^{\tau \rightarrow \infty } \left[ \begin{array}{cccc} 0 &{} \cdots &{} 0 &{} A_1 \\ \vdots &{} \ddots &{} &{} \vdots \\ 0 &{} &{} 0 &{} A_n \\ 0 &{} \cdots &{} 0 &{} B \\ \end{array} \right] =: J. \end{aligned}$$

The matrix $B \in \mathbb {R}^{m\times m}$ is clearly symmetric positive semi-definite, and it will now be shown that its spectrum lies in [0, 1). Suppose there is an ${{\varvec{x}}} \in \mathbb {R}^m$ satisfying

6.27 $$\begin{aligned} {{\varvec{x}}}^\mathrm{T}({{\varvec{\mu }}}^\star -Y{\hat{{\varvec{e}}}}_i) = \Vert {{\varvec{x}}}\Vert _{\ell _2} \Vert {{\varvec{\mu }}}^\star -Y{\hat{{\varvec{e}}}}_i\Vert _{\ell _2}, \quad i=1,\ldots ,n.\nonumber \\ \end{aligned}$$

Then there exists ${\varvec{\alpha }} = \{\alpha _i\}_{i=1}^n$ such that

6.28 $$\begin{aligned} {{\varvec{\mu }}}^\star -Y{\hat{{\varvec{e}}}}_i = \alpha _i {{\varvec{x}}} \quad \Rightarrow \quad Y = {{\varvec{\mu }}}^\star {{\varvec{e}}}^\mathrm{T} - {{\varvec{x}}}{\varvec{\alpha }}^\mathrm{T} \end{aligned}$$

violating the assumption that the columns of *Y* are not collinear. Thus, there can be no ${{\varvec{x}}}$ satisfying (6.27). This result implies strict inequality in the following estimate:

6.29 $$\begin{aligned} {\displaystyle {{\varvec{x}}}^\mathrm{T}B{{\varvec{x}}} }= & {} \displaystyle \sum _{i=1}^n \frac{[{{\varvec{x}}}^\mathrm{T}({{\varvec{\mu }}}^\star -Y{\hat{{\varvec{e}}}}_i)]^2}{\Vert {{\varvec{\mu }}}^\star -Y{\hat{{\varvec{e}}}}_i \Vert _{\ell _2}^3}\Big / \sum _{j=1}^n \frac{1}{\Vert {{\varvec{\mu }}}^\star -Y {\hat{{\varvec{e}}}}_j\Vert _{\ell _2}}\nonumber \\< & {} \displaystyle \sum _{i=1}^n \frac{\Vert {{\varvec{x}}}\Vert _{\ell _2}^2\Vert {{\varvec{\mu }}}^\star -Y{\hat{{\varvec{e}}}}_i \Vert _{\ell _2}^2}{\Vert {{\varvec{\mu }}}^\star -Y{\hat{{\varvec{e}}}}_i\Vert _{\ell _2}^3}\Big / \sum _{j=1}^n\nonumber \\&\quad \frac{1}{\Vert {{\varvec{\mu }}}^\star -Y{\hat{{\varvec{e}}}}_j\Vert _{\ell _2}} = \Vert {{\varvec{x}}}\Vert _{\ell _2}^2, \quad \forall {{\varvec{x}}} \in \mathbb {R}^m. \end{aligned}$$

Thus, the spectral radius of *B* is less than 1. Let a rotation matrix *P* be chosen so that $P^\mathrm{T}BP = \Lambda =\text{ diag }\{\lambda _i\}_{i=1}^m$ where $\lambda _i \in [0,1)$. Then the matrix *J* in (6.26) satisfies

6.30 $$\begin{aligned} \left[ \begin{array}{cccc} I &{} \cdots &{} &{} 0 \\ \vdots &{} \ddots &{} &{} \vdots \\ 0 &{} &{} I &{} 0 \\ 0 &{} \cdots &{} 0 &{} P \\ \end{array} \right] ^\mathrm{T} J \left[ \begin{array}{cccc} I &{} \cdots &{} &{} 0 \\ \vdots &{} \ddots &{} &{} \vdots \\ 0 &{} &{} I &{} 0 \\ 0 &{} \cdots &{} 0 &{} P \\ \end{array} \right] = \left[ \begin{array}{cccc} 0 &{} \cdots &{} 0 &{} A_1P \\ \vdots &{} \ddots &{} &{} \vdots \\ 0 &{} &{} 0 &{} A_nP \\ 0 &{} \cdots &{} 0 &{} \Lambda \\ \end{array} \right] \nonumber \\ \end{aligned}$$

proving that the spectrum of *J* lies in [0, 1). Since the Jacobian in (6.26) is arbitrarily well approximated by *J* when $\tau$ is sufficiently large, the spectral radius of the Jacobian must be less than 1 for $\tau$ sufficiently large. Because of the assumption ${{\varvec{\mu }}}^\star \not \in \{Y{\hat{{\varvec{e}}}}_j\}_{j=1}^n$, the mapping (6.19)–(6.20) is smooth in a neighborhood of the fixed point $\{D^\star ,{{\varvec{\mu }}}^\star \}$, and hence iterates $\{D_l,{{\varvec{\mu }}}_l\}$ converge to the fixed point when started sufficiently close to it.

### *Remark 1*

Computations demonstrate that the iteration (3.6) – (3.7) converges to a minimizer of *M* for all $\tau >0$ and even when the condition ${{\varvec{\mu }}}^\star \not \in \{Y{\hat{{\varvec{e}}}}_j\}_{j=1}^n$ is violated. Furthermore, while the uniqueness of the minimizer is not guaranteed when the non-collinearity condition is violated, the iteration is found to converge to the median shown in Lemma 1 when $\tau$ is sufficiently small.

## Convergence of the Iterative Scheme for $\ell _1$ PCA

The analysis of the scheme (4.10)–(4.11) begins with establishing the existence of a minimizer for $H_k$ in (4.8). Recall the assumption in Sect. 4 that $S_1 = {\mathcal R}(Y_\mathrm{c}) = \mathbb {R}^m$ so that $S_k = {\mathcal R}(Y_k) = {\mathcal R}(V_{k-1})^\perp$, $k=2,\ldots ,m$, as seen in (4.3). Also, define

7.1 $$\begin{aligned} \mathbb {S}_k = \{{\hat{{{\varvec{v}}}}}\in S_k: \Vert {\hat{{{\varvec{v}}}}}\Vert _{\ell _2}=1\}. \end{aligned}$$

### **Lemma 4**

For $H_k$ in (4.8), there exists a minimizer ${\hat{{{\varvec{v}}}}}^\star$ over $S_k$ which satisfies ${\hat{{{\varvec{v}}}}}^\star \in \mathbb {S}_k$. Moreover, $\sum _{j=1}^n \Vert Y_k{\hat{{\varvec{e}}}}_j\Vert _{\ell _2}$ gives an upper bound for the minimum.

### *Proof*

Because of the properties of projections, it follows that $\Vert ({\hat{{{\varvec{v}}}}}{\hat{{{\varvec{v}}}}}^\mathrm{T}-I)Y_k{\hat{{\varvec{e}}}}_j\Vert _{\ell _2} \le \Vert Y_k{\hat{{\varvec{e}}}}_j\Vert _{\ell _2}$ holds $\forall {\hat{{{\varvec{v}}}}} \in \mathbb {S}_k$ and $\forall j$. By (4.8), $H_k({{\varvec{v}}})\le H_k({{\varvec{0}}})$ holds $\forall {{\varvec{v}}} \in S_k$. Thus, $H_k$ is not strictly minimized at ${{\varvec{v}}}={{\varvec{0}}}$. According to (4.8), $H_k$ is constant along rays outside the origin. Therefore, the minimization can as well be carried out over $\mathbb {S}_k$. The claim follows since $\mathbb {S}_k$ is compact and $H_k$ is continuous on $\mathbb {S}_k$. $\square$

### **Lemma 5**

The first-order necessary optimality condition for a minimizer ${\hat{{{\varvec{v}}}}}^\star$ of $H_k$ over $S_k$ satisfying ${\hat{{{\varvec{v}}}}}^\star \in \mathbb {S}_k$ as given by Lemma 4 is that there exists $D^\star \in \mathbb {R}^{m\times n}$ satisfying

7.2 $$\begin{aligned}&\displaystyle ({\hat{{{\varvec{v}}}}}^\star {\hat{{{\varvec{v}}}}}^\star {}^\mathrm{T}-I)Y_k{\hat{{\varvec{e}}}}_j = \Vert ({\hat{{{\varvec{v}}}}}^\star {\hat{{{\varvec{v}}}}}^\star {}^\mathrm{T}-I) Y_k{\hat{{\varvec{e}}}}_j \Vert _{\ell _2} D^\star {\hat{{\varvec{e}}}}_j,\nonumber \\&\quad {\hat{{{\varvec{v}}}}}^\star {}^\mathrm{T}D^\star {\hat{{\varvec{e}}}}_j=0, \quad \Vert D^\star {\hat{{\varvec{e}}}}_j\Vert _{\ell _2} \le 1,\nonumber \\&\qquad j=1,\ldots ,n \nonumber \\&{\displaystyle \sum _{j=1}^n ({\hat{{{\varvec{v}}}}}^\star {}^\mathrm{T}Y_k{\hat{{\varvec{e}}}}_j) D^\star {\hat{{\varvec{e}}}}_j = {{\varvec{0}}}. } \end{aligned}$$

### *Proof*

The necessary optimality condition for a minimizer ${{\varvec{v}}}^\star$ is

7.3 $$\begin{aligned} 0 \in \partial H_k({{\varvec{v}}}) \subset \sum _{j=1}^n \partial \left\| \left( \frac{{{\varvec{v}}}{{\varvec{v}}}^\mathrm{T}}{\Vert {{\varvec{v}}}\Vert _{\ell _2}^2}-I \right) Y_k{\hat{{\varvec{e}}}}_j \right\| _{\ell _2}, \quad {{\varvec{v}}} \ne {{\varvec{0}}},\nonumber \\ \end{aligned}$$

where $\partial$ denotes the Clarke derivative. Thus, there exist ${{\varvec{b}}}_j^\star \in \partial _{{{\varvec{v}}}}\Vert ({{\varvec{v}}}{{\varvec{v}}}^\mathrm{T}/\Vert {{\varvec{v}}}\Vert _{\ell _2}^2-I) Y_k{\hat{{\varvec{e}}}}_j\Vert _{\ell _2}|_{{{\varvec{v}}}={\varvec{v}}^\star }$, $j=1,\ldots ,n$, satisfying

7.4 $$\begin{aligned} \sum _{j=1}^n {{\varvec{b}}}_j^\star = 0. \end{aligned}$$

By the chain rule (see, e.g., [9], Propositions 2.2.7, 2.3.2 and Theorem 2.3.9), the respective Clarke derivatives are given according to

7.5 $$\begin{aligned}&\partial _{{{\varvec{v}}}} \left\| \left( \frac{{{\varvec{v}}}{{\varvec{v}}}^\mathrm{T}}{\Vert {{\varvec{v}}}\Vert _{\ell _2}^2}-I \right) Y_k{\hat{{\varvec{e}}}}_j \right\| _{\ell _2}\nonumber \\&\quad = \left[ \partial _{{{\varvec{v}}}}\left( \frac{{{\varvec{v}}}{{\varvec{v}}}^\mathrm{T}}{\Vert {{\varvec{v}}}\Vert _{\ell _2}^2}Y_k{\hat{{\varvec{e}}}}_j \right) \right] ^\mathrm{T} \underbrace{\partial _{{w}}\Vert {\varvec{w}}-Y_k{\hat{{\varvec{e}}}}_j\Vert _{\ell _2}}_{{w} = {v}{v}^\mathrm{T}Y_k\hat{{e}}_j/\Vert {v}\Vert _{\ell _2}^2} \end{aligned}$$

where

7.6 $$\begin{aligned} \partial _{{{\varvec{v}}}}\left( \frac{{{\varvec{v}}}{{\varvec{v}}}^\mathrm{T}}{\Vert {{\varvec{v}}}\Vert _{\ell _2}^2}Y_k{\hat{{\varvec{e}}}}_j \right) = \frac{{{\varvec{v}}}^\mathrm{T}Y_k{\hat{{\varvec{e}}}}_j}{\Vert {{\varvec{v}}}\Vert _{\ell _2}^2}I + \frac{{{\varvec{v}}}{\hat{{\varvec{e}}}}_j^\mathrm{T}Y_k^\mathrm{T}}{\Vert {{\varvec{v}}}\Vert _{\ell _2}^2} - \frac{2{{\varvec{v}}}{{\varvec{v}}}^\mathrm{T}}{\Vert {{\varvec{v}}}\Vert _{\ell _2}^4} {{\varvec{v}}}^\mathrm{T}Y_k{\hat{{\varvec{e}}}}_j\nonumber \\ \end{aligned}$$

and

7.7 $$\begin{aligned} \partial _{{{\varvec{w}}}} \Vert {\varvec{w}}-Y_k{\hat{{\varvec{e}}}}_j\Vert _{\ell _2} = \left\{ \begin{array}{l@{\quad }l} {\displaystyle \frac{{\varvec{w}}-Y_k{\hat{{\varvec{e}}}}_j}{\Vert {\varvec{w}}-Y_k{\hat{{\varvec{e}}}}_j\Vert _{\ell _2}}, } &{} {\displaystyle {\varvec{w}}\ne Y_k{\hat{{\varvec{e}}}}_j} \\ B(0,1), &{} {\varvec{w}}= Y_k{\hat{{\varvec{e}}}}_j \end{array} \right. \end{aligned}$$

with the unit ball *B*(0, 1). Let ${\varvec{c}}_j^\star$ be chosen so that

7.8 $$\begin{aligned} {\varvec{c}}_j^\star \in \left\{ \begin{array}{l@{\quad }l} {\displaystyle \frac{({\hat{{{\varvec{v}}}}}^\star {\hat{{{\varvec{v}}}}}^\star {}^\mathrm{T}-I) Y_k{\hat{{\varvec{e}}}}_j}{\Vert ({\hat{{{\varvec{v}}}}}^\star {\hat{{{\varvec{v}}}}}^\star {}^\mathrm{T}-I) Y_k{\hat{{\varvec{e}}}}_j\Vert _{\ell _2}}, } &{} Y_k{\hat{{\varvec{e}}}}_j \ne {\hat{{{\varvec{v}}}}}^\star {\hat{{{\varvec{v}}}}}^\star {}^\mathrm{T} Y_k{\hat{{\varvec{e}}}}_j \\ B(0,1), &{} Y_k{\hat{{\varvec{e}}}}_j = {\hat{{{\varvec{v}}}}}^\star {\hat{{{\varvec{v}}}}}^\star {}^\mathrm{T}Y_k{\hat{{\varvec{e}}}}_j \end{array} \right. \end{aligned}$$

and

7.9 $$\begin{aligned} {{\varvec{b}}}_j^\star = [{\hat{{{\varvec{v}}}}}^\star {}^\mathrm{T}Y_k{\hat{{\varvec{e}}}}_jI + {\hat{{{\varvec{v}}}}}^\star {\hat{{\varvec{e}}}}_j^\mathrm{T}Y_k^\mathrm{T} - 2{\hat{{{\varvec{v}}}}}^\star {\hat{{{\varvec{v}}}}}^\star {}^\mathrm{T} {\hat{{{\varvec{v}}}}}^\star {}^\mathrm{T}Y_k{\hat{{\varvec{e}}}}_j]{\varvec{c}}_j^\star . \end{aligned}$$

According to (7.8),

7.10 $$\begin{aligned} \Vert ({\hat{{{\varvec{v}}}}}^\star {\hat{{{\varvec{v}}}}}^\star {}^\mathrm{T}-I) Y_k{\hat{{\varvec{e}}}}_j\Vert _{\ell _2}{\varvec{c}}_j^\star = ({\hat{{{\varvec{v}}}}}^\star {\hat{{{\varvec{v}}}}}^\star {}^\mathrm{T}-I) Y_k{\hat{{\varvec{e}}}}_j, \quad j=1,\ldots ,n.\nonumber \\ \end{aligned}$$

With (7.9), it follows for $Y_k{\hat{{\varvec{e}}}}_j \ne {\hat{{{\varvec{v}}}}}^\star {\hat{{{\varvec{v}}}}}^\star {}^\mathrm{T}Y_k{\hat{{\varvec{e}}}}_j$,

7.11 $$\begin{aligned} {{\varvec{b}}}_j^\star= & {} \displaystyle \left[ {\hat{{{\varvec{v}}}}}^\star {}^\mathrm{T}Y_k{\hat{{\varvec{e}}}}_jI + Y_k{\hat{{\varvec{e}}}}_j{\hat{{{\varvec{v}}}}}^\star {}^\mathrm{T} - 2{\hat{{{\varvec{v}}}}}^\star {}^\mathrm{T}Y_k{\hat{{\varvec{e}}}}_j {\hat{{{\varvec{v}}}}}^\star {\hat{{{\varvec{v}}}}}^\star {}^\mathrm{T} \right] \nonumber \\&\quad \frac{({\hat{{{\varvec{v}}}}}^\star {\hat{{{\varvec{v}}}}}^\star {}^\mathrm{T}-I) Y_k{\hat{{\varvec{e}}}}_j}{\Vert ({\hat{{{\varvec{v}}}}}^\star {\hat{{{\varvec{v}}}}}^\star {}^\mathrm{T}-I) Y_k{\hat{{\varvec{e}}}}_j\Vert _{\ell _2}}\nonumber \\= & {} \displaystyle ({\hat{{{\varvec{v}}}}}^\star {}^\mathrm{T}Y_k{\hat{{\varvec{e}}}}_j)\frac{({\hat{{{\varvec{v}}}}}^\star {\hat{{{\varvec{v}}}}}^\star {}^\mathrm{T}-I) Y_k{\hat{{\varvec{e}}}}_j}{\Vert ({\hat{{{\varvec{v}}}}}^\star {\hat{{{\varvec{v}}}}}^\star {}^\mathrm{T}-I) Y_k{\hat{{\varvec{e}}}}_j\Vert _{\ell _2}}\nonumber \\= & {} ({\hat{{{\varvec{v}}}}}^\star {}^\mathrm{T}Y_k{\hat{{\varvec{e}}}}_j) (I-{\hat{{{\varvec{v}}}}}^\star {\hat{{{\varvec{v}}}}}^\star {}^\mathrm{T})\frac{({\hat{{{\varvec{v}}}}}^\star {\hat{{{\varvec{v}}}}}^\star {}^\mathrm{T}-I) Y_k{\hat{{\varvec{e}}}}_j}{\Vert ({\hat{{{\varvec{v}}}}}^\star {\hat{{{\varvec{v}}}}}^\star {}^\mathrm{T}-I) Y_k{\hat{{\varvec{e}}}}_j\Vert _{\ell _2}}\nonumber \\= & {} ({\hat{{{\varvec{v}}}}}^\star {}^\mathrm{T}Y_k{\hat{{\varvec{e}}}}_j) (I-{\hat{{{\varvec{v}}}}}^\star {\hat{{{\varvec{v}}}}}^\star {}^\mathrm{T}){\varvec{c}}_j^\star \end{aligned}$$

and for $$Y_k{\hat{{\varvec{e}}}}_j = {\hat{{{\varvec{v}}}}}^\star {\hat{{{\varvec{v}}}}}^\star {}^\mathrm{T}Y_k{\hat{{\varvec{e}}}}_j$$,

7.12 $$\begin{aligned} {{\varvec{b}}}_j^\star= & {} [{\hat{{{\varvec{v}}}}}^\star {}^\mathrm{T}Y_k{\hat{{\varvec{e}}}}_jI + {\hat{{{\varvec{v}}}}}^\star {\hat{{\varvec{e}}}}_j^\mathrm{T}Y_k^\mathrm{T} - 2{\hat{{{\varvec{v}}}}}^\star {\hat{{{\varvec{v}}}}}^\star {}^\mathrm{T} {\hat{{{\varvec{v}}}}}^\star {}^\mathrm{T}Y_k{\hat{{\varvec{e}}}}_j]{\varvec{c}}_j^\star \nonumber \\= & {} ({\hat{{{\varvec{v}}}}}^\star {}^\mathrm{T}Y_k{\hat{{\varvec{e}}}}_j) (I-{\hat{{{\varvec{v}}}}}^\star {\hat{{{\varvec{v}}}}}^\star {}^\mathrm{T}){\varvec{c}}_j^\star . \end{aligned}$$

Define

7.13 $$\begin{aligned} {\varvec{d}}_j^\star = (I-{\hat{{{\varvec{v}}}}}^\star {\hat{{{\varvec{v}}}}}^\star {}^\mathrm{T}){\varvec{c}}_j^\star . \end{aligned}$$

Combining (7.4), (7.11), (7.12), and (7.13) gives

7.14 $$\begin{aligned} 0 = \sum _{j=1}^n {{\varvec{b}}}_j^\star = \sum _{j=1}^n ({\hat{{{\varvec{v}}}}}^\star {}^\mathrm{T}Y_k{\hat{{\varvec{e}}}}_j) {\varvec{d}}_j^\star . \end{aligned}$$

The claim (7.2) follows with $D^\star = \{{\varvec{d}}_1^\star ,\ldots ,{\varvec{d}}_n^\star \}$. $\square$

Turning to the iteration (4.10)–(4.11), we observe that if convergence to a fixed point $\{D^\star ,{\hat{{{\varvec{v}}}}}^\star \}$ with $\Vert {\hat{{{\varvec{v}}}}}^\star \Vert _{\ell _2}=1$ can be guaranteed, then from (4.10) we have that ${\hat{{{\varvec{v}}}}}^\star {}^\mathrm{T}D_j^\star {\hat{{\varvec{e}}}}_j = 0$ and

7.15 $$\begin{aligned}&\Vert ({\hat{{{\varvec{v}}}}}^\star {\hat{{{\varvec{v}}}}}^\star {}^\mathrm{T}-I) Y_k{\hat{{\varvec{e}}}}_j\Vert _{\ell _2} D^\star {\hat{{\varvec{e}}}}_j \nonumber \\&\quad =({\hat{{{\varvec{v}}}}}^\star {\hat{{{\varvec{v}}}}}^\star {}^\mathrm{T}-I) Y_k{\hat{{\varvec{e}}}}_j, \quad j=1,\ldots ,n. \end{aligned}$$

According to (4.11), the fixed point ${\hat{{{\varvec{v}}}}}^\star$ satisfies

7.16 $$\begin{aligned} {\hat{{{\varvec{v}}}}}^\star \Vert {{\varvec{v}}}^\star \Vert _{\ell _2} = {{\varvec{v}}}^\star = {\hat{{{\varvec{v}}}}}^\star -\rho \sum _{j=1}^n \frac{({\hat{{{\varvec{v}}}}}^\star {}^\mathrm{T}Y_k{\hat{{\varvec{e}}}}_j) (\tau Y_k{\hat{{\varvec{e}}}}_j-D_l^\star {\hat{{\varvec{e}}}}_j)}{1 + \tau \Vert ({\hat{{{\varvec{v}}}}}^\star {\hat{{{\varvec{v}}}}}^\star {}^\mathrm{T}-I) Y_k{\hat{{\varvec{e}}}}_j\Vert _{\ell _2}},\nonumber \\ \end{aligned}$$

where ${{\varvec{v}}}^\star$ is defined by the right side of (7.16). Applying $({\hat{{{\varvec{v}}}}}^\star {\hat{{{\varvec{v}}}}}^\star {}^\mathrm{T}-I)$ to both sides of (7.16) gives

7.17 $$\begin{aligned} 0= & {} ({\hat{{{\varvec{v}}}}}^\star {\hat{{{\varvec{v}}}}}^\star {}^\mathrm{T}-I) \sum _{j=1}^n \frac{({\hat{{{\varvec{v}}}}}^\star {}^\mathrm{T}Y_k{\hat{{\varvec{e}}}}_j) (\tau Y_k{\hat{{\varvec{e}}}}_j-D_l^\star {\hat{{\varvec{e}}}}_j)}{1 + \tau \Vert ({\hat{{{\varvec{v}}}}}^\star {\hat{{{\varvec{v}}}}}^\star {}^\mathrm{T}-I) Y_k{\hat{{\varvec{e}}}}_j\Vert _{\ell _2}}\nonumber \\= & {} \sum _{j=1}^n ({\hat{{{\varvec{v}}}}}^\star {}^\mathrm{T}Y_k{\hat{{\varvec{e}}}}_j) \frac{D_l^\star {\hat{{\varvec{e}}}}_j+ \tau ({\hat{{{\varvec{v}}}}}^\star {\hat{{{\varvec{v}}}}}^\star {}^\mathrm{T}-I)Y_k{\hat{{\varvec{e}}}}_j}{1 + \tau \Vert ({\hat{{{\varvec{v}}}}}^\star {\hat{{{\varvec{v}}}}}^\star {}^\mathrm{T}-I) Y_k{\hat{{\varvec{e}}}}_j\Vert _{\ell _2}}. \end{aligned}$$

Combining (7.15) and (7.17) gives

7.18 $$\begin{aligned} 0= & {} \sum _{j=1}^n ({\hat{{{\varvec{v}}}}}^\star {}^\mathrm{T}Y_k{\hat{{\varvec{e}}}}_j) \frac{D^\star {\hat{{\varvec{e}}}}_j+\tau \Vert ({\hat{{{\varvec{v}}}}}^\star {\hat{{{\varvec{v}}}}}^\star {}^\mathrm{T}-I) Y_k{\hat{{\varvec{e}}}}_j\Vert _{\ell _2} D^\star {\hat{{\varvec{e}}}}_j}{1+\tau \Vert ({\hat{{{\varvec{v}}}}}^\star {\hat{{{\varvec{v}}}}}^\star {}^\mathrm{T}-I)Y_k{\hat{{\varvec{e}}}}_j\Vert _{\ell _2}}\nonumber \\= & {} \sum _{j=1}^n ({\hat{{{\varvec{v}}}}}^\star {}^\mathrm{T}Y_k{\hat{{\varvec{e}}}}_j)D^\star {\hat{{\varvec{e}}}}_j. \end{aligned}$$

Moreover, if $\Vert D_0{\hat{{\varvec{e}}}}_j\Vert _{\ell _2} \le 1, j=1,\ldots ,n,$ then the iterates $\{D_l\}$ also satisfy this bound, and hence $\Vert D^\star {\hat{{\varvec{e}}}}_j\Vert _{\ell _2} \le 1, j=1,\ldots ,n$. Thus, $\{D^\star ,{\hat{{{\varvec{v}}}}}^\star \}$ must satisfy the necessary optimality condition (7.2). The following theorem provides sufficient conditions under which convergence of our algorithm to a fixed point can be guaranteed.

### **Theorem 2**

Let $\{D^\star ,{\hat{{{\varvec{v}}}}}^\star \}$ satisfy (7.2) with ${\hat{{{\varvec{v}}}}}^\star \in \mathbb {S}_k$ and suppose

7.19 $$\begin{aligned} {\hat{{{\varvec{v}}}}}^\star {}^\mathrm{T}Y_k{\hat{{\varvec{e}}}}_j \ne 0, \quad ({\hat{{{\varvec{v}}}}}^\star {\hat{{{\varvec{v}}}}}^\star {}^\mathrm{T}-I)Y_k{\hat{{\varvec{e}}}}_j\ne {{\varvec{0}}}, \quad j=1,\ldots ,n.\nonumber \\ \end{aligned}$$

Then $\{D^\star ,{\hat{{{\varvec{v}}}}}^\star \}$ is a fixed point of the iteration (4.10)–(4.11), and for $\tau$ sufficiently large and for $\rho$ sufficiently small, the iterates $\{D_l,{\hat{{{\varvec{v}}}}}_l\}$ converge to this fixed point when $\{D_0,{\hat{{{\varvec{v}}}}}_0\}$ starts the iteration close enough to $\{D^\star ,{\hat{{{\varvec{v}}}}}^\star \}$.

### *Proof*

It will first be shown that $\{D^\star ,{\hat{{{\varvec{v}}}}}^\star \}$ is a fixed point for the iteration (4.10)–(4.11), which is locally asymptotically stable for $\tau$ sufficiently large and for $\rho$ sufficiently small. Using (7.2) and substituting $D_l = D^\star$ and $\hat{\mathbf{v}}_l = {\hat{{{\varvec{v}}}}}^\star$ on the right side of (4.10),

7.20 $$\begin{aligned} D_{l+1}{\hat{{\varvec{e}}}}_j= & {} \frac{({\hat{{{\varvec{v}}}}}^\star {\hat{{{\varvec{v}}}}}^\star {}^\mathrm{T}-I)(\tau Y_k{\hat{{\varvec{e}}}}_j- D^\star {\hat{{\varvec{e}}}}_j)}{1+\tau \Vert ({\hat{{{\varvec{v}}}}}^\star {\hat{{{\varvec{v}}}}}^\star {}^\mathrm{T}-I) Y_k{\hat{{\varvec{e}}}}_j\Vert _{\ell _2}}\nonumber \\= & {} \frac{\tau \Vert ({\hat{{{\varvec{v}}}}}^\star {\hat{{{\varvec{v}}}}}^\star {}^\mathrm{T}-I) Y_k{\hat{{\varvec{e}}}}_j\Vert _{\ell _2}D^\star {\hat{{\varvec{e}}}}_j+D^\star {\hat{{\varvec{e}}}}_j}{1+\tau \Vert ({\hat{{{\varvec{v}}}}}^\star {\hat{{{\varvec{v}}}}}^\star {}^\mathrm{T}-I) Y_k{\hat{{\varvec{e}}}}_j\Vert _{\ell _2}}=D^\star {\hat{{\varvec{e}}}}_j\nonumber \\ \end{aligned}$$

gives $D_{l+1} = D^\star$. Also by (7.2),

7.21 $$\begin{aligned} 0= & {} {\displaystyle \sum _{j=1}^n ({\hat{{{\varvec{v}}}}}^\star {}^\mathrm{T}Y_k{\hat{{\varvec{e}}}}_j)D^\star {\hat{{\varvec{e}}}}_j \frac{1+\tau \Vert ({\hat{{{\varvec{v}}}}}^\star {\hat{{{\varvec{v}}}}}^\star {}^\mathrm{T}-I)Y_k{\hat{{\varvec{e}}}}_j\Vert _{\ell _2}}{1+\tau \Vert ({\hat{{{\varvec{v}}}}}^\star {\hat{{{\varvec{v}}}}}^\star {}^\mathrm{T}-I)Y_k{\hat{{\varvec{e}}}}_j\Vert _{\ell _2}} } \nonumber \\= & {} {\displaystyle \sum _{j=1}^n ({\hat{{{\varvec{v}}}}}^\star {}^\mathrm{T}Y_k{\hat{{\varvec{e}}}}_j) \frac{D^\star {\hat{{\varvec{e}}}}_j+\tau ({\hat{{{\varvec{v}}}}}^\star {\hat{{{\varvec{v}}}}}^\star {}^\mathrm{T}-I)Y_k{\hat{{\varvec{e}}}}_j}{1+\tau \Vert ({\hat{{{\varvec{v}}}}}^\star {\hat{{{\varvec{v}}}}}^\star {}^\mathrm{T}-I)Y_k{\hat{{\varvec{e}}}}_j\Vert _{\ell _2}} } \nonumber \\= & {} {\displaystyle ({\hat{{{\varvec{v}}}}}^\star {\hat{{{\varvec{v}}}}}^\star {}^\mathrm{T}-I) \sum _{j=1}^n ({\hat{{{\varvec{v}}}}}^\star {}^\mathrm{T}Y_k{\hat{{\varvec{e}}}}_j) \frac{\tau Y_k{\hat{{\varvec{e}}}}_j-D^\star {\hat{{\varvec{e}}}}_j}{1+\tau \Vert ({\hat{{{\varvec{v}}}}}^\star {\hat{{{\varvec{v}}}}}^\star {}^\mathrm{T}-I)Y_k{\hat{{\varvec{e}}}}_j\Vert _{\ell _2}} }\nonumber \\ \end{aligned}$$

and hence

7.22 $$\begin{aligned} {{\varvec{v}}}^\star= & {} {\hat{{{\varvec{v}}}}}^\star - \rho \sum _{j=1}^n \frac{({\hat{{{\varvec{v}}}}}^\star {}^\mathrm{T}Y_l{\hat{{\varvec{e}}}}_j) (\tau Y_k{\hat{{\varvec{e}}}}_j-D^\star {\hat{{\varvec{e}}}}_j)}{1+\tau \Vert ({\hat{{{\varvec{v}}}}}^\star {\hat{{{\varvec{v}}}}}^\star {}^\mathrm{T}-I)Y_k{\hat{{\varvec{e}}}}_j\Vert _{\ell _2}}\nonumber \\= & {} {\hat{{{\varvec{v}}}}}^\star \left[ 1 - \rho {\hat{{{\varvec{v}}}}}^\star {}^\mathrm{T} \sum _{j=1}^n \frac{({\hat{{{\varvec{v}}}}}^\star {}^\mathrm{T}Y_l{\hat{{\varvec{e}}}}_j) (\tau Y_k{\hat{{\varvec{e}}}}_j-D^\star {\hat{{\varvec{e}}}}_j)}{1+\tau \Vert ({\hat{{{\varvec{v}}}}}^\star {\hat{{{\varvec{v}}}}}^\star {}^\mathrm{T}-I)Y_k{\hat{{\varvec{e}}}}_j\Vert _{\ell _2}} \right] \nonumber \\ \end{aligned}$$

satisfies ${{\varvec{v}}}^\star = {\hat{{{\varvec{v}}}}}^\star \Vert {{\varvec{v}}}^\star \Vert _{\ell _2}$. Thus, setting $D_l = D^\star$ and $\hat{\mathbf{v}}_l = {\hat{{{\varvec{v}}}}}^\star$ on the right side of (4.11) gives $\hat{\mathbf{v}}_{l+1} = {\hat{{{\varvec{v}}}}}^\star$. Therefore, $\{D^\star ,{\hat{{{\varvec{v}}}}}^\star \}$ is a fixed point of the iteration (4.10)–(4.11).

To establish the stability of the fixed point, define

7.23 $$\begin{aligned} {{\varvec{F}}}_j({\varvec{d}}_1,\ldots ,{\varvec{d}}_n,{{\varvec{v}}})= & {} \frac{({{\varvec{v}}}{{\varvec{v}}}^\mathrm{T}-I)(\tau Y_k{\hat{{\varvec{e}}}}_j-{\varvec{d}}_j)}{1+\tau \Vert ({{\varvec{v}}}{{\varvec{v}}}^\mathrm{T}-I)Y_k{\hat{{\varvec{e}}}}_j\Vert _{\ell _2}}, \nonumber \\&j=1,\ldots ,n \end{aligned}$$

and

7.24 $$\begin{aligned}&\displaystyle {{\varvec{G}}}({\varvec{d}}_1,\ldots ,{\varvec{d}}_n,{{\varvec{v}}}) = \frac{{{\varvec{g}}}({\varvec{d}}_1,\ldots ,{\varvec{d}}_n,{{\varvec{v}}})}{\Vert {{\varvec{g}}} ({\varvec{d}}_1,\ldots ,{\varvec{d}}_n,{{\varvec{v}}})\Vert _{\ell _2}}\nonumber \\&\displaystyle {{\varvec{g}}}({\varvec{d}}_1,\ldots ,{\varvec{d}}_n,{{\varvec{v}}}) = {{\varvec{v}}}\nonumber \\&\quad - \rho \sum _{j=1}^n \frac{({{\varvec{v}}}^\mathrm{T}Y_k{\hat{{\varvec{e}}}}_j) (\tau Y_k{\hat{{\varvec{e}}}}_j-{\varvec{d}}_j)/\Vert {{\varvec{v}}}\Vert _{\ell _2}}{1 + \tau \Vert ({{\varvec{v}}}{{\varvec{v}}}^\mathrm{T}-I) Y_k{\hat{{\varvec{e}}}}_j\Vert _{\ell _2}} \end{aligned}$$

so that (4.10)–(4.11) is given by

7.25 $$\begin{aligned} D_{l+1}{\hat{{\varvec{e}}}}_j= & {} {{\varvec{F}}}_j(D_l{\hat{{\varvec{e}}}}_1,\ldots ,D_l{\hat{{\varvec{e}}}}_n,\hat{\mathbf{v}}_l), \quad j=1,\ldots ,n, \quad \hat{\mathbf{v}}_{l+1} \nonumber \\= & {} {{\varvec{G}}}(D_{l+1}{\hat{{\varvec{e}}}}_1,\ldots ,D_{l+1}{\hat{{\varvec{e}}}}_n, \hat{\mathbf{v}}_l). \end{aligned}$$

The claimed stability will follow once it is shown that the Jacobian of this mapping from the $(n+1)$-fold Cartesian product $(S_k)^{n+1}$ into $(S_k)^{n+1}$ evaluated at $\{D^\star ,{\hat{{{\varvec{v}}}}}^\star \} = \{{\varvec{d}}_1^\star ,\ldots ,{\varvec{d}}_n^\star ,{\hat{{{\varvec{v}}}}}^\star \}$ has only eigenvalues with magnitude less than 1 when $\tau$ is sufficiently large and $\rho$ is sufficiently small. For (7.23),

7.26 $$\begin{aligned}&\frac{\partial {{\varvec{F}}}_j}{\partial {\varvec{d}}_i}({\varvec{d}}_1^\star ,\ldots , {\varvec{d}}_n^\star ,{\hat{{{\varvec{v}}}}}^\star )\nonumber \\&= \frac{I-{\hat{{{\varvec{v}}}}}^\star {\hat{{{\varvec{v}}}}}^\star {}^\mathrm{T}}{1+\tau \Vert ({\hat{{{\varvec{v}}}}}^\star {\hat{{{\varvec{v}}}}}^\star {}^\mathrm{T}-I) Y_k{\hat{{\varvec{e}}}}_j\Vert _{\ell _2}} \mathop {\longrightarrow }\limits ^{\tau \rightarrow \infty } 0 \end{aligned}$$

and

7.27 $$\begin{aligned}&\displaystyle \frac{\partial {{\varvec{F}}}_j}{\partial {{\varvec{v}}}}({\varvec{d}}_1^\star ,\ldots , {\varvec{d}}_n^\star ,{{\varvec{v}}})\nonumber \\&\quad =\left\{ \frac{{{\varvec{v}}}^\mathrm{T}(\tau Y_k{\hat{{\varvec{e}}}}_j-{\varvec{d}}_j^\star ) I + {{\varvec{v}}}(\tau Y_k{\hat{{\varvec{e}}}}_j-{\varvec{d}}_j^\star )^\mathrm{T}}{1+\tau \Vert ({{\varvec{v}}} {{\varvec{v}}}^\mathrm{T}-I)Y_k{\hat{{\varvec{e}}}}_j\Vert _{\ell _2}} \right. \nonumber \\&\qquad \displaystyle \left. - \frac{({{\varvec{v}}} {{\varvec{v}}}^\mathrm{T}-I)(\tau Y_k{\hat{{\varvec{e}}}}_j- {\varvec{d}}_j)}{[1+\tau \Vert ({{\varvec{v}}} {{\varvec{v}}}^\mathrm{T}-I)Y_k{\hat{{\varvec{e}}}}_j\Vert _{\ell _2}]^2}\right. \nonumber \\&\left. \qquad \left[ \left( {{\varvec{v}}}^\mathrm{T}Y_k{\hat{{\varvec{e}}}}_j I + {{\varvec{v}}} {\hat{{\varvec{e}}}}_j^\mathrm{T}Y_k^\mathrm{T} \right) ^\mathrm{T} \frac{\tau ({{\varvec{v}}} {{\varvec{v}}}^\mathrm{T}-I)Y_k{\hat{{\varvec{e}}}}_j}{\Vert ({{\varvec{v}}} {{\varvec{v}}}^\mathrm{T}-I)Y_k{\hat{{\varvec{e}}}}_j\Vert _{\ell _2}} \right] ^\mathrm{T} \right\} _{{{\varvec{v}}}=\hat{{{\varvec{v}}}}^\star } \nonumber \\&\qquad \displaystyle \mathop {\longrightarrow }\limits ^{\tau \rightarrow \infty } \frac{{\hat{{{\varvec{v}}}}}^\star {}^\mathrm{T}Y_k{\hat{{\varvec{e}}}}_j I + {\hat{{{\varvec{v}}}}}^\star {\hat{{\varvec{e}}}}_j^\mathrm{T}Y_k^\mathrm{T}}{\Vert ({\hat{{{\varvec{v}}}}}^\star {\hat{{{\varvec{v}}}}}^\star {}^\mathrm{T}-I)Y_k{\hat{{\varvec{e}}}}_j\Vert _{\ell _2}}\nonumber \\&\qquad - ({\hat{{{\varvec{v}}}}}^\star {}^\mathrm{T}Y_k{\hat{{\varvec{e}}}}_j) \frac{({\hat{{{\varvec{v}}}}}^\star {\hat{{{\varvec{v}}}}}^\star {}^\mathrm{T}-I)Y_k{\hat{{\varvec{e}}}}_j {\hat{{\varvec{e}}}}_j^\mathrm{T}Y_k^\mathrm{T} ({\hat{{{\varvec{v}}}}}^\star {\hat{{{\varvec{v}}}}}^\star {}^\mathrm{T}-I) }{\Vert ({\hat{{{\varvec{v}}}}}^\star {\hat{{{\varvec{v}}}}}^\star {}^\mathrm{T}-I)Y_k{\hat{{\varvec{e}}}}_j\Vert _{\ell _2}^3}:=A_j.\nonumber \\ \end{aligned}$$

For (7.24),

7.28 $$\begin{aligned}&\frac{\partial {{\varvec{g}}}}{\partial {\varvec{d}}_i}({\varvec{d}}_1^\star ,\ldots , {\varvec{d}}_n^\star ,{\hat{{{\varvec{v}}}}}^\star )\nonumber \\&\quad = \rho \frac{({\hat{{{\varvec{v}}}}}^\star {}^\mathrm{T}Y_k{\hat{{\varvec{e}}}}_i)I/\Vert {\hat{{{\varvec{v}}}}}^\star \Vert _{\ell _2}}{1+\tau \Vert ({\hat{{{\varvec{v}}}}}^\star {\hat{{{\varvec{v}}}}}^\star {}^\mathrm{T}-I)Y_k{\hat{{\varvec{e}}}}_i\Vert _{\ell _2}} \mathop {\longrightarrow }\limits ^{\tau \rightarrow \infty } 0 \end{aligned}$$

and with $$\hat{{{\varvec{g}}}}^\star = {{\varvec{g}}}({\varvec{d}}_1^\star ,\ldots , {\varvec{d}}_n^\star ,{\hat{{{\varvec{v}}}}}^\star ) = {\hat{{{\varvec{v}}}}}^\star $$,

7.29 $$\begin{aligned}&\displaystyle \frac{\partial {{\varvec{G}}}}{\partial {\varvec{d}}_i}({\varvec{d}}_1^\star ,\ldots , {\varvec{d}}_n^\star ,{\hat{{{\varvec{v}}}}}^\star )\nonumber \\&\quad = \frac{1}{\Vert \hat{{{\varvec{g}}}}^\star \Vert _{\ell _2}}\left( I-\frac{\hat{{{\varvec{g}}}}^\star \hat{{{\varvec{g}}}}^\star {}^\mathrm{T}}{\Vert \hat{{{\varvec{g}}}}^\star \Vert _{\ell _2}} \right) \frac{\partial {{\varvec{g}}}}{\partial {\varvec{d}}_i}({\varvec{d}}_1^\star ,\ldots , {\varvec{d}}_n^\star ,{\hat{{{\varvec{v}}}}}^\star )\nonumber \\&\displaystyle = (I-{\hat{{{\varvec{v}}}}}^\star {\hat{{{\varvec{v}}}}}^\star {}^\mathrm{T}) \frac{\partial {{\varvec{g}}}}{\partial {\varvec{d}}_i}({\varvec{d}}_1^\star ,\ldots , {\varvec{d}}_n^\star ,{\hat{{{\varvec{v}}}}}^\star ) \mathop {\longrightarrow }\limits ^{\tau \rightarrow \infty } 0. \end{aligned}$$

Also

7.30 $$\begin{aligned}&\displaystyle \frac{\partial {{\varvec{g}}}}{\partial {{\varvec{v}}}}({\varvec{d}}_1^\star ,\ldots , {\varvec{d}}_n^\star ,{{\varvec{v}}})\nonumber \\&\quad = \left\{ I- \rho \sum _{j=1}^n \frac{(\tau Y_k{\hat{{\varvec{e}}}}_j-{\varvec{d}}_j^\star ) {\hat{{\varvec{e}}}}_j^\mathrm{T}Y_k^\mathrm{T}}{1+\tau \Vert ({{\varvec{v}}}{{\varvec{v}}}^\mathrm{T}-I)\Vert _{\ell _2}} \left( I-\frac{{{\varvec{v}}} {{\varvec{v}}}^\mathrm{T}}{\Vert {{\varvec{v}}}\Vert _{\ell _2}^2} \right) \frac{1}{\Vert {{\varvec{v}}}\Vert _{\ell _2}} \right. \nonumber \\&\qquad \displaystyle \left. +\,\rho \sum _{j=1}^n \frac{({{\varvec{v}}}^\mathrm{T}Y_k{\hat{{\varvec{e}}}}_j) (\tau Y_k{\hat{{\varvec{e}}}}_j-{\varvec{d}}_j)/\Vert {{\varvec{v}}}\Vert _{\ell _2}}{[1 + \tau \Vert ({{\varvec{v}}}{{\varvec{v}}}^\mathrm{T}-I) Y_k{\hat{{\varvec{e}}}}_j\Vert _{\ell _2}]^2}\right. \nonumber \\&\qquad \left. \left[ \left( {{\varvec{v}}}^\mathrm{T}Y_k{\hat{{\varvec{e}}}}_j I + {{\varvec{v}}}{\hat{{\varvec{e}}}}_j^\mathrm{T}Y_k^\mathrm{T} \right) ^\mathrm{T} \frac{\tau ({{\varvec{v}}} {{\varvec{v}}}^\mathrm{T}-I)Y_k{\hat{{\varvec{e}}}}_j}{\Vert ({{\varvec{v}}} {{\varvec{v}}}^\mathrm{T}-I)Y_k{\hat{{\varvec{e}}}}_j\Vert _{\ell _2}} \right] ^\mathrm{T}\right\} _{{{\varvec{v}}}=\hat{{{\varvec{v}}}}^\star }\nonumber \\&\qquad \displaystyle =I-\rho \sum _{j=1}^n \frac{(\tau Y_k{\hat{{\varvec{e}}}}_j-{\varvec{d}}_j^\star ) {\hat{{\varvec{e}}}}_j^\mathrm{T}Y_k^\mathrm{T}}{1+\tau \Vert (\hat{{\hat{{{\varvec{v}}}}}}^\star {\hat{{{\varvec{v}}}}}^\star {}^\mathrm{T}-I)Y_k{\hat{{\varvec{e}}}}_j\Vert _{\ell _2}}\nonumber \\&\qquad \left[ 1 + \frac{\tau ({\hat{{{\varvec{v}}}}}^\mathrm{T}Y_k{\hat{{\varvec{e}}}}_j)^2}{[1 + \tau \Vert ({\hat{{{\varvec{v}}}}}{\hat{{{\varvec{v}}}}}^\mathrm{T}-I) Y_k{\hat{{\varvec{e}}}}_j\Vert _{\ell _2}] \Vert ({\hat{{{\varvec{v}}}}}{\hat{{{\varvec{v}}}}}^\mathrm{T}-I)Y_k{\hat{{\varvec{e}}}}_j\Vert _{\ell _2}} \right] \nonumber \\&\qquad \times \displaystyle (I-{\hat{{{\varvec{v}}}}}^\star {\hat{{{\varvec{v}}}}}^\star {}^\mathrm{T}) \nonumber \\&\qquad \displaystyle \mathop {\longrightarrow }\limits ^{\tau \rightarrow \infty } I-\rho \left[ \sum _{j=1}^n ({\hat{{{\varvec{v}}}}}^\star {}^\mathrm{T}Y_k{\hat{{\varvec{e}}}}_j)^2\right. \nonumber \\&\qquad \left. \frac{Y_k{\hat{{\varvec{e}}}}_j {\hat{{\varvec{e}}}}_j^\mathrm{T}Y_k^\mathrm{T}}{\Vert ({\hat{{{\varvec{v}}}}}^\star {\hat{{{\varvec{v}}}}}^\star {}^\mathrm{T}-I)Y_k{\hat{{\varvec{e}}}}_j\Vert _{\ell _2}^3} \right] (I-{\hat{{{\varvec{v}}}}}^\star {\hat{{{\varvec{v}}}}}^\star {}^\mathrm{T}) \end{aligned}$$

and

7.31 $$\begin{aligned}&\displaystyle \frac{\partial {{\varvec{G}}}}{\partial {{\varvec{v}}}}({\varvec{d}}_1^\star ,\ldots , {\varvec{d}}_n^\star ,{\hat{{{\varvec{v}}}}}^\star )\nonumber \\&\quad = \frac{1}{\Vert {{\varvec{g}}}^\star \Vert _{\ell _2}}\left( I-\frac{{{\varvec{g}}}^\star {{\varvec{g}}}^\star {}^\mathrm{T}}{\Vert {{\varvec{g}}}^\star \Vert _{\ell _2}} \right) \frac{\partial {{\varvec{g}}}}{\partial {{\varvec{v}}}}({\varvec{d}}_1^\star ,\ldots , {\varvec{d}}_n^\star ,{\hat{{{\varvec{v}}}}}^\star ) \nonumber \\&\displaystyle = (I-{\hat{{{\varvec{v}}}}}^\star {\hat{{{\varvec{v}}}}}^\star {}^\mathrm{T}) \frac{\partial {{\varvec{g}}}}{\partial {{\varvec{v}}}}({\varvec{d}}_1^\star ,\ldots , {\varvec{d}}_n^\star ,{\hat{{{\varvec{v}}}}}^\star )\nonumber \\&\displaystyle \mathop {\longrightarrow }\limits ^{\tau \rightarrow \infty } (I-{\hat{{{\varvec{v}}}}}^\star {\hat{{{\varvec{v}}}}}^\star {}^\mathrm{T})\left[ I-\rho \sum _{j=1}^n ({\hat{{{\varvec{v}}}}}^\star {}^\mathrm{T}Y_k{\hat{{\varvec{e}}}}_j)^2\right. \nonumber \\&\left. \quad \frac{Y_k{\hat{{\varvec{e}}}}_j {\hat{{\varvec{e}}}}_j^\mathrm{T}Y_k^\mathrm{T}}{\Vert ({\hat{{{\varvec{v}}}}}^\star {\hat{{{\varvec{v}}}}}^\star {}^\mathrm{T}-I)Y_k{\hat{{\varvec{e}}}}_j\Vert _{\ell _2}^3} \right] (I-{\hat{{{\varvec{v}}}}}^\star {\hat{{{\varvec{v}}}}}^\star {}^\mathrm{T})=:B. \end{aligned}$$

Thus, the Jacobian satisfies

7.32 $$\begin{aligned}&\frac{\partial ({{\varvec{F}}}_1,\ldots ,{{\varvec{F}}}_n,{{\varvec{G}}})}{\partial ({\varvec{d}}_1,\ldots ,{\varvec{d}}_n,{{\varvec{\mu }}})}({\varvec{d}}_1^\star ,\ldots , {\varvec{d}}_n^\star ,{{\varvec{\mu }}}^\star )\nonumber \\&\quad \mathop {\longrightarrow }\limits ^{\tau
 \rightarrow \infty } \left[ \begin{array}{cccc} 0 &{} \cdots &{} 0 &{} A_1 \\ \vdots &{} \ddots &{} &{} \vdots \\ 0 &{} &{} 0 &{} A_n \\ 0 &{} \cdots &{} 0 &{} B \\ \end{array} \right] =: J. \end{aligned}$$

By the definition $S_k = {\mathcal R}(Y_k)$ prior to (4.3), it follows from (7.27) that $A_j:S_k\rightarrow S_k$, $j=1,\ldots ,n$, and from (7.31) that $B:S_k\rightarrow S_k$. In the same way, it follows from (7.32) that $J:(S_k)^{n+1}\rightarrow (S_k)^{n+1}$. It will be shown that $B:S_k\rightarrow S_k$ has spectrum in $(-1,1)$. Let *B* be expressed as $B = (I-{\hat{{{\varvec{v}}}}}^\star {\hat{{{\varvec{v}}}}}^\star {}^\mathrm{T}) - \rho \hat{C}$ where

7.33 $$\begin{aligned} \hat{C}= & {} (I-{\hat{{{\varvec{v}}}}}^\star {\hat{{{\varvec{v}}}}}^\star {}^\mathrm{T})C (I-{\hat{{{\varvec{v}}}}}^\star {\hat{{{\varvec{v}}}}}^\star {}^\mathrm{T}), \nonumber \\ C= & {} \sum _{j=1}^n ({\hat{{{\varvec{v}}}}}^\star {}^\mathrm{T}Y_k{\hat{{\varvec{e}}}}_j)^2 \frac{Y_k{\hat{{\varvec{e}}}}_j {\hat{{\varvec{e}}}}_j^\mathrm{T}Y_k^\mathrm{T}}{\Vert ({\hat{{{\varvec{v}}}}}^\star {\hat{{{\varvec{v}}}}}^\star {}^\mathrm{T}-I)Y_k{\hat{{\varvec{e}}}}_j\Vert _{\ell _2}^3}. \end{aligned}$$

Suppose there were ${{\varvec{v}}} \in S_k$ for which ${{\varvec{v}}}^\mathrm{T}C{{\varvec{v}}} = 0$. Then by (4.2) and (4.3), ${{\varvec{v}}}^\mathrm{T}Y_k={{\varvec{v}}}^\mathrm{T}(I-V_{k-1}V_{k-1}^\mathrm{T})Y_\mathrm{c} = {{\varvec{v}}}^\mathrm{T}Y_\mathrm{c}$ and with (7.19),

7.34 $$\begin{aligned} 0= & {} \sum _{j=1}^n ({\hat{{{\varvec{v}}}}}^\star {}^\mathrm{T}Y_k{\hat{{\varvec{e}}}}_j)^2 \frac{|{{\varvec{v}}}^\mathrm{T}Y_\mathrm{c}{\hat{{\varvec{e}}}}_j|^2}{\Vert ({\hat{{{\varvec{v}}}}}^\star {\hat{{{\varvec{v}}}}}^\star {}^\mathrm{T}-I)Y_k{\hat{{\varvec{e}}}}_j\Vert } \nonumber \\&\quad \ge \Vert {{\varvec{v}}}^\mathrm{T}Y_\mathrm{c}\Vert _{\ell _2}^2 \left[ \min _{1\le j\le n} \frac{({\hat{{{\varvec{v}}}}}^\star {}^\mathrm{T}Y_k{\hat{{\varvec{e}}}}_j)^2}{\Vert ({\hat{{{\varvec{v}}}}}^\star {\hat{{{\varvec{v}}}}}^\star {}^\mathrm{T}-I)Y_k{\hat{{\varvec{e}}}}_j\Vert } \right] _{>0}, \end{aligned}$$

and ${{\varvec{v}}}^\mathrm{T}Y_\mathrm{c}=0$ would contradict the assumption that $S_1 = {\mathcal R}(Y_\mathrm{c}) = \mathbb {R}^m$. Thus, there exist $0<\lambda _\mathrm{min}\le \lambda _\mathrm{max}$ such that *C* satisfies

7.35 $$\begin{aligned} \lambda _\mathrm{min}\Vert {{\varvec{x}}}\Vert _{\ell _2}^2 \le {{\varvec{x}}}^\mathrm{T}C{{\varvec{x}}} \le \lambda _\mathrm{max}\Vert {{\varvec{x}}}\Vert _{\ell _2}^2, \quad \forall {{\varvec{x}}}\in S_k. \end{aligned}$$

Let $S_k = U_k \oplus W_k$ where $W_k = \text{ span }\{{\hat{{{\varvec{v}}}}}^\star \}$. Clearly, $\lambda = 0$ is an eigenvalue of *B* associated with the eigenvector ${\hat{{{\varvec{v}}}}}^\star \in W_k$. Eigenvalues for eigenvectors in $U_k$ will now be estimated. Let $\rho$ be small enough that $-1 < 1-\rho \lambda _\mathrm{max} < 1-\rho \lambda _\mathrm{min} < 1$. Then for ${{\varvec{x}}} \in U_k$ it follows with the definition of $\hat{C}$ and *C* that ${{\varvec{x}}}^\mathrm{T}B{{\varvec{x}}} = \Vert {{\varvec{x}}}\Vert _{\ell _2}^2 - \rho {{\varvec{x}}}^\mathrm{T}\hat{C}{{\varvec{x}}} = \Vert {{\varvec{x}}}\Vert _{\ell _2}^2 - \rho {{\varvec{x}}}^\mathrm{T}C{{\varvec{x}}}$ and

7.36 $$\begin{aligned}&-\Vert {{\varvec{x}}}\Vert _{\ell _2}^2 < (1-\rho \lambda _\mathrm{max})\Vert {{\varvec{x}}}\Vert _{\ell _2}^2 \le \Vert {{\varvec{x}}}\Vert _{\ell _2}^2 - \rho {{\varvec{x}}}^\mathrm{T}C{{\varvec{x}}} \nonumber \\&\quad \le (1-\rho \lambda _\mathrm{min})\Vert {{\varvec{x}}}\Vert _{\ell _2}^2 < \Vert {{\varvec{x}}}\Vert _{\ell _2}^2. \end{aligned}$$

Thus, the spectrum of $B:S_k\rightarrow S_k$ lies in $(-1,1)$. Recalling that the dimension of $S_k$ is $m-k+1$, let $P \in \mathbb {R}^{m\times (m-k+1)}$ be chosen with orthonormal columns such that $P^\mathrm{T}B P= \Lambda = \text{ diag }\{\lambda _i\}_{i=1}^{m-k+1}$ where $\lambda _i \in (-1,1)$. Also set $Z \in \mathbb {R}^{m\times (m-k+1)}$ with all zero entries. Further let $I\in \mathbb {R}^{m\times m}$ and $0\in \mathbb {R}^{m\times m}$ in (7.37) below denote the identity and the zero matrix, respectively. Then the matrix *J* satisfies

7.37 $$\begin{aligned} \left[ \begin{array}{cccc} I &{} \cdots &{} &{} Z \\ \vdots &{} \ddots &{} &{} \vdots \\ 0 &{} &{} I &{} Z \\ 0 &{} \cdots &{} 0 &{} P \\ \end{array} \right] ^\mathrm{T}J\left[ \begin{array}{cccc} I &{} \cdots &{} &{} Z \\ \vdots &{} \ddots &{} &{} \vdots \\ 0 &{} &{} I &{} Z \\ 0 &{} \cdots &{} 0 &{} P \\ \end{array} \right] = \left[ \begin{array}{cccc} 0 &{} \cdots &{} 0 &{} A_1P \\ \vdots &{} \ddots &{} &{} \vdots \\ 0 &{} &{} 0 &{} A_nP \\ Z^\mathrm{T}Z &{} \cdots &{} Z^\mathrm{T}Z &{} \Lambda \\ \end{array} \right] \nonumber \\ \end{aligned}$$

proving that the spectrum of *J* lies in $(-1,1)$. Since the Jacobian in (6.26) is arbitrarily well approximated by *J* when $\tau$ is sufficiently large, its spectrum lies strictly within the ball of radius 1 for $\tau$ sufficiently large and for $\rho$ sufficiently small. Because of the assumption (7.19), the mapping (7.23)–(7.24) is smooth in a neighborhood of the fixed point $\{D^\star ,{\hat{{{\varvec{v}}}}}^\star \}$, and hence the iterates $\{D_l,{\hat{{{\varvec{v}}}}}_l\}$ converge to the fixed point when started sufficiently close to it. $\square$

### *Remark 2*

Computations demonstrate that the iteration (4.10)–(4.11) can converge to a minimizer for $H_k$ even when the condition (7.19) is violated. Such a case is illustrated in Fig. 4. Variations of the example illustrated in Fig. 4, in which data points lie on the boundary of a rectangle instead of a square, indicate an advantage to having sphered the data by $\ell _2$ means according to (2.8) and (2.10) before proceeding with the methods of Sect. 4.

## Convergence of the Iterative Scheme for $\ell _1$ ICA

The analysis of the scheme (5.11) begins with establishing existence of a maximizer for $G_l$ in (5.9). Recall the assumption in Sect. 5 that $T_1 = {\mathcal R}(Y_\mathrm{s}) = \mathbb {R}^m$ so that $T_l = {\mathcal R}(Y_l) = {\mathcal R}(U_{l-1}^\mathrm{T})^\perp$, $l=2,\ldots ,m$, as seen in (5.7). Also, define

8.1 $$\begin{aligned} \mathbb {T}_l = \{{\hat{{{\varvec{u}}}}}\in T_l: \Vert \hat{{{\varvec{u}}}}\Vert _{\ell _2}=1\}. \end{aligned}$$

### **Lemma 6**

For $G_l$ in (5.9), there exists a maximizer ${\hat{{{\varvec{u}}}}}^\star$ over $T_l$ satisfying ${\hat{{{\varvec{u}}}}}^\star \in \mathbb {T}_l$.

### *Proof*

Since ${\hat{{{\varvec{u}}}}}^\mathrm{T}Y_l = {\hat{{{\varvec{u}}}}}^\mathrm{T} (I-U_{l-1}^\mathrm{T}U_{k-1})Y_\mathrm{s} = {\hat{{{\varvec{u}}}}}^\mathrm{T}Y_\mathrm{s}$ holds for each *l*, the assumption $T_1 = {\mathcal R}(Y_\mathrm{s}) = \mathbb {R}^m$ implies that there is a ${\hat{{{\varvec{u}}}}} \in \mathbb {T}_l$ satisfying $G_l({\hat{{{\varvec{u}}}}})\ne 0$. Thus, $G_l$ is not maximized at ${{\varvec{u}}}={{\varvec{0}}}$. According to (5.9), $G_l$ is constant along rays outside the origin. Thus, the maximization can as well be carried out on $\mathbb {T}_l$. The claim follows since $\mathbb {T}_l$ is compact and $G_l$ is continuous on $\mathbb {T}_l$. $\square$

### **Lemma 7**

For any ${{\varvec{u}}} \in T_l \backslash \{0\}$, the directional derivative of $G_l$ in the direction of ${\varvec{w}} \in T_l$ is given by

8.2 $$\begin{aligned} \partial G_l({{\varvec{u}}};{\varvec{w}})= & {} \frac{1}{\Vert {{\varvec{u}}}\Vert _{\ell _2}} \left[ \sum _{j=1}^n \delta _j({{\varvec{u}}},{\varvec{w}}) Y_l{\hat{{\varvec{e}}}}_j\right. \nonumber \\&\left. - \sum _{j=1}^n \frac{|{\hat{{\varvec{e}}}}_j^\mathrm{T}Y_l^\mathrm{T}{{\varvec{u}}}|}{\Vert {{\varvec{u}}}\Vert _{\ell _2}} \frac{{{\varvec{u}}}}{\Vert {{\varvec{u}}}\Vert _{\ell _2}}, \right] ^\mathrm{T}{\varvec{w}} \end{aligned}$$

where

8.3 $$\begin{aligned} \delta _j({{\varvec{u}}},{\varvec{w}}) = \left\{ \begin{array}{r@{\quad }l} \sigma ({\hat{{\varvec{e}}}}_j^\mathrm{T}Y_l^\mathrm{T}{{\varvec{u}}}), &{} {\hat{{\varvec{e}}}}_j^\mathrm{T}Y_l^\mathrm{T}{{\varvec{u}}} \ne 0 \\ \sigma ({\hat{{\varvec{e}}}}_j^\mathrm{T}Y_l^\mathrm{T}{\varvec{w}}), &{} {\hat{{\varvec{e}}}}_j^\mathrm{T}Y_l^\mathrm{T}{{\varvec{u}}} = 0 \end{array} \right. \quad \sigma (t) = \text{ sign }(t).\nonumber \\ \end{aligned}$$

### *Proof*

For ${{\varvec{u}}} \in T_l\backslash \{0\}$, ${\varvec{w}} \in T_l$, and $t>0$ sufficiently small that ${{\varvec{u}}} +t{\varvec{w}} \ne 0$,

8.4 $$\begin{aligned}&G_l({{\varvec{u}}}+t{\varvec{w}})-G_l({{\varvec{u}}})= \sum _{j=1}^n \frac{|{\hat{{\varvec{e}}}}_j^\mathrm{T}Y_l^\mathrm{T}({{\varvec{u}}}+t{\varvec{w}})|- |{\hat{{\varvec{e}}}}_j^\mathrm{T}Y_l^\mathrm{T}{{\varvec{u}}}|}{\Vert {{\varvec{u}}}+t{\varvec{w}}\Vert _{\ell _2}}\nonumber \\&\quad -\sum _{j=1}^n|{\hat{{\varvec{e}}}}_j^\mathrm{T}Y_l^\mathrm{T}{{\varvec{u}}}| \frac{\Vert {{\varvec{u}}}+t{\varvec{w}}\Vert _{\ell _2}-\Vert {{\varvec{u}}}\Vert _{\ell _2}}{\Vert {{\varvec{u}}}+t{\varvec{w}}\Vert _{\ell _2} \Vert {{\varvec{u}}}\Vert _{\ell _2}}. \end{aligned}$$

For ${\hat{{\varvec{e}}}}_j^\mathrm{T}Y_l^\mathrm{T}{{\varvec{u}}} \ne 0$, the terms of the first sum in (8.4) satisfy

8.5 $$\begin{aligned}&\displaystyle \lim _{t\rightarrow 0^+} \frac{|{\hat{{\varvec{e}}}}_j^\mathrm{T}Y_l^\mathrm{T}({{\varvec{u}}}+t{\varvec{w}})|- |{\hat{{\varvec{e}}}}_j^\mathrm{T}Y_l^\mathrm{T}{{\varvec{u}}}|}{t\Vert {{\varvec{u}}}+t{\varvec{w}}\Vert _{\ell _2}}\nonumber \\&\quad = \displaystyle \lim _{t\rightarrow 0^+} \frac{(2t{\hat{{\varvec{e}}}}_j^\mathrm{T}Y_l^\mathrm{T}{{\varvec{u}}}+t^2{\hat{{\varvec{e}}}}_j^\mathrm{T}Y_l^\mathrm{T}{{\varvec{u}}})({\hat{{\varvec{e}}}}_j^\mathrm{T}Y_l^\mathrm{T}{{\varvec{u}}})/t}{(|{\hat{{\varvec{e}}}}_j^\mathrm{T}Y_l^\mathrm{T}({{\varvec{u}}}+t{\varvec{w}})|+|{\hat{{\varvec{e}}}}_j^\mathrm{T}Y_l^\mathrm{T}{{\varvec{u}}}|)\Vert {{\varvec{u}}}+t{\varvec{w}}\Vert _{\ell _2}} \nonumber \\&\quad = \displaystyle \sigma ({\hat{{\varvec{e}}}}_j^\mathrm{T}Y_l^\mathrm{T}{{\varvec{u}}})\frac{{\hat{{\varvec{e}}}}_j^\mathrm{T}Y_l^\mathrm{T} {\varvec{w}}}{\Vert {{\varvec{u}}}\Vert _{\ell _2}} \end{aligned}$$

and for $${\hat{{\varvec{e}}}}_j^\mathrm{T}Y_l^\mathrm{T}{{\varvec{u}}} = 0$$,

8.6 $$\begin{aligned}&\lim _{t\rightarrow 0^+} \frac{|{\hat{{\varvec{e}}}}_j^\mathrm{T}Y_l^\mathrm{T}({{\varvec{u}}}+t{\varvec{w}})|- |{\hat{{\varvec{e}}}}_j^\mathrm{T}Y_l^\mathrm{T}{{\varvec{u}}}|}{t\Vert {{\varvec{u}}}+t{\varvec{w}}\Vert _{\ell _2}} \nonumber \\&\quad = \frac{|{\hat{{\varvec{e}}}}_j^\mathrm{T}Y_l^\mathrm{T}{\varvec{w}}|}{\Vert {{\varvec{u}}}\Vert _{\ell _2}} = \sigma ({\hat{{\varvec{e}}}}_j^\mathrm{T}Y_l^\mathrm{T}{\varvec{w}}) \frac{{\hat{{\varvec{e}}}}_j^\mathrm{T}Y_l^\mathrm{T}{\varvec{w}}}{\Vert {{\varvec{u}}}\Vert _{\ell _2}}. \end{aligned}$$

The terms of the second sum in (8.4) satisfy

8.7 $$\begin{aligned}&\lim _{t\rightarrow 0^+} \frac{\Vert {{\varvec{u}}}+t{\varvec{w}}\Vert _{\ell _2}-\Vert {{\varvec{u}}}\Vert _{\ell _2}}{t\Vert {{\varvec{u}}}+t{\varvec{w}}\Vert _{\ell _2} \Vert {{\varvec{u}}}\Vert _{\ell _2}}\nonumber \\&\quad = \lim _{t\rightarrow 0^+} \frac{(2t{{\varvec{u}}}^\mathrm{T}{\varvec{w}}+t^2\Vert {\varvec{w}}\Vert _{\ell _2}^2)/t}{\Vert {{\varvec{u}}}+t{\varvec{w}}\Vert _{\ell _2} \Vert {{\varvec{u}}}\Vert _{\ell _2}(\Vert {{\varvec{u}}}+t{\varvec{w}}\Vert _{\ell _2}+\Vert {{\varvec{u}}}\Vert _{\ell _2})}\nonumber \\&\quad = \frac{{{\varvec{u}}}^\mathrm{T}{\varvec{w}}}{\Vert {{\varvec{u}}}\Vert _{\ell _2}^3}. \end{aligned}$$

Combining these calculations gives (8.2). $\square$

Lemma 7 is now used to prove Lemma 8. For the following, let $\sigma ({{\varvec{v}}}) = \{\sigma (v_i)\}$ where ${{\varvec{v}}} = \{v_i\}$ and $\sigma (t) = \text{ sign }(t)$.

### **Lemma 8**

The first-order necessary optimality condition for a maximizer ${\hat{{{\varvec{u}}}}}^\star$ of $G_l$ over $T_l$ satisfying ${\hat{{{\varvec{u}}}}}^\star \in \mathbb {T}_l$ as given by Lemma 6 is

8.8 $$\begin{aligned} Y_l \sigma (Y_l^\mathrm{T}{\hat{{{\varvec{u}}}}}^\star ) = \Vert Y_l^\mathrm{T}{\hat{{{\varvec{u}}}}}^\star \Vert _{\ell _1} {\hat{{{\varvec{u}}}}}^\star \end{aligned}$$

and the sets ${\mathcal Y} = \{j: Y_l{\hat{{\varvec{e}}}}_j={{\varvec{0}}}\}$ and ${\mathcal S} = \{j: {{\varvec{u}}}^\star {}^\mathrm{T}Y_l {\hat{{\varvec{e}}}}_j=0\}$ agree. As a consequence, $\exists \epsilon > 0$ such that

8.9 $$\begin{aligned} \nabla G_l({{\varvec{u}}})= & {} \frac{1}{\Vert {{\varvec{u}}}\Vert _{\ell _2}} \left[ Y_l \sigma (Y_l^\mathrm{T}{{\varvec{u}}})\right. \nonumber \\&\left. - \frac{\Vert Y_l^\mathrm{T}{{\varvec{u}}}\Vert _{\ell _1}}{\Vert {{\varvec{u}}}\Vert _{\ell _2}} \frac{{{\varvec{u}}}}{\Vert {{\varvec{u}}}\Vert _{\ell _2}} \right] , \quad \forall {{\varvec{u}}} \in B({\hat{{{\varvec{u}}}}}^\star ,\epsilon ). \end{aligned}$$

### *Proof*

Let ${\hat{{{\varvec{u}}}}}^\star \in \mathbb {T}_l$ be a maximizer for $G_l$ guaranteed by Lemma 6. By (8.2),

8.10 $$\begin{aligned} \partial G_l({\hat{{{\varvec{u}}}}}^\star ;{\varvec{w}})= & {} \lim _{t\rightarrow 0^+} \frac{G_l({\hat{{{\varvec{u}}}}}^\star +t{\varvec{w}})-G_l({\hat{{{\varvec{u}}}}}^\star )}{t}\nonumber \\\le & {} 0, \quad \forall {\varvec{w}} \in T_l. \end{aligned}$$

By decomposing sums into indices in ${\mathcal S}$ and ${\mathcal S}^\mathrm{c}$,

8.11 $$\begin{aligned}&\partial G_l({\hat{{{\varvec{u}}}}}^\star ;{\varvec{w}}) \nonumber \\&\quad \displaystyle = \left[ \sum _{j\in {\mathcal S}} \sigma ({\hat{{\varvec{e}}}}_j^\mathrm{T}Y_l^\mathrm{T}{\varvec{w}})Y_l{\hat{{\varvec{e}}}}_j + \sum _{j\in {\mathcal S}^\mathrm{c}} \sigma ({\hat{{\varvec{e}}}}_j^\mathrm{T}Y_l^\mathrm{T}{\hat{{{\varvec{u}}}}}^\star ) Y_l{\hat{{\varvec{e}}}}_j\right. \nonumber \\&\quad \left. -\sum _{j=1}^n |{\hat{{\varvec{e}}}}_j^\mathrm{T}Y_l^\mathrm{T}{\hat{{{\varvec{u}}}}}^\star |\hat{{{\varvec{u}}}}^\star \right] ^\mathrm{T}{\varvec{w}}\nonumber \\&\quad \displaystyle = \sum _{j\in {\mathcal S}} |{\hat{{\varvec{e}}}}_j^\mathrm{T}Y_l^\mathrm{T}{\varvec{w}}| +\left[ \sum _{j\in {\mathcal S}^\mathrm{c}} \sigma ({\hat{{\varvec{e}}}}_j^\mathrm{T} Y_l^\mathrm{T}{\hat{{{\varvec{u}}}}}^\star ) Y_l{\hat{{\varvec{e}}}}_j\right. \nonumber \\&\qquad \left. + \sum _{j\in {\mathcal S}} \underbrace{\sigma ({\hat{{\varvec{e}}}}_j^\mathrm{T}Y_l^\mathrm{T}{\hat{{{\varvec{u}}}}}^\star )}_{=0} Y_l{\hat{{\varvec{e}}}}_j -\sum _{j=1}^n |{\hat{{\varvec{e}}}}_j^\mathrm{T}Y_l^\mathrm{T}{\hat{{{\varvec{u}}}}}^\star |\hat{{{\varvec{u}}}}^\star \right] ^\mathrm{T}{\varvec{w}}\nonumber \\&\quad \displaystyle = \sum _{j\in {\mathcal S}} |{\hat{{\varvec{e}}}}_j^\mathrm{T}Y_l^\mathrm{T}{\varvec{w}}| + {{\varvec{v}}}^\star {}^\mathrm{T}{\varvec{w}}, \end{aligned}$$

where

8.12 $$\begin{aligned} {{\varvec{v}}}^\star= & {} \sum _{j=1}^n \sigma ({\hat{{\varvec{e}}}}_j^\mathrm{T}Y_l^\mathrm{T}{\hat{{{\varvec{u}}}}}^\star ) Y_l{\hat{{\varvec{e}}}}_j -\sum _{j=1}^n |{\hat{{\varvec{e}}}}_j^\mathrm{T}Y_l^\mathrm{T}{\hat{{{\varvec{u}}}}}^\star |\hat{{{\varvec{u}}}}^\star \nonumber \\= & {} Y_l\sigma (Y_l^\mathrm{T}{\hat{{{\varvec{u}}}}}^\star ) - \Vert Y_l^\mathrm{T}{\hat{{{\varvec{u}}}}}^\star \Vert _{\ell _1} {\hat{{{\varvec{u}}}}}^\star . \end{aligned}$$

Combining (8.10) and (8.11), it follows

8.13 $$\begin{aligned} \forall i\in {\mathcal S} \quad |{\hat{{\varvec{e}}}}_i^\mathrm{T}Y_l^\mathrm{T}{\varvec{w}}| \le \sum _{j\in {\mathcal S}} |{\hat{{\varvec{e}}}}_j^\mathrm{T}Y_l^\mathrm{T}{\varvec{w}}| \le 0, \quad \forall {\varvec{w}} \in {{\varvec{v}}}^\star {}^\perp .\nonumber \\ \end{aligned}$$

This implies the existence of $\{\gamma _j\}_{j \in {\mathcal S}}$ (possibly zero) such that

8.14 $$\begin{aligned} Y_l{\hat{{\varvec{e}}}}_j = \gamma _j {{\varvec{v}}}^\star , \quad \forall j\in {\mathcal S}. \end{aligned}$$

Now with ${\varvec{w}} = {{\varvec{v}}}^\star$, combining (8.10), (8.11), and (8.14) gives

8.15 $$\begin{aligned} 0 \ge \sum _{j\in {\mathcal S}}|\gamma _j|\Vert {{\varvec{v}}}^\star \Vert _{\ell _2}^2 + \Vert {{\varvec{v}}}^\star \Vert _{\ell _2}^2 \end{aligned}$$

or ${{\varvec{v}}}^\star = {{\varvec{0}}}$. With (8.12), the optimality condition (8.8) follows.

According to (8.14), ${\mathcal S} \subset {\mathcal Y}$ holds. Since ${\mathcal Y} \subset {\mathcal S}$ always holds, it follows that ${\mathcal Y} = {\mathcal S}$. Note that

8.16 $$\begin{aligned} G_l({{\varvec{u}}}) = \frac{1}{\Vert {{\varvec{u}}}\Vert _{\ell _2}} \sum _{j=1}^n |{\hat{{\varvec{e}}}}_j^\mathrm{T}Y_l^\mathrm{T}{{\varvec{u}}}| = \frac{1}{\Vert {{\varvec{u}}}\Vert _{\ell _2}} \sum _{j\in {\mathcal Y}^\mathrm{c}} |{\hat{{\varvec{e}}}}_j^\mathrm{T}Y_l^\mathrm{T}{{\varvec{u}}}| \end{aligned}$$

is smooth for ${{\varvec{u}}} \in B({\hat{{{\varvec{u}}}}}^\star ,\epsilon )$ for some $\epsilon > 0$. As a result, $G_l$ is smooth in $B({\hat{{{\varvec{u}}}}}^\star ,\epsilon )$ and can be differentiated directly to obtain (8.9). $\square$

Turning to the iteration (5.11), we observe that if convergence to a fixed point ${\hat{{{\varvec{u}}}}}^\star$ can be guaranteed, then multiplying ${\hat{{{\varvec{u}}}}}^\star {}^\mathrm{T}$ by $\Vert {{\varvec{u}}}^\star \Vert {\hat{{{\varvec{u}}}}}^\star = {{\varvec{u}}}^\star = {\hat{{{\varvec{u}}}}}^\star + \tau [Y_l\sigma (Y_l^\mathrm{T}{\hat{{{\varvec{u}}}}}^\star ) - \hat{{{\varvec{u}}}}^\star \Vert {\hat{{{\varvec{u}}}}}^\star {}^\mathrm{T}Y_l\Vert _{\ell _1}]$ gives

8.17 $$\begin{aligned} \Vert {{\varvec{u}}}^\star \Vert _{\ell _2}= & {} {\hat{{{\varvec{u}}}}}^\star {}^\mathrm{T}{{\varvec{u}}}^\star = 1 + \tau [ {\hat{{{\varvec{u}}}}}^\star {}^\mathrm{T}Y_l\sigma (Y_l^\mathrm{T}{\hat{{{\varvec{u}}}}}^\star ) \nonumber \\&- \Vert {\hat{{{\varvec{u}}}}}^\star {}^\mathrm{T}Y_l\Vert _{\ell _1}]=1 \end{aligned}$$

where the last equality follows with $(Y_l^\mathrm{T}{\hat{{{\varvec{u}}}}}^\star )^\mathrm{T}\sigma (Y_l^\mathrm{T}{\hat{{{\varvec{u}}}}}^\star ) = \Vert {\hat{{{\varvec{u}}}}}^\star {}^\mathrm{T}Y_l\Vert _{\ell _1}$. Since $\Vert {{\varvec{u}}}^\star \Vert _{\ell _2} = \Vert {\hat{{{\varvec{u}}}}}^\star \Vert _{\ell _2} = 1$ holds, it follows that ${{\varvec{u}}}^\star = {\hat{{{\varvec{u}}}}}^\star$ and hence ${\hat{{{\varvec{u}}}}}^\star$ satisfies (8.8). The following theorem provides sufficient conditions under which convergence of our algorithm to a fixed point can be guaranteed.

### **Theorem 3**

Let ${\hat{{{\varvec{u}}}}}^\star \in \mathbb {T}_l$ satisfy (8.8) where the set ${\mathcal S} = \{j: {\hat{{\varvec{e}}}}_j^\mathrm{T}Y_l^\mathrm{T}{\hat{{{\varvec{u}}}}}^\star = 0\}$ is empty. Then ${\hat{{{\varvec{u}}}}}^\star$ is a fixed point of the iteration (5.11), and for $\tau$ sufficiently small, the iterates $\{{\hat{{{\varvec{u}}}}}_k\}$ converge to this fixed point when $\{{\hat{{{\varvec{u}}}}}_0\}$ starts the iteration close enough to $\{{\hat{{{\varvec{u}}}}}^\star \}$.

### *Proof*

It will first be shown that $\{{\hat{{{\varvec{u}}}}}^\star \}$ is a fixed point for the iteration (5.11) which is locally asymptotically stable for $\tau$ sufficiently small. Setting $\hat{\mathbf{u}}_{k+1} = {\hat{{{\varvec{u}}}}}^\star$ on the right side of (5.11) and using (8.8) shows that $\mathbf{u}_{k+1} = {\hat{{{\varvec{u}}}}}^\star$. Hence with $\Vert \mathbf{u}_{k+1}\Vert _{\ell _2} = \Vert {\hat{{{\varvec{u}}}}}^\star \Vert _{\ell _2} = 1$ it follows that $\hat{\mathbf{u}}_{k+1} = \mathbf{u}_{k+1} = {\hat{{{\varvec{u}}}}}^\star$, and thus ${\hat{{{\varvec{u}}}}}^\star$ is a fixed point. To establish the stability of the fixed point, define

8.18 $$\begin{aligned} {{\varvec{G}}}({{\varvec{u}}})= & {} \frac{{{\varvec{g}}}({{\varvec{u}}})}{\Vert {{\varvec{g}}}({{\varvec{u}}})\Vert _{\ell _2}}, \nonumber \\ {{\varvec{g}}}({{\varvec{u}}})= & {} {{\varvec{u}}} + \tau [Y_l\, \sigma (Y_l^\mathrm{T} {{\varvec{u}}}) - {{\varvec{u}}} \Vert Y_l^\mathrm{T}{{\varvec{u}}}\Vert _{\ell _1}] \end{aligned}$$

so that (5.11) is given by

8.19 $$\begin{aligned} \hat{\mathbf{u}}_{k+1} = {{\varvec{G}}}(\hat{\mathbf{u}}_k). \end{aligned}$$

The claimed stability will follow once it is shown that the Jacobian of this mapping evaluated at ${\hat{{{\varvec{u}}}}}^\star$ has spectral radius less than 1 when $\tau$ is sufficiently small. The Jacobian is given by

8.20 $$\begin{aligned} \frac{\partial {{\varvec{G}}}}{\partial {{\varvec{u}}}} = \frac{1}{\Vert {{\varvec{g}}}({{\varvec{u}}})\Vert _{\ell _2}}\left( I - \frac{{{\varvec{g}}}({{\varvec{u}}}){{\varvec{g}}}^\mathrm{T}({{\varvec{u}}})}{\Vert {{\varvec{g}}}({{\varvec{u}}})\Vert _{\ell _2}^2}\right) \frac{\partial {{\varvec{g}}}}{\partial {{\varvec{u}}}}({{\varvec{u}}}) \end{aligned}$$

where with $$\{j: {\hat{{\varvec{e}}}}_j^\mathrm{T}Y_l^\mathrm{T}{{\varvec{u}}}=0\} = \emptyset $$,

8.21 $$\begin{aligned} \frac{\partial {{\varvec{g}}}}{\partial {{\varvec{u}}}}({{\varvec{u}}}) = (1-\tau \Vert Y_l^\mathrm{T}{{\varvec{u}}}\Vert _{\ell _1})I - \tau {{\varvec{u}}} \sum _{j=1}^n \sigma ({\hat{{\varvec{e}}}}_j^\mathrm{T}Y_l^\mathrm{T}{{\varvec{u}}}) {\hat{{\varvec{e}}}}_j^\mathrm{T}Y_l^\mathrm{T}.\nonumber \\ \end{aligned}$$

It follows with (8.8) that ${{\varvec{g}}}({\hat{{{\varvec{u}}}}}^\star ) = \hat{{{\varvec{u}}}}^\star$ and thus $\Vert {{\varvec{g}}}({\hat{{{\varvec{u}}}}}^\star )\Vert _{\ell _2} = \Vert {\hat{{{\varvec{u}}}}}^\star \Vert _{\ell _2} = 1$. The Jacobian of ${{\varvec{G}}}$ at ${\hat{{{\varvec{u}}}}}^\star$ is symmetric and is given by

8.22 $$\begin{aligned} \frac{\partial {{\varvec{G}}}}{\partial {{\varvec{u}}}}({\hat{{{\varvec{u}}}}}^\star ) = (1-\tau \Vert Y_l^\mathrm{T}{\hat{{{\varvec{u}}}}}^\star \Vert ) (I-{\hat{{{\varvec{u}}}}}^\star \hat{{{\varvec{u}}}}^\star {}^\mathrm{T}). \end{aligned}$$

For ${{\varvec{v}}} \in \mathbb {R}^m$, it follows with $0\le \Vert (I-{\hat{{{\varvec{u}}}}}^\star \hat{{{\varvec{u}}}}^\star {}^\mathrm{T}){{\varvec{v}}}\Vert _{\ell _2}^2 = {{\varvec{v}}}^\mathrm{T}(I-{\hat{{{\varvec{u}}}}}^\star \hat{{{\varvec{u}}}}^\star {}^\mathrm{T}){{\varvec{v}}} \le \Vert {{\varvec{v}}}\Vert _{\ell _2}^2$ that the Jacobian satisfies

8.23 $$\begin{aligned} 0\le & {} {{\varvec{v}}}^\mathrm{T}\frac{\partial {{\varvec{G}}}}{\partial {{\varvec{u}}}}({\hat{{{\varvec{u}}}}}^\star ){{\varvec{v}}}\nonumber \\\le & {} (1-\tau \Vert Y_l^\mathrm{T}{\hat{{{\varvec{u}}}}}^\star \Vert _{\ell _1})\Vert {{\varvec{v}}}\Vert _{\ell _2}^2 < \Vert {{\varvec{v}}}\Vert _{\ell _2}^2, \quad \forall {{\varvec{v}}}\in \mathbb {R}^m \end{aligned}$$

provided that $1>\tau \Vert {\hat{{{\varvec{u}}}}}^\star {}^\mathrm{T}Y_l\Vert _{\ell _1}$. For any such $\tau$, the spectral radius of the Jacobian in (8.22) is less than 1. Because of the assumption that ${\mathcal S}$ is empty, the mapping (8.18) is smooth in a neighborhood of the fixed point $\{{\hat{{{\varvec{u}}}}}^\star \}$, and hence the iterates $\{{\hat{{{\varvec{u}}}}}_k\}$ converge to the fixed point when started sufficiently close to it. $\square$

### *Remark 3*

Computations demonstrate that the iteration (5.11) converges to a maximizer for $G_l$ even when the condition ${\mathcal S} = \{j: {\hat{{\varvec{e}}}}_j^\mathrm{T}Y_l^\mathrm{T}{\hat{{{\varvec{u}}}}}^\star = 0\} = \emptyset$ is violated.

**Fig. 7:**
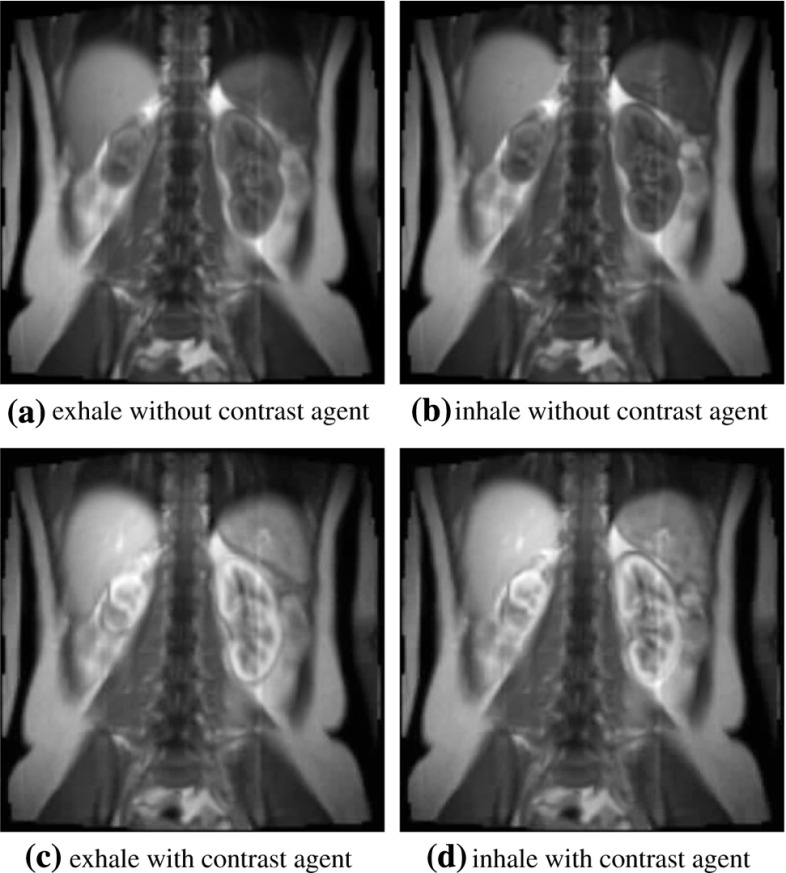
Representative stages of the DCE-MRI sequence: exhale and inhale with and without contrast agent, where these are defined by (9.1)

**Fig. 8:**
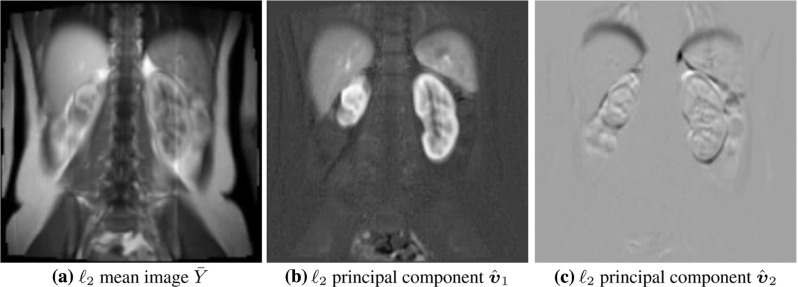
**a** Mean image and **b**–**c** the first two principal components of the DCE-MRI sequence obtained by $\ell _2$ methods. Intensity changes in the image sequence associated with contrast agent and with physiological motion are conspicuously apparent in the components ${\hat{{{\varvec{v}}}}}_1$ and ${\hat{{{\varvec{v}}}}}_2$, respectively

**Fig. 9:**
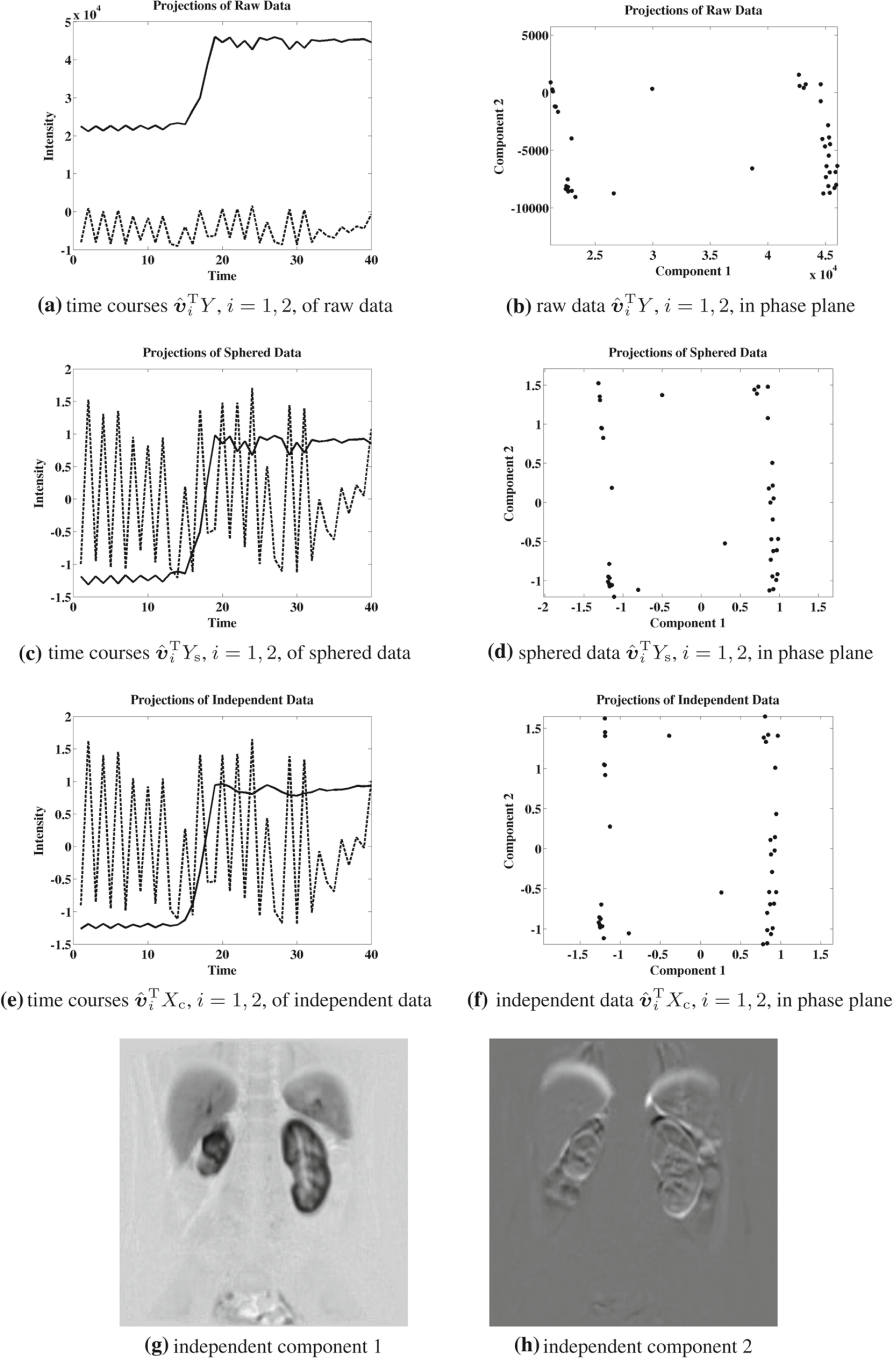
Representation of components of raw, sphered and independent data for the DCE-MRI sequence. These have been determined by $\ell _2$-based methods

## Application to DCE-MRI Sequences

In this section, the proposed methods are applied to the dynamic contrast-enhanced magnetic resonance image (DCE-MRI) sequence http://math.uni-graz.at/keeling/manuskripten/dcemri.mpg to separate intensity changes due to physiological motion from those due to contrast agent. With such a separation, unavoidable physiological motion may be removed in order to investigate tissues in a stationary state. See also [20] and [22]. To focus on the period in which contrast agent arrives in the imaged tissues, only the first 40 of 134 frames are used for the following decompositions. Each frame consists of an image with $400 \times 400$ pixels. Thus, in the notation of Sect. 2, the data are $Y \in \mathbb {R}^{m\times n}$ with $m=400^2 \gg 40 = n$. For a static display of the DCE-MRI sequence, representative stages are shown in Fig. 7: exhale and inhale, with and without contrast agent. Specifically, with ${\bar{Y}},V,\Lambda$ given by (2.7) and (2.10), the images of Fig. 7 are given by

9.1 $$\begin{aligned} {\bar{Y}}+V\Lambda ^{\frac{1}{2}}{{\varvec{e}}}_{ij}, \quad i,j=\pm 1, \quad {{\varvec{e}}}_{ij}=( i,j,0,\ldots ,0)^\mathrm{T} \end{aligned}$$

and the images ${\bar{Y}}$ and

9.2 $$\begin{aligned} {\hat{{{\varvec{v}}}}}_i = V{\hat{{\varvec{e}}}}_i, \quad i=1,2, \quad ({\hat{{\varvec{e}}}}_i)_j= \delta _{ij}, \end{aligned}$$

are shown in Fig. 8. Brightness changes in relation to the background are seen throughout organs in Fig. 8b, and this suggests that ${\hat{{{\varvec{v}}}}}_1$ represents intensity changes in the DCE-MRI sequence due to contrast agent. On the other hand, brightness changes are seen mainly on the edges of organs in Fig. 8c, and this suggests that ${\hat{{{\varvec{v}}}}}_2$ represents intensity changes in the DCE-MRI sequence due to physiological motion. The image sequence is shown more dynamically in Fig. 9. The graphs in the left column are the time courses ${\hat{{{\varvec{v}}}}}_i^\mathrm{T}Y$, ${\hat{{{\varvec{v}}}}}_i^\mathrm{T}Y_\mathrm{s}$, ${\hat{{{\varvec{v}}}}}_i^\mathrm{T}X_\mathrm{c}$, $i=1,2$, for the raw, sphered and independent data, respectively, where $Y_\mathrm{s}$ and $X_\mathrm{c}$ are given by (2.8) and (2.14), respectively. The graphs in the right column are corresponding plots in a phase plane. To determine the most significant independent components, all but the top two principal components were discarded. Then *V* was replaced by its first two columns, $Y_\mathrm{s}$ by its first two rows and $\Lambda$ by a diagonal matrix containing the largest two eigenvalues. Also, the independent images

9.3 $$\begin{aligned} V\Lambda ^{\frac{1}{2}}U^\mathrm{T}Q_iX_\mathrm{c}, \quad i=1,2, \quad Q_i=\text{ diag }\{{\hat{{\varvec{e}}}}_i\}, \quad ({\hat{{\varvec{e}}}}_i)_j = \delta _{ij},\nonumber \\ \end{aligned}$$

are shown in Fig. 9g, h. As explained in connection with Fig. 8, these can be associated respectively with intensity changes in the DCE-MRI sequence due to contrast agent and to physiological motion. Note that there are only small differences between Figs. 9c and e, between Figs. 9d and f, between Figs. 8b and 9g and between Figs. 8c and 9h. Thus, the separation of intensity changes due to physiological motion from those due to contrast agent is achieved here already with the sphered data. Hence the transformation to independent data had little effect for this particular example. Recall from Sect. 2 that the order and the sign of ICA components are not uniquely determined.

**Fig. 10:**
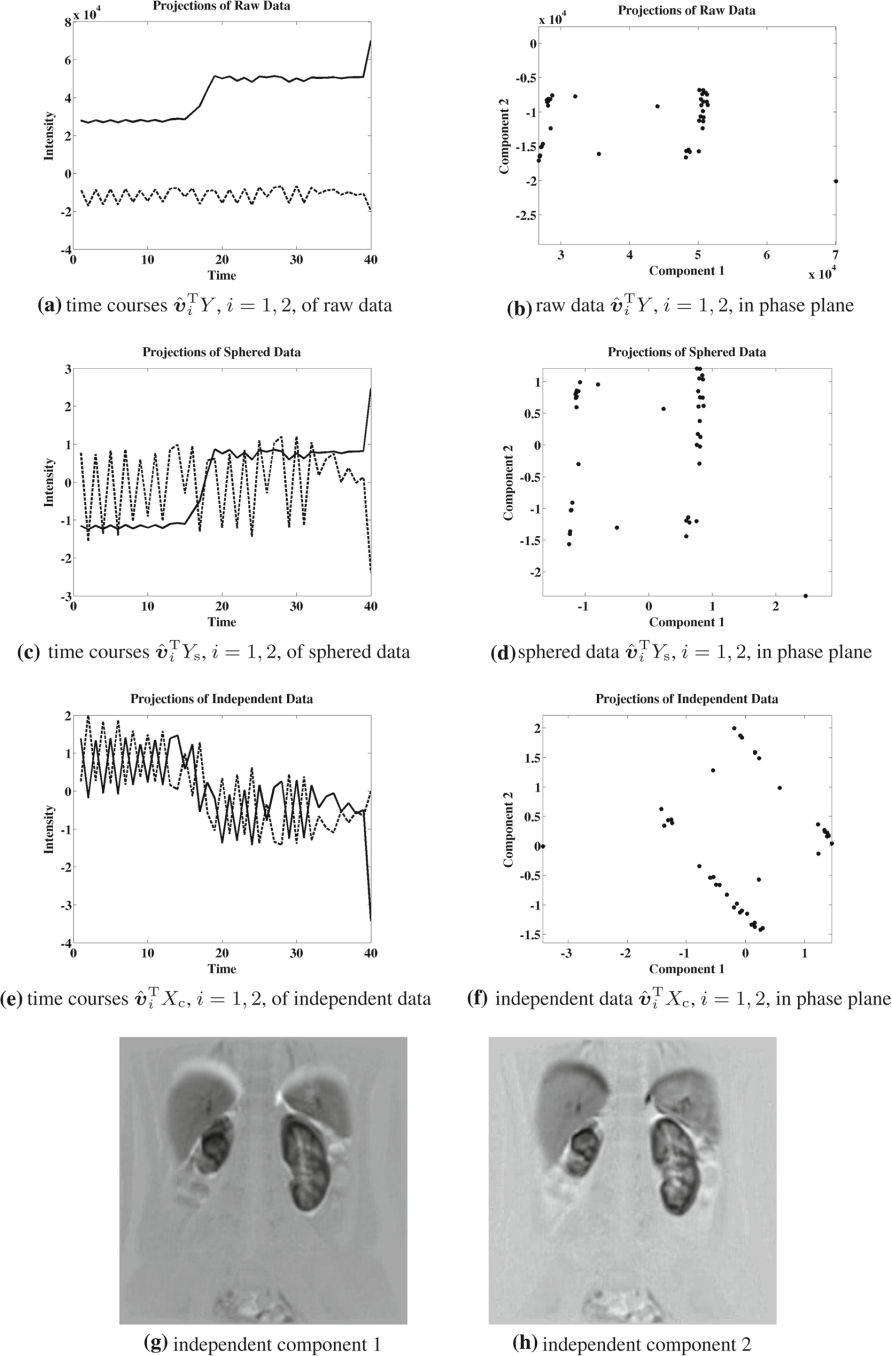
Representation of components of raw, sphered and independent data for the DCE-MRI sequence with a single outlier introduced at the final time. These have been determined by $\ell _2$-based methods

This separation will now be considered in the presence of outliers. As seen in the full DCE-MRI sequence, an excessively bright frame may appear suddenly. To simulate this effect, intensities of the final frame of the sequence are increased by a constant factor. Then the same methods used for Fig. 9 are applied to the corrupted data, and the results are shown in Fig. 10 with the same format as that used in Fig. 9. The corrupted data may be seen at the final time shown in the graphs of the first column of Fig. 10. Also the outlier is conspicuous in the phase plane graphs in the right column of Fig. 10. Finally, the images defined by (9.3) with the corrupted data are shown in Figs. 10g and h. Since these clearly differ from their counterparts in Figs. 9g and h, the presence of the single outlier has corrupted the separation of intensity changes due to physiological motion from those due to contrast agent. We would like to report that we obtained results comparable to those seen in Fig. 10 by applying the FastICA code [13] using optional robust merit functions.

**Fig. 11:**
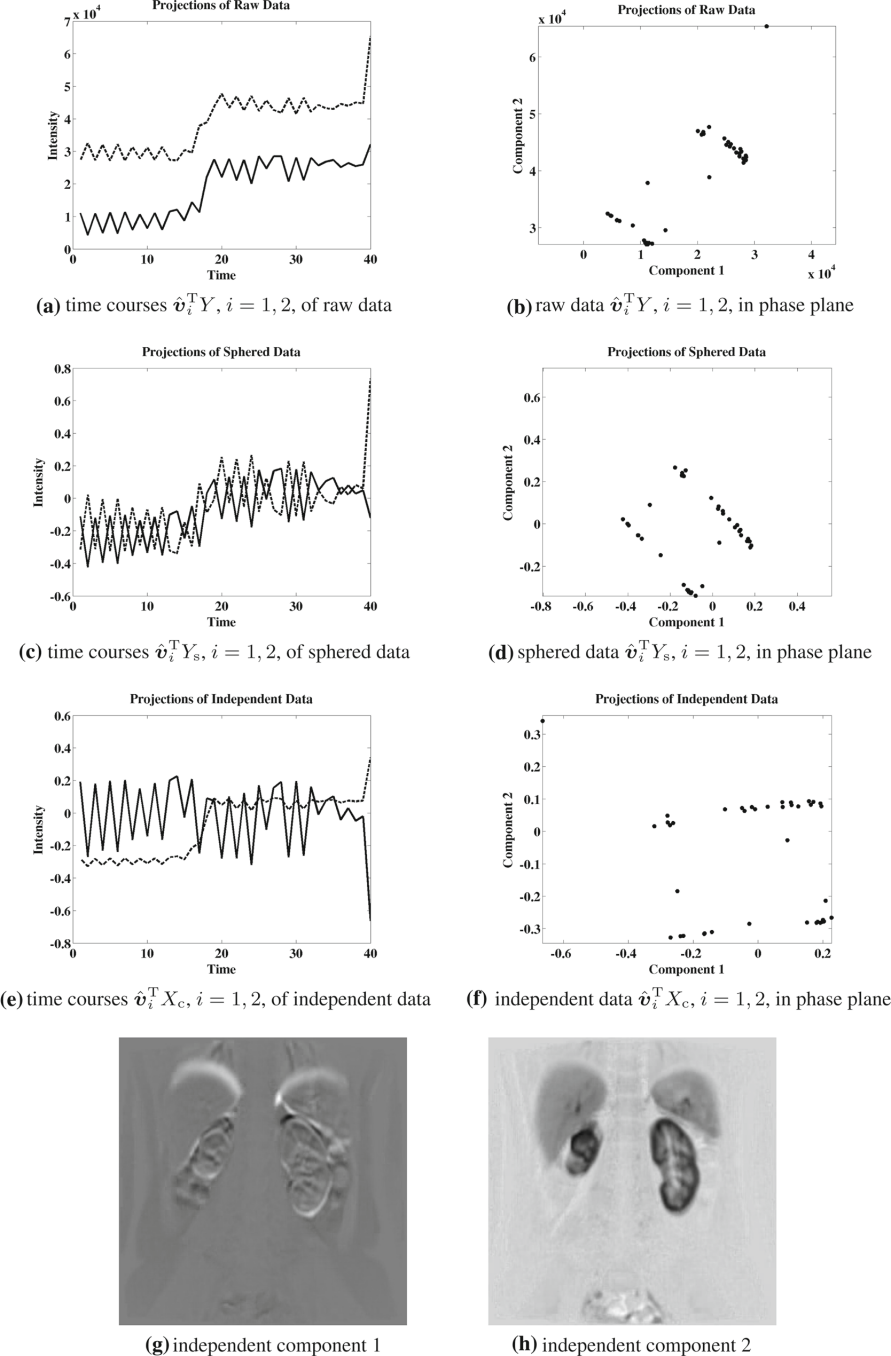
Representation of components of raw, sphered and independent data for the DCE-MRI sequence with a single outlier introduced at the final time. These have been determined by $\ell _1$-based methods

For comparison, the $\ell _1$-based methods of Sects. 3–5 are now applied to the corrupted data, and the results are shown in Fig. 11 with the same format as that used in Figs. 9 and 10. Now the matrices $\overline{V}$, $Y_\mathrm{c}$, *V*, $\Lambda$, $Y_\mathrm{s}$, and *U* are understood as explained in Sects. 3–5 as well as in Remark 2. As in the previous cases, all but the top two principal components are discarded and the respective matrices are reduced correspondingly to have only two rows or columns or both. Then (9.2) and (9.3) apply with these $\ell _1$-based matrices. As before, the corrupted data may be seen at the final time shown in the graphs of the first column of Fig. 11. Also the outlier is conspicuous in the phase plane graphs in the right column of Fig. 11. Finally, the images defined by (9.3) with the corrupted data are shown in Figs. 11g and h. Note the similarities between Figs. 11e–h and their counterparts in Figs. 9e–h. On this basis, the $\ell _1$ methods can be seen to have successfully separated intensity changes due to physiological motion from those due to contrast agent in spite of the outlier. With this separation, the data are projected onto the single independent component of Fig. 11g using (2.21) to produce the following transformed DCE-MRI sequence manifesting contrast changes free of physiological motion,

http://math.uni-graz.at/keeling/manuskripten/dcemri_ica.mpg.

## Conclusion

In this work, robust measures have been introduced for centering complex data in the presence of outliers and for determining principal and independent components of such data. The approach to centering is to use the geometric median. The approach for determining principal components is to find best fit lines through the data, where each line minimizes the sum of distances (not squared) to data points in the subspace orthogonal to other components. The approach for determining independent components is first to sphere the data so that the corresponding axes are aligned with clusters, and then to determine independent axes as those which separate sphered clusters as much as possible. This separation is accomplished by maximizing an $\ell _1$ counterpart to Rayleigh quotients. To optimize the respective merit functions, iterative methods were proposed and their local convergence was proved. Illustrative examples were presented to demonstrate the benefits of the robust approaches. Finally, the proposed methods were applied to a DCE-MRI sequence to separate intensity changes due to physiological motion from those due to contrast agent, and benefits of the robust methods have been demonstrated with respect to this realistic example.
